# Supplementary material for: Data-driven approaches linking wastewater and source estimation hazardous waste for environmental management
Source: Nat Commun. 2024 Jun 26;15:5432. doi: 10.1038/s41467-024-49817-6 (PMC11208539; doi:10.1038/s41467-024-49817-6)
Supplement: Supplementary file 1 — Supplementary Information [file 41467_2024_49817_MOESM1_ESM.pdf]

Supplementary Information for

Data-driven approaches linking wastewater and source estimation hazardous waste for environmental management

Wenjun Xie [a], Qingyuan Yu [a], Wen Fang [a,*], Xiaoge Zhang [b], Jinghua Geng [a], Jiayi Tang [a], Wenfei Jing [a], Miaomiao Liu [a,*], Zongwei Ma [a], Jianxun Yang [a], Jun Bi [a,*]

[a] *State Key Laboratory of Pollution Control and Resource Reuse, School of the Environment, Nanjing University, Jiangsu 210023, China*
[b] *Department of Industrial and Systems Engineering, The Hong Kong Polytechnic University, Kowloon, Hong Kong*

** Corresponding authors, wenfang@nju.edu.cn, liumm@nju.edu.cn, and jbi@nju.edu.cn*

This file includes:

Supplementary texts 1 to 9

Supplementary Figures 1 to 21

Supplementary Tables 1 to 28

SI References

Supplementary Text 1. Data description for three major cases application in other regions and sectors.

Three cases we have developed to demonstrate the generalizability of the model framework are metal surface treatment in Shandong province, metal surface treatment in Zhejiang province and lead and zinc metallurgy in Hunan province. These three study regions were large producers of hazardous waste with Shandong ranking 1st (9,333,500 tons of hazardous waste), Zhejiang ranking 5th (4,447,900 tons of hazardous waste), and Hunan ranking 12th (2,186,300 tons of hazardous waste) in China in 2020[1]. Due to the data availability, these three cases were developed using the HW generation and wastewater data with time-resolution of year from the China Environmental Statistics Database of 2015.

Regarding the first two cases for the sector of metal surface treatment, 14 variables, including the firm scale, 6 manufacturing processes (metal heat treatment, metal surface processing, circuit board treatment, equipment maintenance, exhaust gas treatment, and wastewater treatment), and 7 variables of wastewater monitoring indicators (wastewater discharge amount, COD, NH_3-N, N, P, Cr, and Cr^{VI}), were adopted as input features to build the model. The response variable was the total generation quantity of HW. Note that the firm scale classification used in the China Environmental Statistics Database differs from the method in this study. The database categorizes firm scale based on the number of staff and business revenue into four groups. After data processing, there were 190 observations for the first case and 396 observations for the second case. Regarding the third case for the sector of lead and zinc metallurgy, 12 variables, including the firm scale, 4 manufacturing processes (mineral leaching, electrolysis, roasting, and flue gas treatment), and 7 variables of wastewater monitoring indicators (wastewater discharge amount, COD, NH_3-N, N, P, Cr, and Cr^{VI}), were adopted as input features to build the model. The response variable was the total generation quantity of HW. After data processing, there were 102 observations for the third case. The descriptive statistics of numeric variables were shown in Supplementary Table 15 to 17.

Supplementary Text 2. Missing value imputation.

There were some missing values for the features of water contaminate emission (chemical oxygen demand (COD), pH, ammonia nitrogen (NH_3-N), total nitrogen (N), total phosphorus (P), iron (Fe), total chromium (Cr), hexavalent chromium (Cr^{VI}), copper (Cu), zinc (Zn), and nickel (Ni)) and wastewater pH in the raw dataset collected from automatic IoT sensors (Table S21).

During missing value imputation, the data leakage management was applied through splitting the data into training and testing sets before performing any preprocessing steps. The training dataset was considered as known data, and the missing values were imputed based on the information from the known data rather than the whole raw dataset[2].

Regarding the training dataset, for the missing value of the variables of wastewater routine monitoring indicators (COD, NH_3-N, N, and P), it was imputed by multiplying the contaminant emission intensity by the wastewater discharge amount. In detail, the contaminant emission intensity, referring to the contaminant emission per wastewater flow, was the median value of each firm. If the enterprise had no records of this indicator, the median value at the industrial sectors level was used. Similarly, the missing value for wastewater pH was imputed by the median value of the enterprise or the industrial sector.

However, regarding the 6 metal emission indicators (iron (Fe), total chromium (Cr), hexavalent chromium (Cr^{VI}), copper (Cu), zinc (Zn), and nickel (Ni)), if there is missing value, it is regarded that this firm will not generate this metal contaminant during manufacturing process and then we set these missing values as 0.

In terms of the testing dataset, the missing value imputation strategy is similar to the training dataset, but the median value of contaminant emission intensity and wastewater pH was calculated based on the training dataset.

Supplementary Text 3. Data sources and quality control.

The data of employee number and industrial sector were officially from the Department of Ecology and Environment of Jiangsu Province. The data of manufacturing processes for each enterprise were determined through combing the typical processes related to HW generation for each sector (Table S3) and the information declared by enterprises. The daily data of water contaminant emission was monitored by automatic IoT sensors. The HW generation data with time-resolution of month was declared by the enterprises.

To ensure the reliability of the HW generation data and manufacturing processes data which were declared by enterprises, the data from those enterprises which were found to have records of environmental violations and administrative penalties were eliminated. The records of environmental enforcement for each enterprise were officially from the department of ecology and environment of Jiangsu Province.

The data used to investigate the characteristics of HW generation from different industrial sectors across China in 2015 were from the China Environmental Statistics Database. This database is considered the most authoritative and reliable national environmental survey database in China. The data quality in this database is ensured by laws and regulations issued by China's Ministry of Ecology and Environment, as well as regular monitoring and unannounced on-site surveys by local ecology and environment bureaus[3].

Supplementary Text 4. Outlier rejection.

After data aggregation, splitting, and missing value imputation, an outlier screening and removal procedure was conducted on the original training dataset (13879). After outlier rejection at the ratio of 5%, a final training dataset with 13,183 observations. The outlier rejection model was adopted for the observations from each industrial sector, respectively. The outlier reject model involved 6 unsupervised machine learning algorithms of K Nearest Neighbors[4,5], Minimum Covariance Determinant[6,7], Clustering-Based Local Outlier Factor[8,9], Histogram-based Outlier Score[10], Local Outlier Factor[11,12] and Isolation Forest[13,14]. Each algorithm could calculate an outlier score for each observation and the score was standardized using the z-score normalization. Scores from 6 algorithms was aggregated using the Average of Maximum (AOM) strategy to determine the final score [15]. AOM has been recommended for detector combination due to its combined bias and variance reduction capability and thus outperform than other methods, such as using the average and maximum score. Specifically, AOM method randomly divided six base detectors into three subgroups and take the maximum score for each subgroup. Then the final score was the average of all subgroup scores. A higher outlier score indicated that the sample deviates far from the overall distribution of the observations. Anomalies were then sorted based on their final outlier scores. The top 5% of the data samples were considered as outliers and removed from the dataset.

Supplementary Text 5. Observations description.

The 16,477 observations used to build and test model after data processing were from 1024 enterprises, which means that each enterprise has about 16 observations in average. For these observations from the same enterprise, their value of enterprise' static characteristics, such as firm scale and manufacturing processes, kept constant, but the data of real-time manufacturing activities, mainly referring to the wastewater data, varied because different observations indicated the enterprise' behavior at different sampling time on a monthly basis.

Supplementary Text 6. Description of eight artificial intelligent algorithms used to develop models

To screen the optimized base algorithm for model development, eight algorithms, including five machine learning algorithms and three deep learning algorithms, were adopted during constructing the combined model. These 5 machine learning algorithms are gradient boosting decision tree (GBDT), support vector machine (SVM), extreme gradient boosting (XGBoost), k-nearest neighbor (kNN), and random forest (RF). These 3 deep learning algorithms suitable for fitting tabular data are Multilayer Perceptron (MLP), MLP ensemble, and Tabular neural network (TNN).

GBDT combines the gradient boosting and decision tree algorithms. It is an additive model that fits new models to provide more accurate estimates of the response variable[16]. The core idea behind GBDT is to construct a new base learner that minimizes the steepest descent of the loss function. GBDT utilizes decision trees as weak learners, and aggregates the results of all trees to obtain the final result. The trees in GBDT are grown to a maximum depth specified by the user, and the terminal leaves are named once the trees are built. The response of trees is calculated to minimize the loss function for all samples in each leaf, and the outputs are summed into the additive model weighted by a learning rate. The learning rate can improve the model performance by scaling the contribution from new trees.

SVM works by finding the optimal hyperplane to make regression predictions that closely fits the training data points while keeping the distance between the hyperplane and the data points as small as possible. The goal of SVM is to minimize the error between predicted and actual values while maximizing the margin of the hyperplane[17]. SVM can avoid overlearning, local minima and dimensional catastrophe problems.

XGBoost was proposed to address the limitations of weakly supervised learning[18]. It develops strong learners through an additive process that includes a learning phase fitting the entire input data. This phase is repeated multiple times until overfitting is reduced. The objective function (eq. 1 and 2) treats the model as a combination of weak learners. XGBoost is a machine learning algorithm that builds powerful models by adding weak learners in an iterative manner. The algorithm fits the entire input data in a learning phase that is repeated multiple times until a stopping criterion is met. The additive process includes adaptive mechanisms to reduce overfitting. The objective function (eq. 1 and 2) treats model complexity as a regularization term and adds it to the cost function.

$$\text{obj} = \sum\nolimits_i L\left(y_i, F\left(x_i\right)\right) + \sum\nolimits_t \varphi\left(f_t\right) \tag{1}$$

$$\varphi\left(f\right) = \gamma T + \frac{1}{2}\theta \sum\nolimits_{\mathrm{j}}^{T} \omega_j^2 \tag{2}$$

where T denotes the number of leaves of the tree f, and ω is the score of a leaf j of the tree f; Tree f(x) is defined as f(x) = ω_q(x), where q is the tree structure that maps a sample x to its corresponding leaf; θ is the regularization parameter, which is a threshold of the score function improvement to continue splitting the tree.

KNN is a non-parametric method used for classification and regression tasks[19]. It considers the K closest training examples in the feature space. In regression, it outputs the attribute value, which is the average of the K nearest

neighbor values. KNN predicts the value of a new data point based on its similarity to each training point in the dataset, using 'feature similarity'. The similarity between the new point and each training point is quantified using the Euclidean distance (eq 3). To assign a value to the new point, a subset of neighbors is selected based on their closest Euclidean distance to the new point. The number of neighbors chosen is determined by the value of K. The final prediction for the new point is the average value of these selected neighbors.

$$D_E = \sqrt{\sum_{i=1}^{k} (x_i - y_i)^2} \quad (3)$$

Random Forest is an ensemble model that consists of a network of decision trees. It uses bootstrapping techniques to create random datasets for training the decision tree ensemble. Bootstrapping involves replacing the original dataset with different training datasets to train each tree, thereby increasing diversity among the trees in the forest. The dataset for training is divided into two subsets, and the decision rules for binary splits in regression trees are optimized by minimizing the sum of squared variances. The prediction for each observation is estimated by averaging the predictions from the trees[20].

MLP consists of fully connected neurons with a nonlinear kind of activation function, organized in at least three layers[21]. It is usually trained using the backpropagation method, including two main processes of forward and backward. In the forward process, a neuron's output is calculated by three steps: the weighting step of multiplying each input feature value by its weight, the sum step of adding them together, and the transfer step of applying an activation function to the sum value[22]. In the backward propagation, the connection weights between neurons are optimized based on the error in the output compared to the expected result. In our study, Mean Squared Error (MSE) loss function and Cross Entropy (CE) loss function are utilized to measure the error mentioned above for regressors and classifiers, respectively:

$$\text{MSE Loss} = \frac{1}{n} \sum_{i=1}^{n} \left(y_i - \widehat{y_i} \right)^2 \quad (4)$$

$$\text{CE Loss} = \frac{1}{n} y_i \bullet \log \widehat{y_i} \quad (5)$$

where n is the number of data points in a batch; y_i refer to the observed generation quantity of HW in eq.4 and whether HW is generated in eq.5, respectively. $\hat{y}_i$ are the predicted generation quantity or the probability of HW generation.

The MLP ensemble method leverages multiple MLPs for ensemble learning. Specifically, we built 5 MLPs and used the bagging strategy[23] to aggregate their output. In detail, for the regression model, the predicted value from all the MLP base models were averaged. For the classification model, the dominant classification among the predictions from 5 base MLP models was used as the final result.

Tabular neural network is a special MLP that adopts embedding layers to handle sparse and high-dimensional categorical features[24,25]. Embedding layer is widely used in natural language processing (NLP) and recommendation systems to make up for the critical flaw of one-hot word representation: data-sparse problem and loss of semantic relatedness between words[26]. Specifically, the embedding layer is a learnable dense layer that converts one-hot input into low-dimensional dense vectors as features of the downstream network. Except

for the embedding layer, Tabular neural network also uses many other approaches like dropout[27] and Kaiming initialization[28] to improve the performance of the model.

Supplementary Text 7. Metrics to evaluate the performance of regression and classification models.

Regression models were evaluated using multiple metrics of the coefficient of determination (R^2), root-mean-square-error (RMSE), mean absolute error (MAE), mean absolute percentage error (MAPE), median absolute deviation (MAD), mean square error (MSE) and sum of squares due to error (SSE)[29,30]. Their respective equations are presented below:

$$R^2 = 1 - \frac{\sum_{i=1}^{m}(y_{res}^i - y_{tru}^i)^2}{\sum_{i=1}^{m}(\overline{y_{tru}} - y_{tru}^i)^2} \tag{6}$$

$$\text{RMSE} = \sqrt{\frac{1}{m}\sum_{i=1}^{m}(y_{tru}^i - y_{res}^i)^2} \tag{7}$$

$$\text{MAD} = \frac{1}{m}\sum_{i=1}^{m}\left|y_{res}^i - \overline{y_{res}}\right| \tag{8}$$

$$\text{MAE} = \frac{1}{m}\sum_{i=1}^{m}\left|y_{res}^i - y_{tru}^i\right| \tag{9}$$

$$\text{MAPE} = \frac{1}{m}\sum_{i=1}^{m}\left|\frac{y_{res}^i - y_{tru}^i}{y_{tru}^i}\right| \times 100\% \tag{10}$$

$$\text{MSE} = \frac{1}{m}\sum_{i=1}^{m}(y_{tru}^i - y_{res}^i)^2 \tag{11}$$

$$\text{SSE} = \sum_{i=1}^{m}(y_{res}^i - y_{tru}^i)^2 \tag{12}$$

where m is the amount of data in the test set. y_{tru} and y_{res} are the observed generation quantity of hazardous waste and the predicted generation quantity of hazardous waste by the model, respectively. $\overline{y_{tru}}$ and $\overline{y_{res}}$ are the average values of the generation quantity of hazardous waste of y_{tru} and y_{res}.

Classification models were evaluated using metrics of accuracy, recall, precision, and F1-score (F1).[30]. Their respective equations are presented below:

$$\text{Accuracy} = \frac{\text{TP} + \text{TN}}{\text{TP} + \text{TN} + \text{FP} + \text{FN}} \tag{13}$$

$$\text{Precision} = \frac{\text{TP}}{\text{TP} + \text{FP}} \tag{14}$$

$$\text{Recall} = \frac{\text{TP}}{\text{TP} + \text{FN}} \tag{15}$$

$$\text{F1} = \frac{2 \times \text{Precision} \times \text{Recall}}{\text{Precision} + \text{Recall}} \tag{16}$$

where TP is the number of positive samples correctly predicted by the model to be in the positive category. TN is the number of negative samples correctly predicted by the model to be in the negative category. FP is the number of negative samples incorrectly predicted by the model to be in the positive category. FN is the number of positive samples incorrectly predicted by the

model to be in the negative category. In this study, the positive category referred to observations with MHW generation quantity > 0.

Supplementary Text 8. Comparison of different missing value imputation approaches on model performance

To further evaluate the missing value imputation approaches on model performance, we compared the strategy adopted in this study with other three methods of missing value imputation based on industrial sector median emissions, KNNimpute, and MissForest algorithm.

The method of missing value imputation on industrial sector medians is to use the median value of the industrial sector to fill the missing values. KNNimpute is an algorithm to impute missing values based on the weighted value of k-nearest neighbor samples. In this study, the data of wastewater routine monitoring indicator from each industrial sector was program inputs and then KNNimpute method was applied to select the k-nearest neighbor samples (k = 5 in this study). Missing values were imputed with the weighted average of the k-nearest neighbor samples. Weights were calculated based on the inverse proportionality to the Euclidean distance of the k-nearest neighbor sample distances from the missing values[31,32].

MissForest algorithm predicted the missing value using the random forest algorithm. To be specific, the data of wastewater routine monitoring indicator from each industrial sector was program inputs and then the missing values were imputed for the variable from least to greatest based on the amounts of missing values. The response variable was the factor X_i that requires imputation, while the predictor variables were other input factors. For predictor variables, their missing value were initially filled using the mean value. Random forest model was trained using the observations with true value and then adopted to predict the missing value. This procedure was repeated until all missing values were imputed[33].

After missing values were filled, combined models to predict the total generation quantity of HW and MHW were trained and evaluated. As shown in Supplementary Table 27, models developed from the data using the imputation methods of multiplying wastewater discharge amount by contaminant emission intensity outperformed the other three methods.

Supplementary Text 9. Comparison of different data balance approaches on model performance.

To further evaluate the data balance approaches on model performance, we compared the strategy adopted in this study with other two data balance techniques of Synthetic Minority Over-Sampling Technique for Regression (SMOTER) and random over-sampling.

SMOTER generates new synthetic examples from the rare cases through an interpolation strategy[34]. This interpolation is carried out using two rare cases (one is a seed case and the other is randomly selected from the k-nearest neighbors of the seed) and the new synthetic sample is determined as a weighted average of the target variable values of the two rare cases. All rare cases are used in turn as seed examples. Random over-sampling means it randomly sampling observations from rare cases and then combining these samples with original rare cases to form the new dataset[35]. Generally, these three data balance techniques performed random undersampling on the normal partition (low generation quantity of HW dataset) and oversampling on the rare partition (high generation quantity of HW dataset), resulting in an equal size of data for the normal and rare partition.

Data balancing algorithms were performed on the training set, and the model performance was compared using the same test set. As shown in Supplementary Table 28, the data balance method of SMOGN outperform than other techniques as evidenced by metrics of both R^2 and RMSE on the testing dataset. However, the random over-sampling technique could not improve the model predictive methods when comparing to the model developed from raw dataset without data balance procedure.

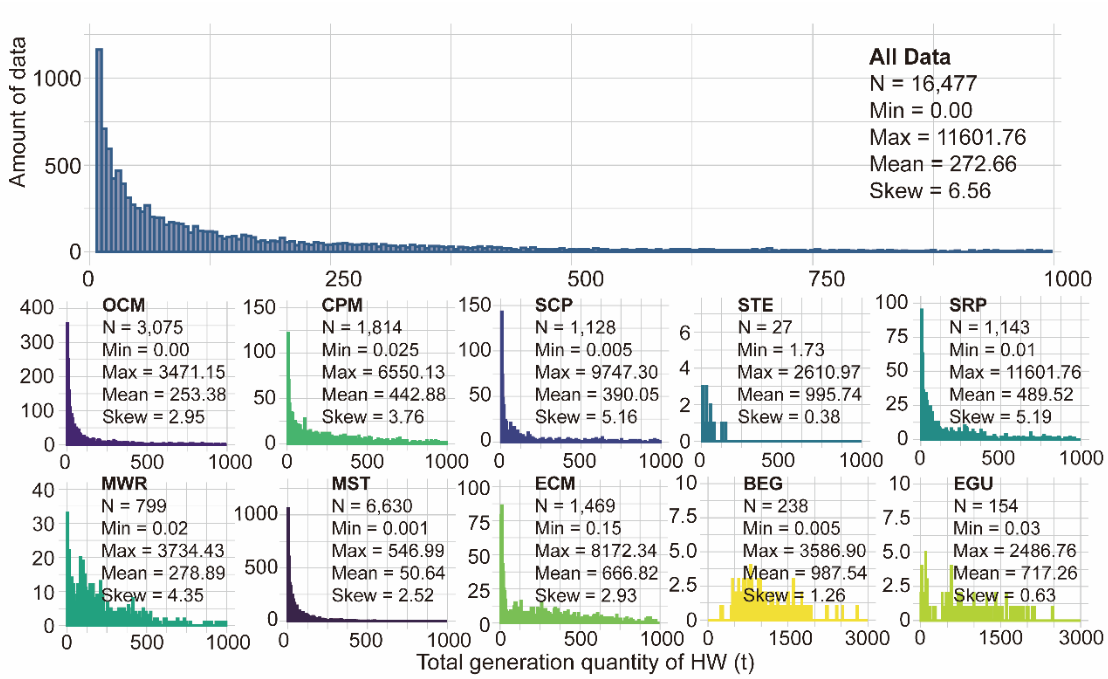

Supplementary Figure 1. Histogram of total generation quantity of HW for all the 16,477 observations and observations in each industrial sectors. 10 industrial sectors involved in the dataset are organic chemical materials manufacture (OCM), chemical pesticides manufacture (CPM), specialized chemical products manufacture (SCP), steel rolling and processing (SRP), metal wire and rope manufacture (MWR), metal surface treatment (MST), electronic circuits manufacture (ECM), biomass energy generation (BEG), and electricity generation using other sources (EGU).

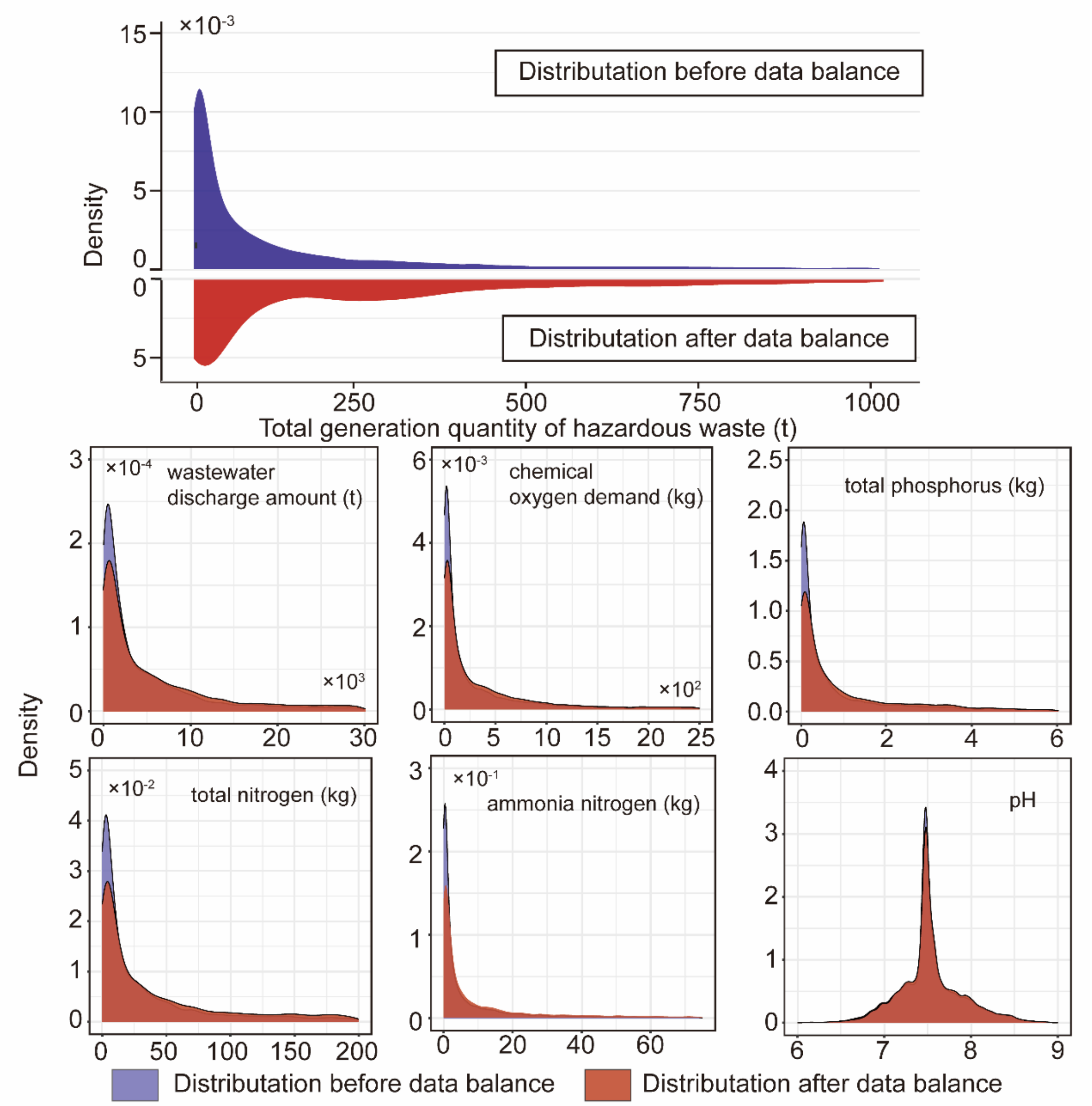

Supplementary Figure 2. Probability density distribution of total generation quantity of HW and 6 water contaminant emission indicators (wastewater discharge amount, COD, P, N, NH_3-N and pH) in the cleaned training dataset before and after data balance.

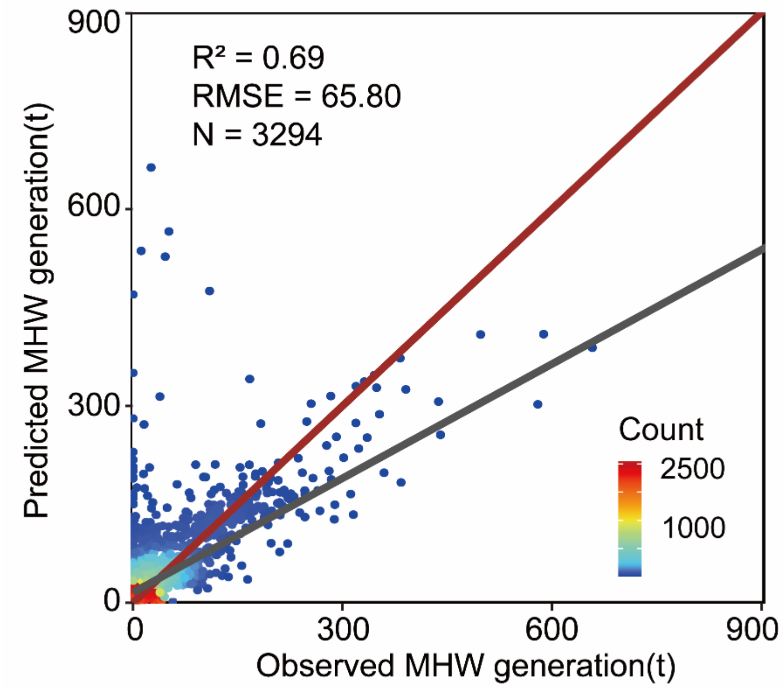

Supplementary Figure 3. Performance of the regression model directly developed from the whole balanced training dataset to predict the generation quantity of MHW.

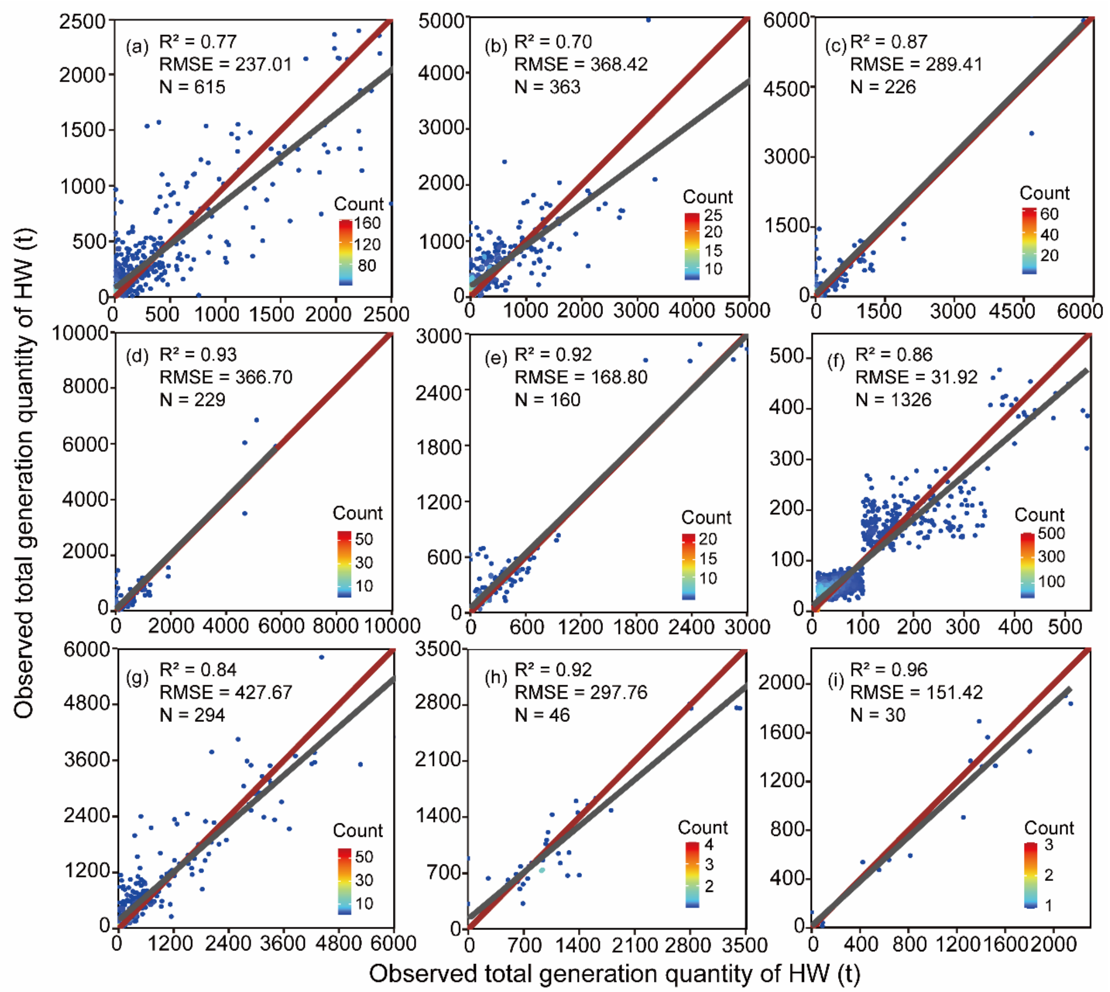

Supplementary Figure 4. Performance of sector-independent models to predict the total generation quantity of HW in the sector of (a) organic chemical materials manufacture (OCM), (b) chemical pesticides manufacture (CPM), (c) specialized chemical products manufacture (SCP), (d) steel rolling and processing (SRP), (e) metal wire and rope manufacture (MWR), (f) metal surface treatment (MST), (g) electronic circuits manufacture (ECM), (h) biomass energy generation (BEG), and (i) electricity generation using other sources (EGU).

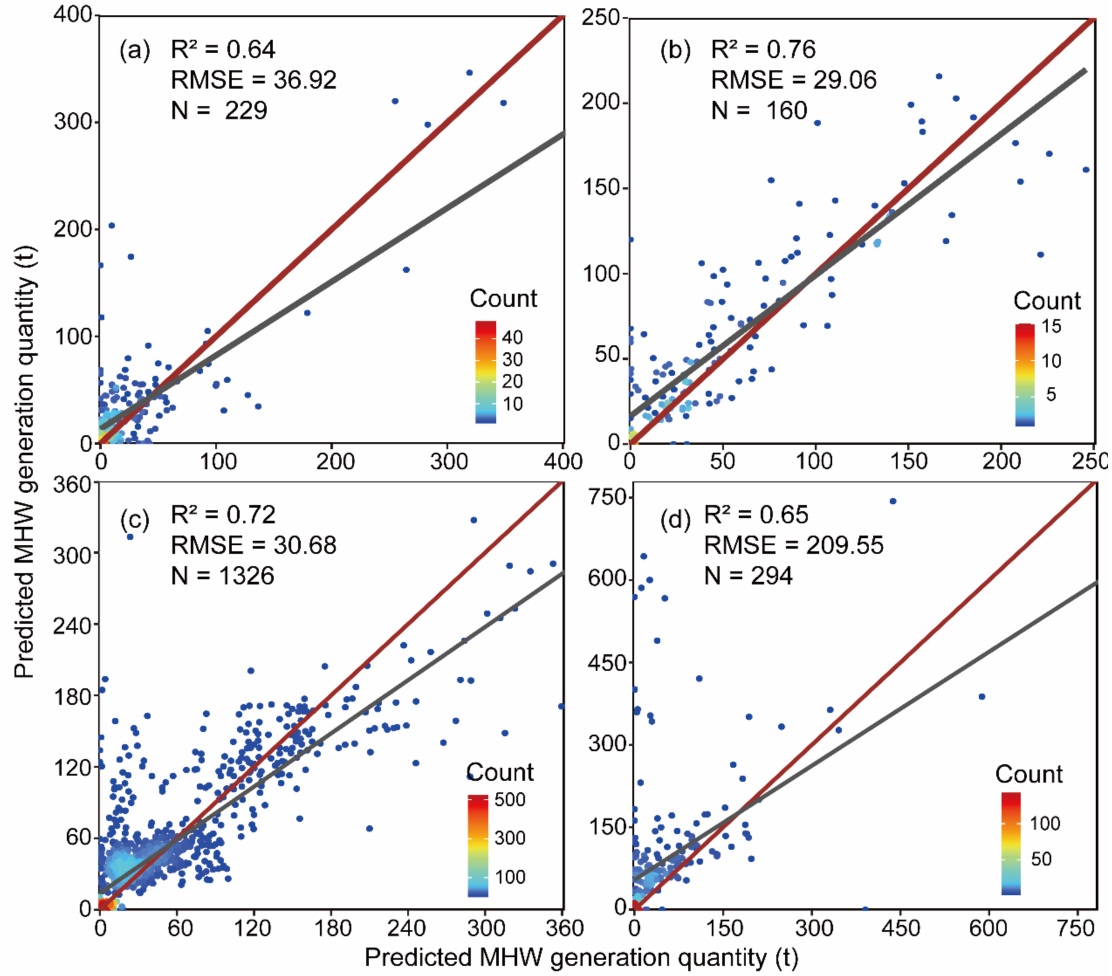

Supplementary Figure 5. Performance of sector-independent models to predict the generation quantity of MHW in the sector of (a) steel rolling and processing (SRP), (b) metal wire and rope manufacture (MWR), (c) metal surface treatment (MST), and (d) electronic circuits manufacture (ECM).

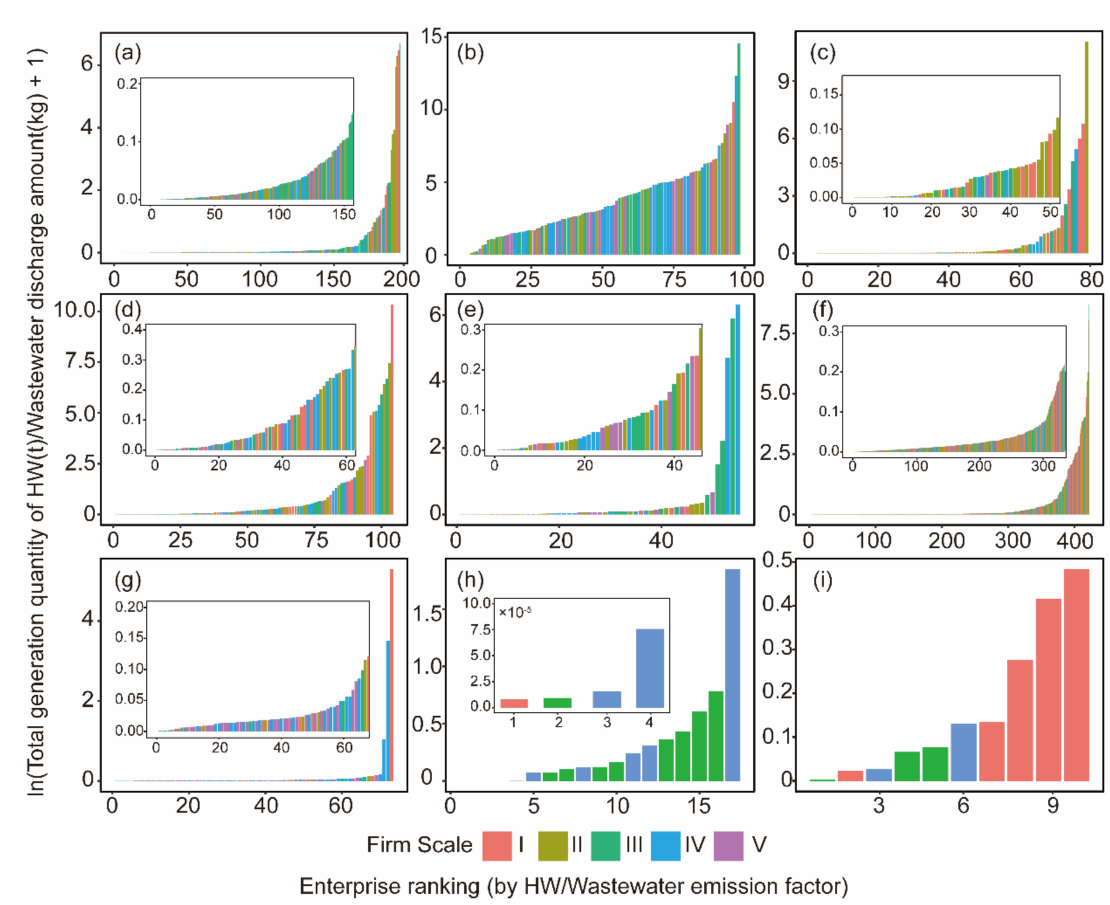

Supplementary Figure 6. The ratio of HW generation quantity to wastewater discharge amount for enterprises in the studied region of Jiangsu. Each bar represents one enterprise. The ratio of HW generation quantity to wastewater discharge amount is calculated by dividing the average monthly HW production of an enterprise from January 2020 to December 2022 by the monthly wastewater discharge. (a)~(i) refer to organic chemical materials manufacture (OCM), chemical pesticides manufacture (CPM), specialized chemical products manufacture (SCP), steel rolling and processing (SRP), metal wire and rope manufacture (MWR), metal surface treatment (MST), electronic circuits manufacture (ECM), biomass energy generation (BEG), and electricity generation using other sources (EGU), respectively.

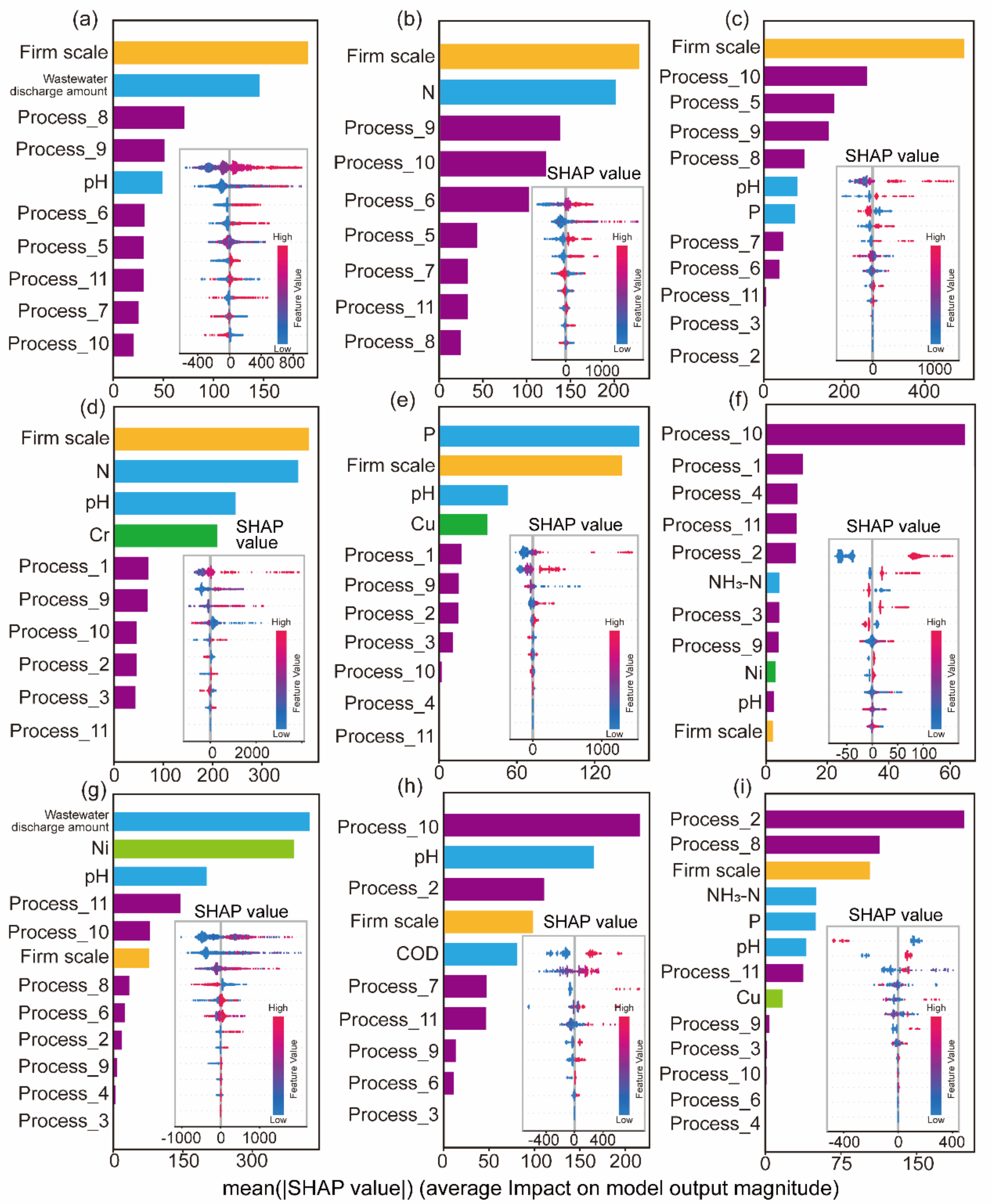

Supplementary Figure 7. Feature importance (histogram plots) and SHAP summary plots for sector-independent models to predict the total generation quantity of HW. (a)~(i) refer to industrial sectors organic chemical materials manufacture (OCM), chemical pesticides manufacture (CPM), specialized chemical products manufacture (SCP), steel rolling and processing (SRP), metal wire and rope manufacture (MWR), metal surface treatment (MST), electronic circuits manufacture (ECM), biomass energy generation (BEG), and electricity generation using other sources (EGU), respectively. In each SHAP summary plot, the features are ranked according to the importance of the features.

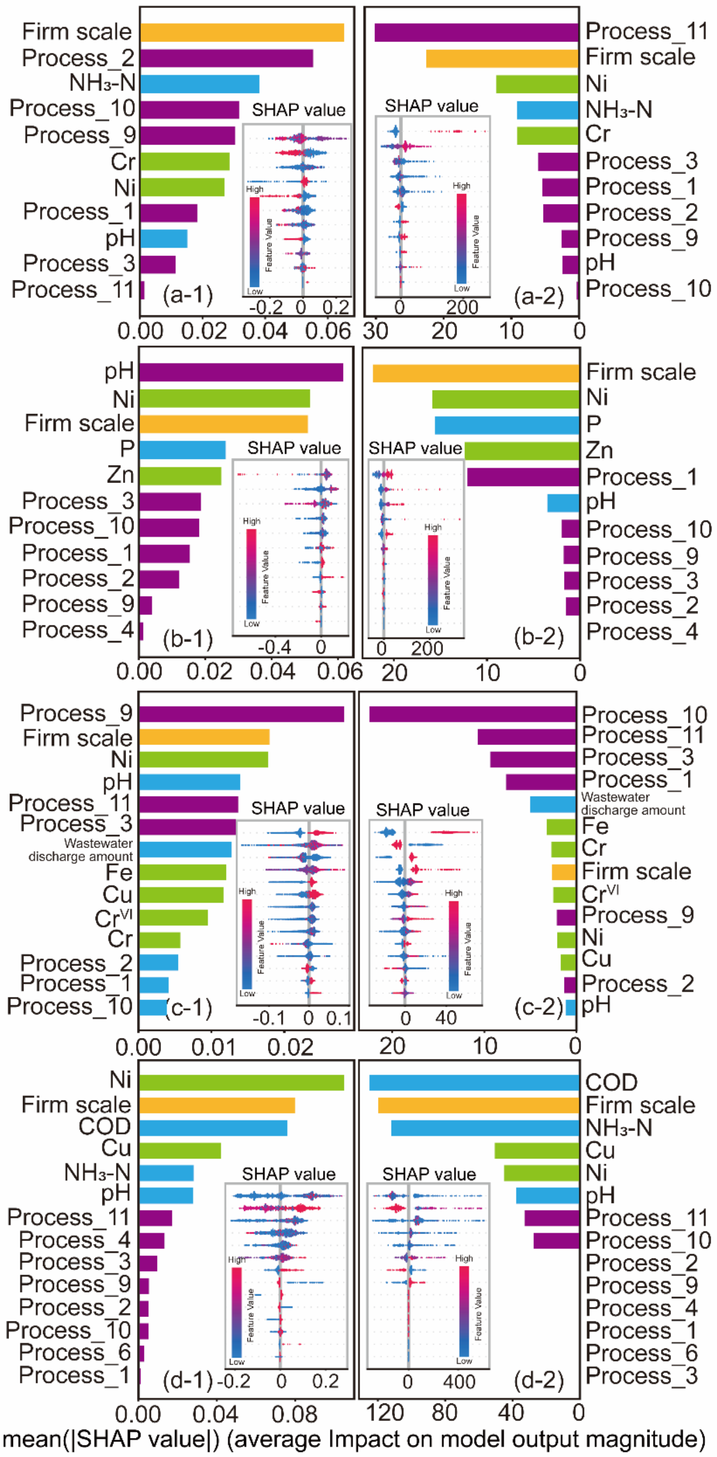

Supplementary Figure 8. Feature importance (histogram plots) and SHAP summary plots for sector-independent ensemble models to predict generation quantity of MHW. (a-1) and (a-2) represent the classification model and regression model in the ensemble model for steel rolling and processing (SRP) sector, respectively. (b)~(d) indicate industrial sectors of metal wire and rope manufacture (MWR), metal surface treatment (MST), and electronic circuits manufacture (ECM), respectively.

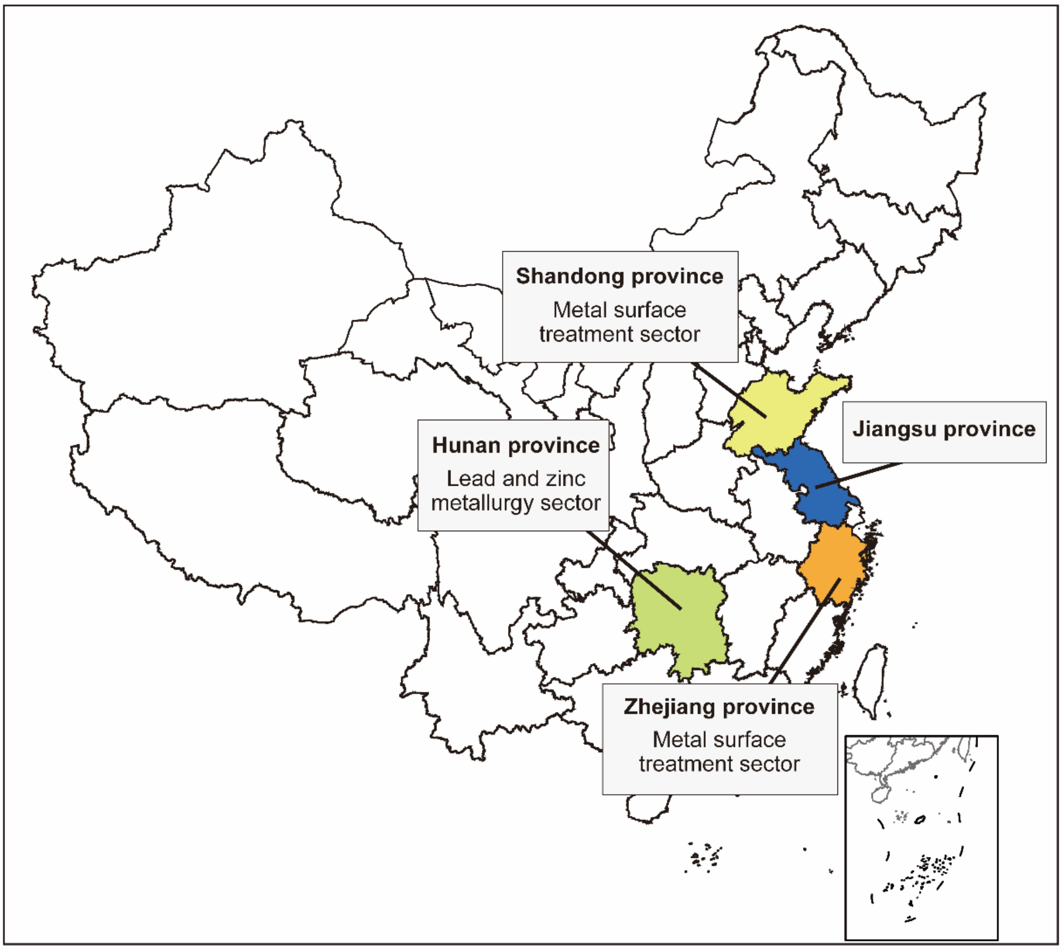

Supplementary Figure 9. Studied regions for three application cases developed to demonstrate the generalization of the model framework.

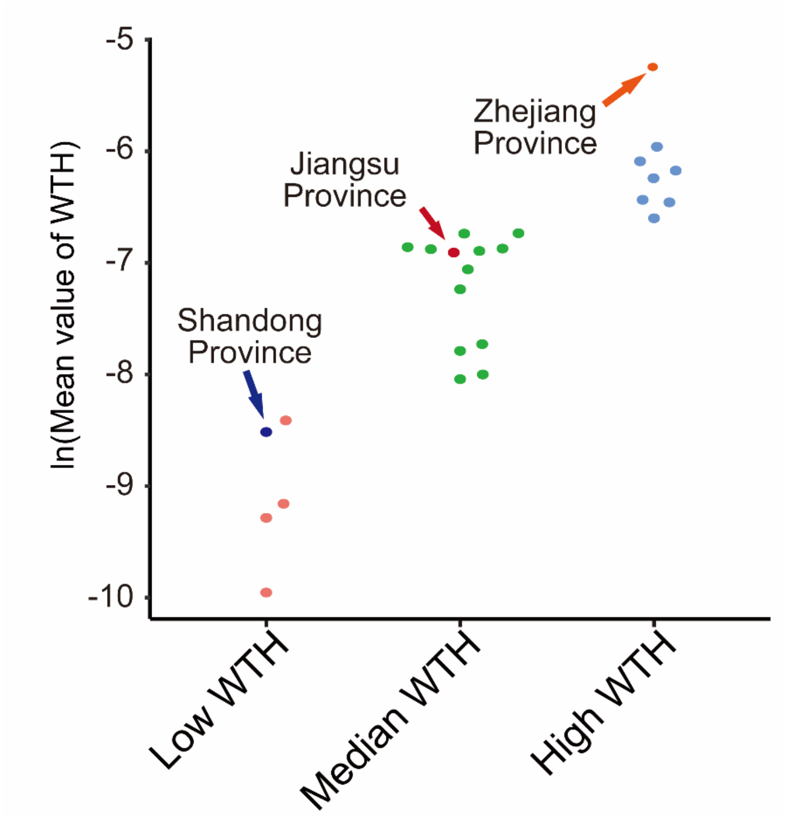

Supplementary Figure 10. The cluster of different regions, determined by K-means, in the ratio of wastewater discharge amount to HW generation quantity (WTH) for the sector of metal surface treatment.

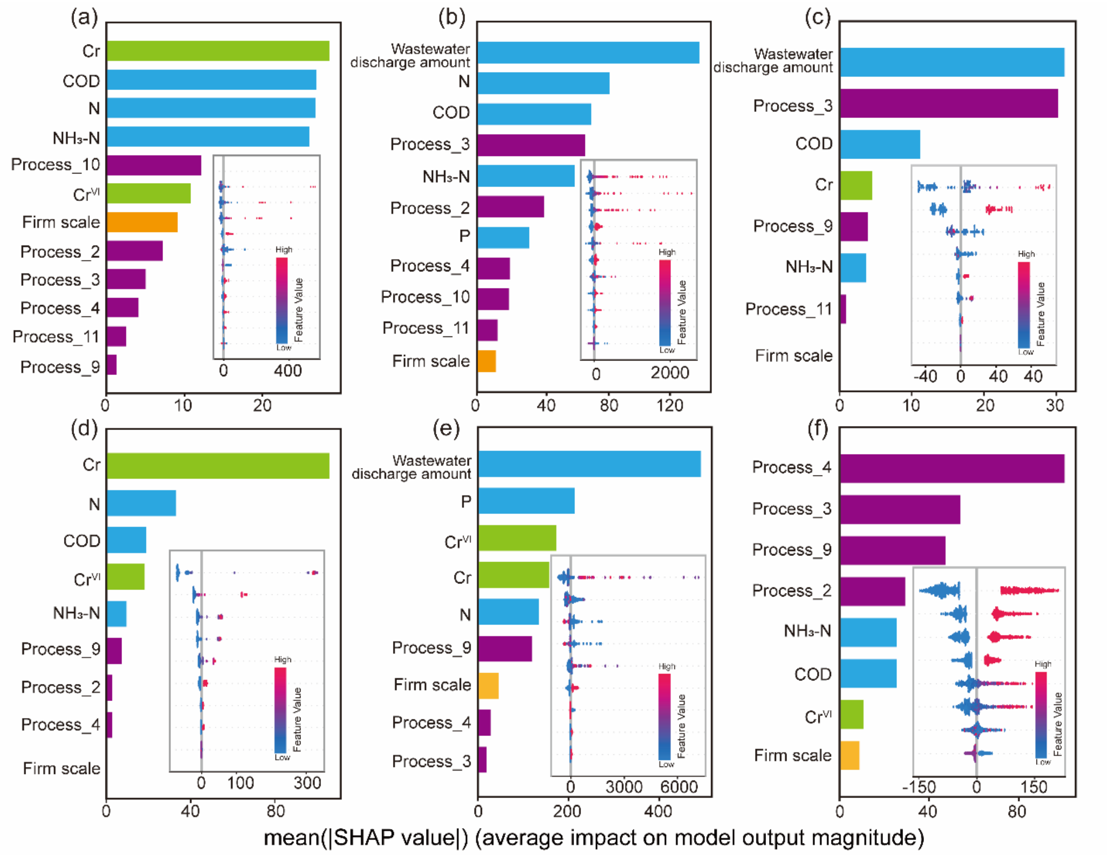

Supplementary Figure 11. Feature importance (histogram plots) and SHAP summary plots for six application cases of the metal surface treatment sector to predict the total generation quantity of HW. (a)~(f) refer to the application regions of Shandong Province, Zhejiang Province, Fujian Province, Hebei Province, Jiangsu Province, and Guangdong Province, respectively. In each SHAP summary plot, the features are ranked according to their importance.

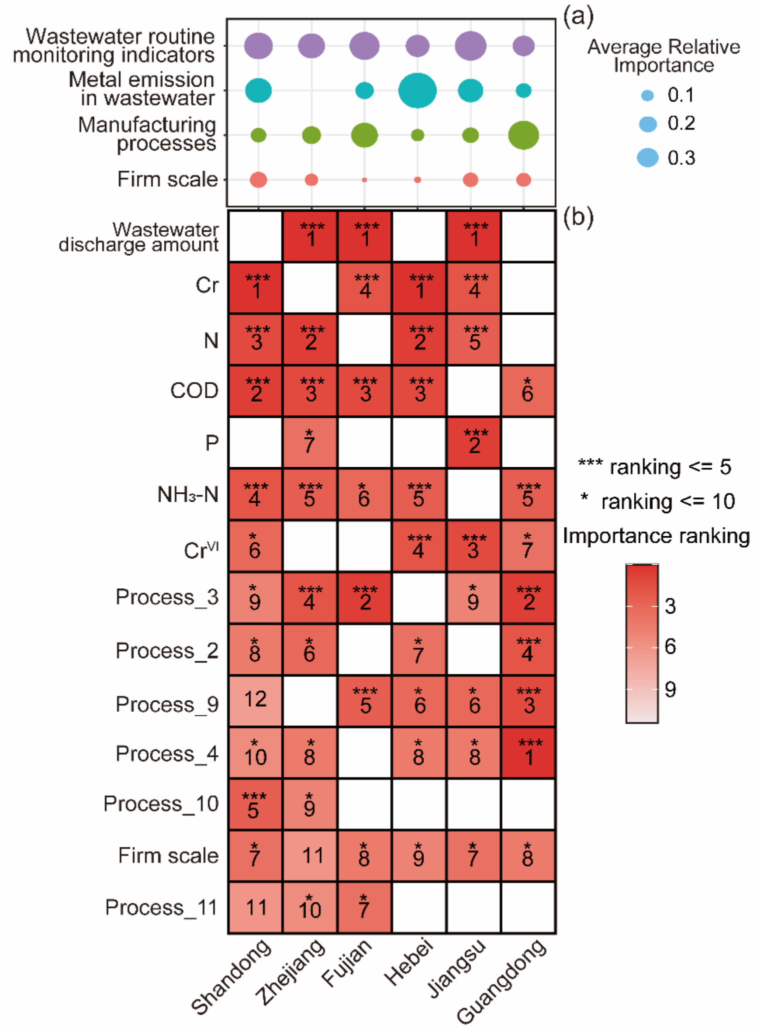

Supplementary Figure 12. Importance of input features for six models of application cases in the metal surface treatment sector to predict the total generation quantity of HW. (a) The average relative importance, indicated by the average MAS value of variables in 4 groups of the firm scale, manufacturing processes, the wastewater routine monitoring indicators, and metal emission in wastewater. (b) Ranking of each input feature importance for 6 models. The number inside each cell reflected the ranking of the variable. Variables ranked in the top 5 are marked with *** to denote their crucial importance in model prediction, while variables ranked between 6th and 10th are marked with * to indicate their relatively significant impact.

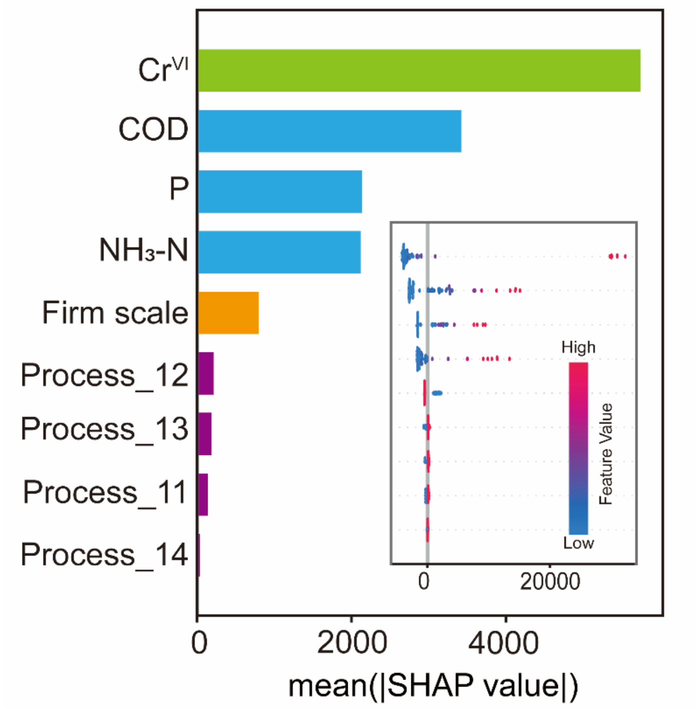

Supplementary Figure 13. Feature importance (histogram plots) and SHAP summary plots for Hunan application cases in the lead and zinc metallurgy to predict the total generation quantity of HW.

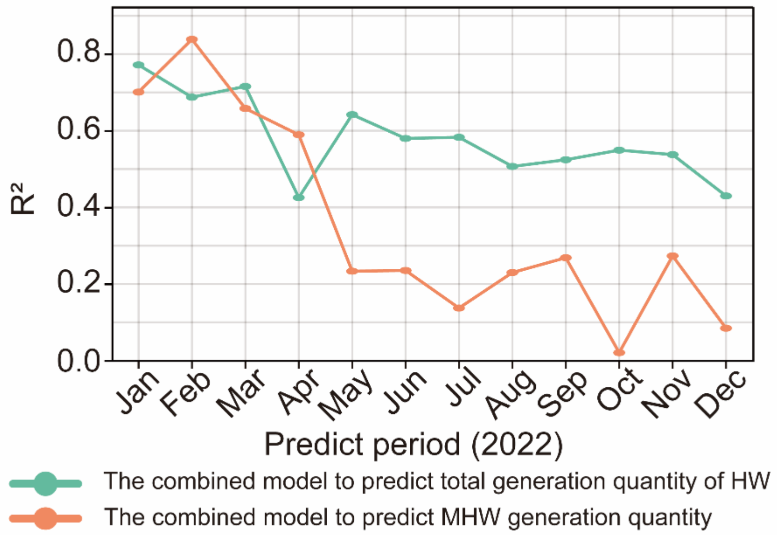

Supplementary Figure 14. The temporal extrapolation of the model during application. The prediction models were trained with the data the Jan. 2020 to Dec. 2021, and were then used to predict the generation quantity of HW in each month in 2022.

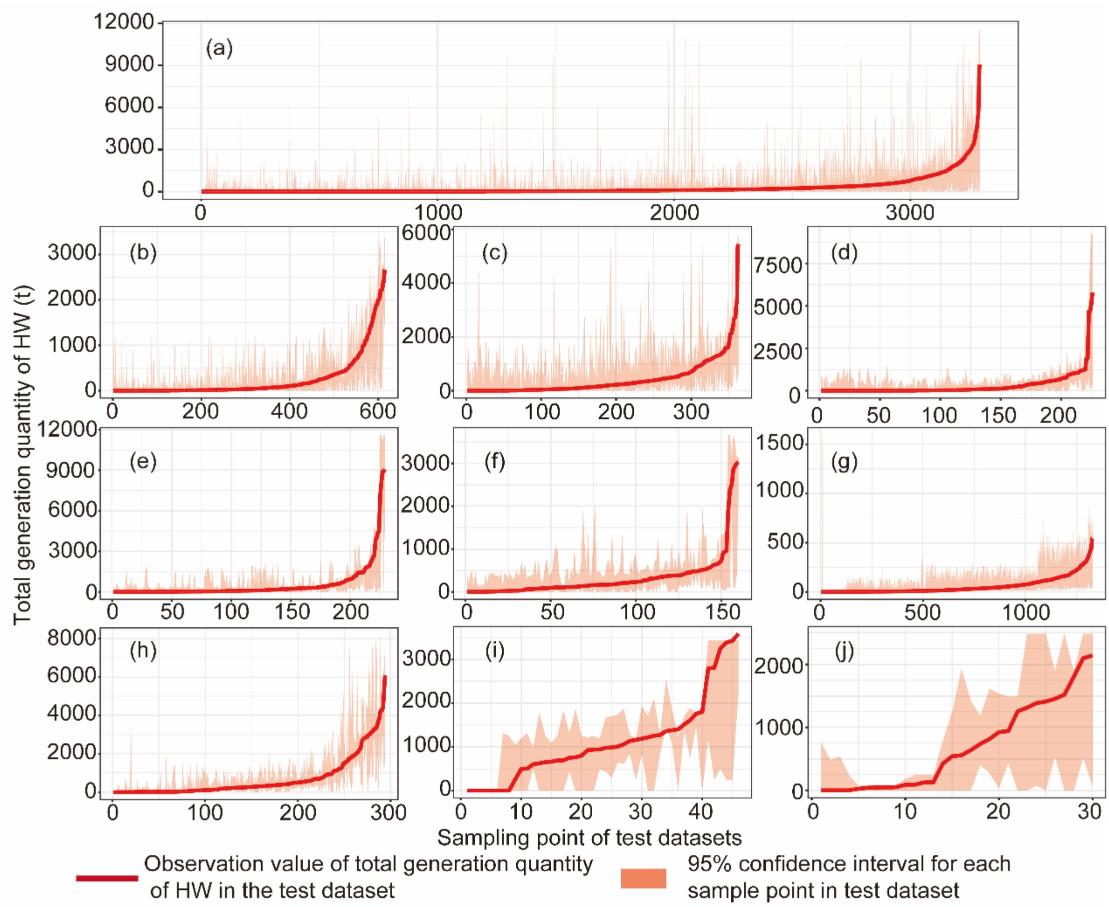

Supplementary Figure 15. Uncertainty assessment of models to predict the total generation quantity of HW. The red curves represent the true value of hazardous waste generation from the test set, ranked from smallest to largest. The shading around the curve indicates the 95% confidence interval of prediction for each sample point in the test set. (a) refers to 10-industrial sectors combined model, (b)~(j) refer to sector-independent models of organic chemical materials manufacture (OCM), chemical pesticides manufacture (CPM), specialized chemical products manufacture (SCP), steel rolling and processing (SRP), metal wire and rope manufacture (MWR), metal surface treatment (MST), electronic circuits manufacture (ECM), biomass energy generation (BEG), and electricity generation using other sources (EGU), respectively.

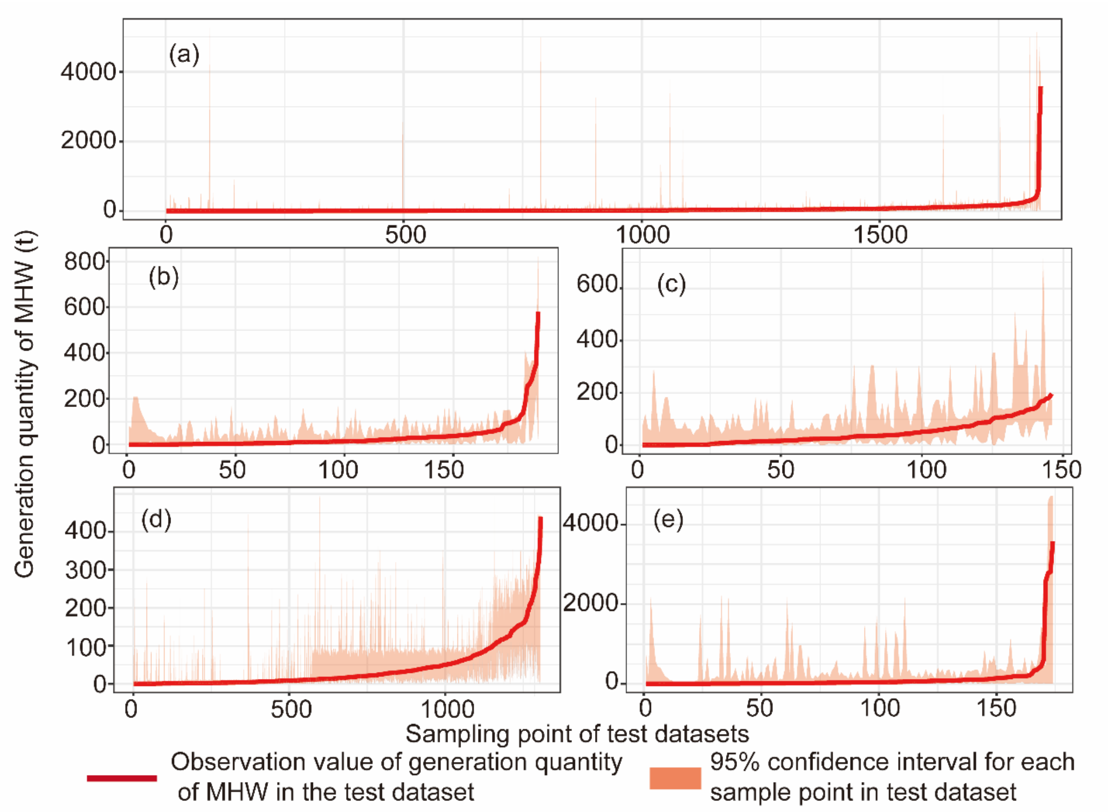

Supplementary Figure 16. Uncertainty assessment of models to predict the generation quantity of MHW. The red curves represent the true value of hazardous waste generation from the test set, ranked from smallest to largest. The shading around the curve indicates the 95% confidence interval of prediction for each sample point in the test set. (a) refers to 10 industrial sectors combined model. (b)~(e) refer to sector-independent models of steel rolling and processing (SRP), metal wire and rope manufacture (MWR), metal surface treatment (MST), and electronic circuits manufacture (ECM).

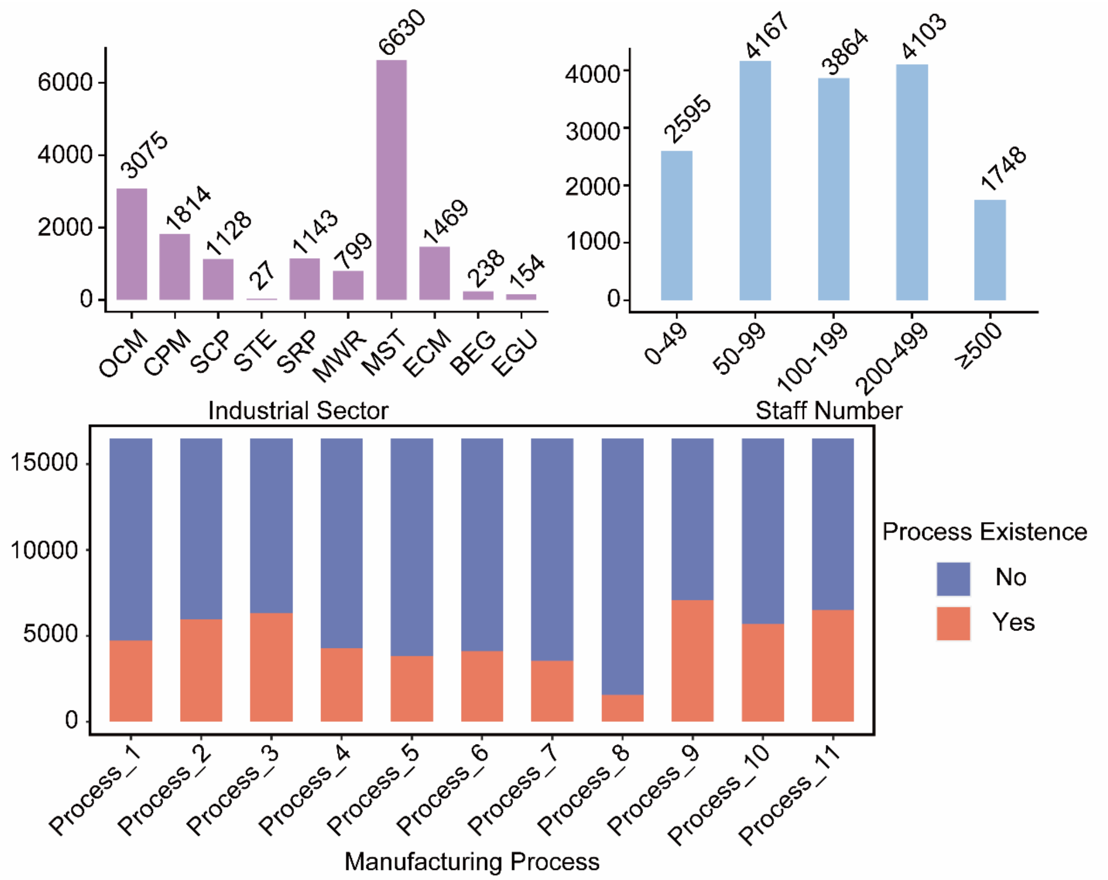

Supplementary Figure 17. The sample size for (a) each industrial sector, (b) the group of each firm-scale, and (c) the group with or without the manufacturing process 1-11 in the cleaned whole dataset (16,477).

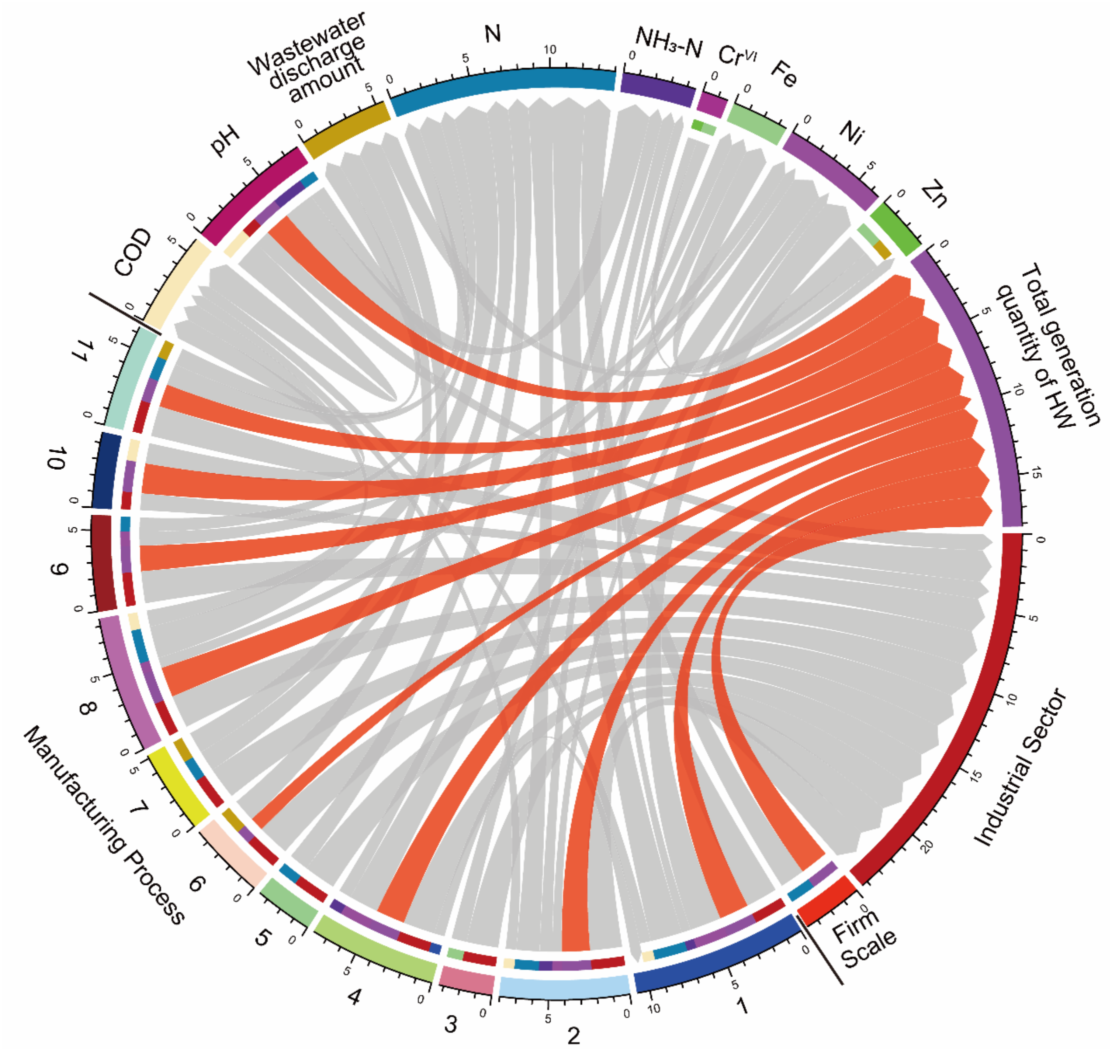

Supplementary Figure 18. The causal relationship between each input feature and the total generation quantity of HW. It is calculated based on the 10 industrial sectors combined data set. The figure shows each feature represented by an edge on the outermost circle, with causal relationships between features connected by indicator strips. The beginning and arrow end of each strip indicate the cause and effect, respectively. The red strips correspond to the feature sets (the parents of total generation quantity of HW) that are highly correlated with the target variable (total generation quantity of HW) as obtained through the Markov blanket search algorithm.

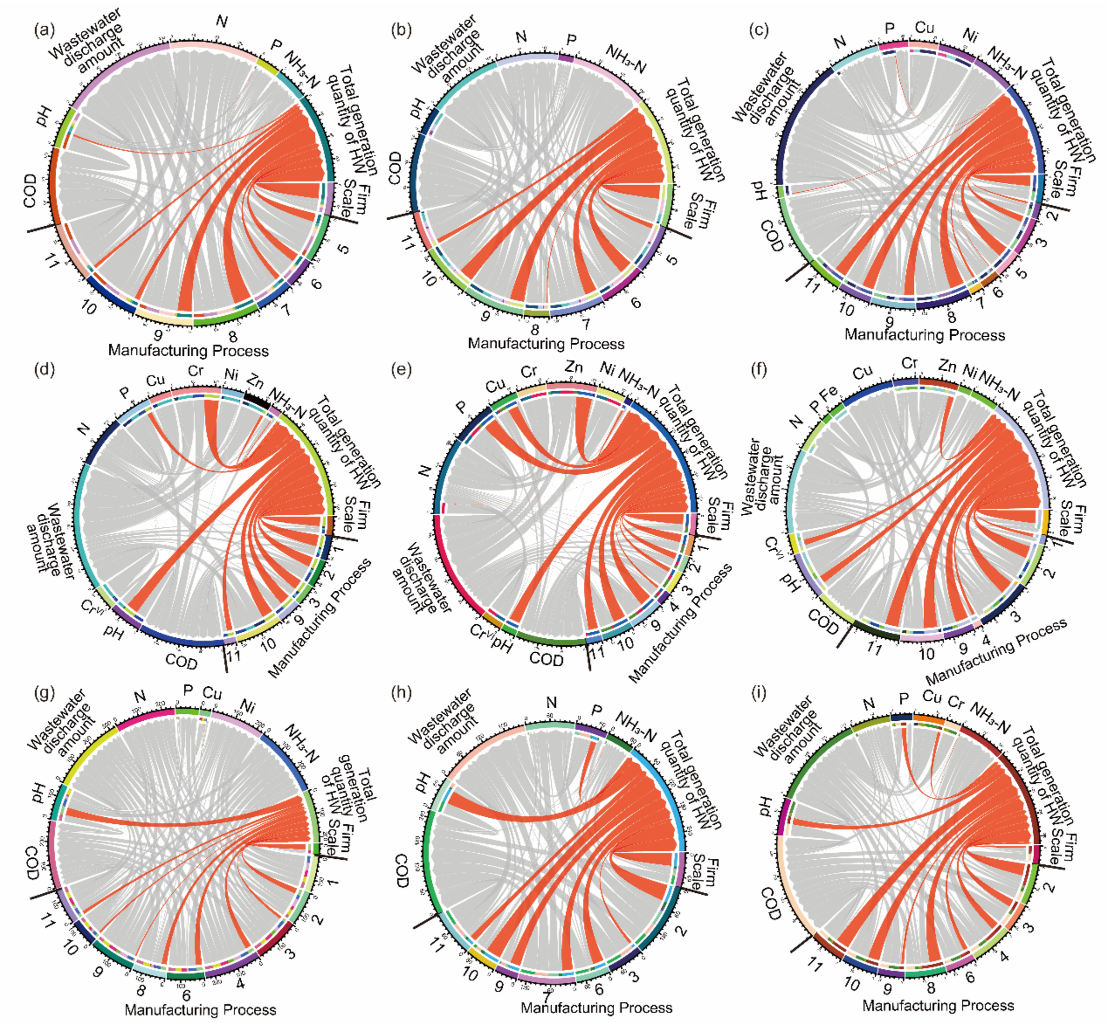

Supplementary Figure 19. The causal relationship between each input feature and the total generation quantity of HW. It is calculated based on each sector data set. (a)~(i) are industrial sectors of organic chemical materials manufacture (OCM), chemical pesticides manufacture (CPM), specialized chemical products manufacture (SCP), steel rolling and processing (SRP), metal wire and rope manufacture (MWR), metal surface treatment (MST), electronic circuits manufacture (ECM), biomass energy generation (BEG), and electricity generation using other sources (EGU), respectively. The figure shows each feature represented by an edge on the outermost circle, with causal relationships between features connected by indicator strips. The beginning and arrow end of each strip indicate the cause and effect, respectively. The red strips correspond to the feature sets (the parents of total generation quantity of HW) that are highly correlated with the target variable (total generation quantity of HW) as obtained through the Markov blanket search algorithm.

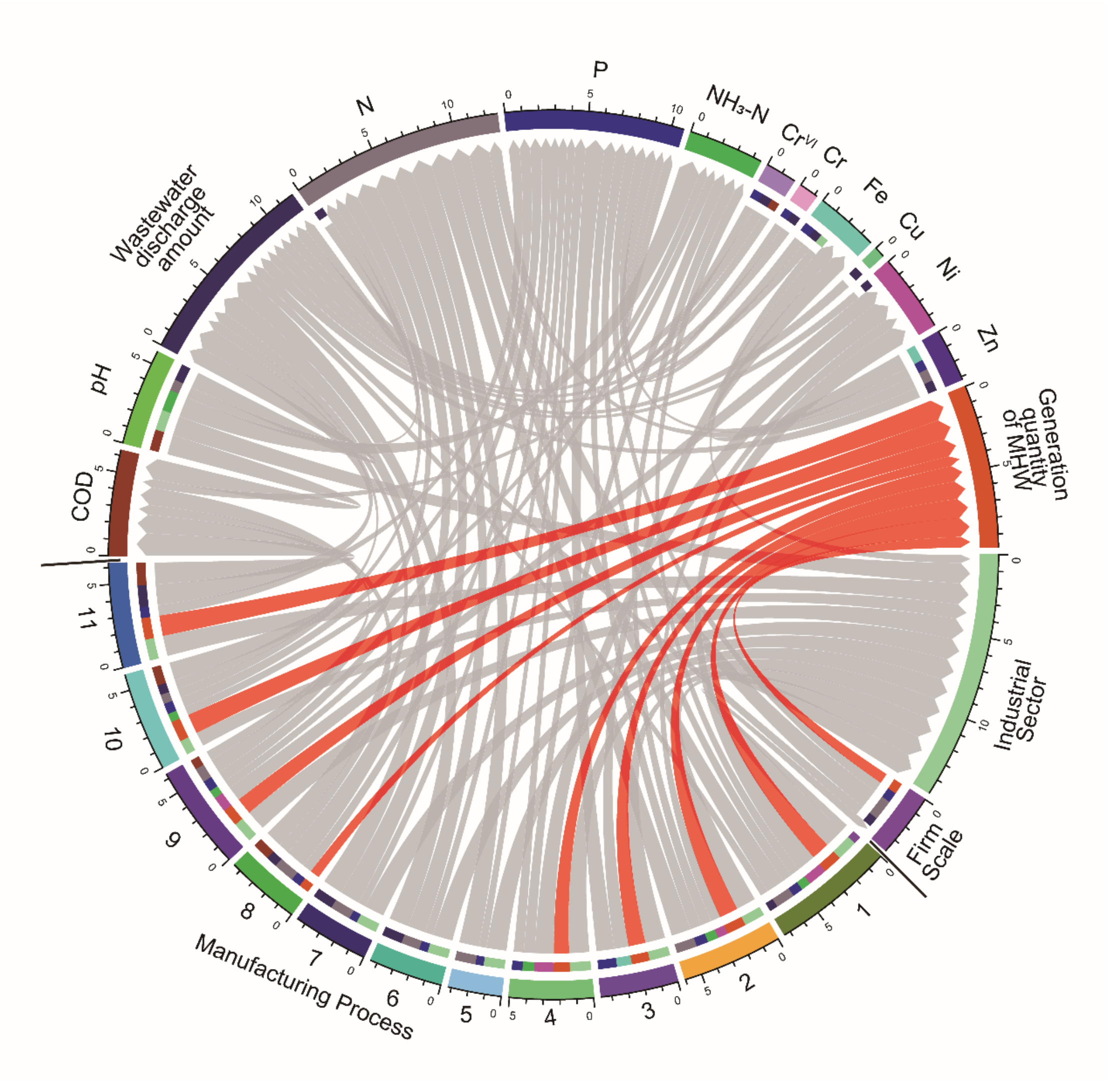

Supplementary Figure 20. The causal relationship between each input feature and the generation quantity of MHW. It is calculated based on the 10 industrial sectors combined data set. The figure shows each feature represented by an edge on the outermost circle, with causal relationships between features connected by indicator strips. The beginning and arrow end of each strip indicate the cause and effect, respectively. The red strips correspond to the feature sets (the parents of generation quantity of MHW) that are highly correlated with the target variable (generation quantity of MHW) as obtained through the Markov blanket search algorithm.

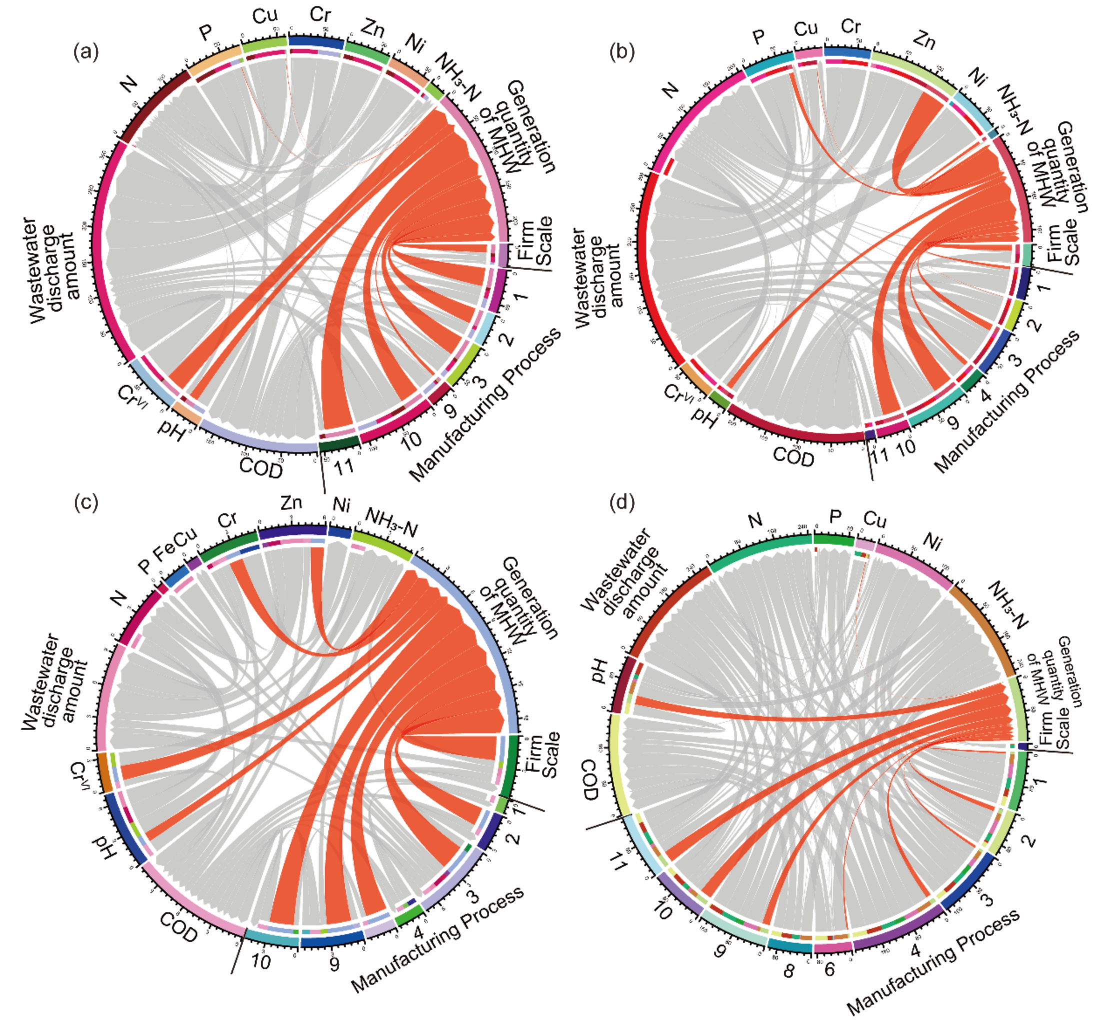

Supplementary Figure 21. The causal relationship between each input feature and the generation quantity of MHW. It is calculated based on each sector data set. (a)~(d) are industrial sectors of steel rolling and processing (SRP), metal wire and rope manufacture (MWR), metal surface treatment (MST), electronic circuits manufacture (ECM). The figure shows each feature represented by an edge on the outermost circle, with causal relationships between features connected by indicator strips. The beginning and arrow end of each strip indicate the cause and effect, respectively. The red strips correspond to the feature sets (the parents of generation quantity of MHW) that are highly correlated with the target variable (total generation quantity of MHW) as obtained through the Markov blanket search algorithm.

Supplementary Table 1. Literature review on relationship between wastewater and hazardous waste generation.

Hazardous waste code	Hazardous waste name	Source of the Waste	Is it related to wastewater emission indicators?	Correlated wastewater emission indicators	Reference/Source	Relationship description
HW01	Medical wastes	Clinical wastes from medical care in hospitals, medical centers and clinics	No			
HW02	Pharmaceutical wastes	Waste from producing original medicine for chemical medicine products	Yes	COD\pH\N\SS\BOD	LaPara et al.[36]	Pharmaceutical wastes primarily originate from the distillation and filtration processes involved in the production of chemical and veterinary medicines, resulting in reaction residues, waste adsorbents, and other by-products. Additionally, the waste liquid produced during these processes can impact wastewater indicators such as COD and pH.
		Waste from producing chemical medicine agents				
		Waste from manufacturing veterinary medicine products			Rana et al.[37]	
		Waste from manufacturing biological, bio-chemical products				
HW03	Obsolete medicine	Waste not from specific sources	No			
HW04	Pesticide wastes	Waste from manufacturing pesticides	Yes	COD\ NH_3-N\P\sulphate	Jia et al.[38]	Pesticide wastes are generated from reaction residues, waste residues, and adsorbents during distillation and filtration processes. Additionally, the wastewater generated from these processes affects the levels of COD, NH_3-N, P, and sulphide in the wastewater.
		Waste not from specific sources				

Hazardous waste code	Hazardous waste name	Source of the Waste	Is it related to wastewater emission indicators?	Correlated wastewater emission indicators	Reference/Source	Relationship description
HW05	Wastes containing wood preserving chemicals	Waste from processing industrial wood and wood chips	Yes	Cu\Cr\As	Kim et al.[39]	An important source of wastes containing wood preserving chemicals are the wastewater treatment sludge produced during the production of wood preservatives and the use of preservatives for wood. During this process, the resulting wastewater contains phenols, heavy metals and other substances that affect the content of COD, Cu, Cr, As and other indicators of wastewater.
		Waste from manufacturing chemical products for special purposes				
		Waste not from specific sources				
HW06	Organic solvent wastes	Waste not from specific sources	Yes	COD\BOD\TOC\SS\pH\P\N\Cr\Cu\Fe\Ni\Zn\Pb\Hg	Güneş et al.[40]	Organic solvent wastes are solvents and wastes containing solvents that are discarded after being used for cleaning, extraction, or as reaction media. These processes generate wastewater containing organic matter, which affects the concentration of chemical oxygen demand, ammonia nitrogen, total organic carbon, and other indicators in the wastewater.
HW07	Wastes from heat treatment containing cyanide	Waste from metal surface treatment and heat treatment	Yes	Cr\Cu\Fe\Ni\Zn\Pb\cyanide\pH	Manyuchi et al.[41]	Wastes from heat treatment containing cyanide are mainly used in the metal heat treatment process of quenching and in the cleaning residue of cyanide-containing equipment. This process produces wastewater that contains cyanide

Hazardous waste code	Hazardous waste name	Source of the Waste	Is it related to wastewater emission indicators?	Correlated wastewater emission indicators	Reference/Source	Relationship description
						and heavy metal ions, which can affect the pH of the wastewater as well as the cyanide and metal ion content.
HW08	Waste mineral oils and waste containing mineral oils	Waste from exploitation of crude oil	Yes	SS\COD\pH\NH3-N\ Cr\TOC\Cu\Fe\Ni\Zn	Costa et al.[42]	Waste mineral oils and waste containing mineral oils are primarily derived from residues and oily sludge generated during oil and gas extraction, storage, transport, and refining. It also includes waste lubricating oil and hydraulic oil generated during the production, maintenance, and cleaning of mechanical devices. During industrial processes, various types of wastewater are generated, such as drilling wastewater, cleaning wastewater, and oil refining wastewater. These wastewaters contain petroleum, organic oils, mineral oils, and other substances that can affect the levels of COD, TOC, pH, SS, and other indicators in the wastewater.
		Waste from exploitation of natural gas			Rezakazemi et al.[43]	
		Waste from manufacturing refined oil products			Cailleaud et al.[44]	
		Waste generated from the manufacturing of electronic components and specialized materials			Park et al.[45]	
		Waste from the rubber products industry				
		Waste not from specific sources				
HW09	Oil/water, hydrocarbon/water mixture or emulsified liquid	Waste not from specific sources	Yes	COD\ BOD\TOC\SS\pH	Ma et al.[46]	The generation of oil/water, hydrocarbon/water mixture or emulsified liquid are mainly due to the use of emulsions in the maintenance of hydraulic presses and metal cutting processes. Wastewater is also produced

Hazardous waste code	Hazardous waste name	Source of the Waste	Is it related to wastewater emission indicators?	Correlated wastewater emission indicators	Reference/Source	Relationship description
						during these processes, and some of the emulsions enter the wastewater, affecting the concentration of pollutants such as COD, BOD, SS, pH value, and TOC.
HW10	Wastes containing PCBs and PBBs	Waste not from specific sources	Yes	COD\pH	Abbasi et al.[47]	Wastes containing PCBs and PBBs primarily come from electrical equipment that contains PCBs, including the cleaning and disposal of such equipment. The cleaning solution generated during the cleaning process of PCB-containing equipment affects wastewater indicators, such as COD and pH.
HW11	Residues of rectifying and distillation	Waste from manufacturing refined oil products	Yes	pH\COD\N\NH_3-N\Fe\Cr\Cu\Zn\TOC\sulphate	Elmobarak et al.[48]	Residues of rectifying and distillation are primarily derived from the refining and processing of petroleum, coal, and natural gas, as well as from the distillation and fractionation processes used in the production of chemical raw materials. These processes generate wastewater containing tar, organic matter, and other substances that affect the pH value, COD, N, cyanide, and heavy metal concentrations.
		Waste from coal processing		pH\NH_3-N\SS\cyanide\COD\Fe\Ni\Zn\Cu\Cr	Maiti er al.[49]	
		Waste from gas production and supply industry				
		Waste from manufacturing of basic chemical raw materials		COD\pH	Yang et al.[50]	
		Waste from the manufacturing of graphite and other non-metallic mineral products				

Hazardous waste code	Hazardous waste name	Source of the Waste	Is it related to wastewater emission indicators?	Correlated wastewater emission indicators	Reference/Source	Relationship description
		Waste from environmental management industry		COD	Vineetha et al.[51]	
		Waste not from specific sources				
HW12	Waste dyes and paints	Waste from manufacturing paints, printing ink, dyes and other relevant products	Yes	COD\pH\P\N\SS\BOD\ sulphate\Ni	Aniyikaiye et al.[52]	Waste dyes and paints mainly originate from the production process of pigments, which results in the generation of wastewater containing organic matter and heavy metal ions. This affects the wastewater's COD, pH, N, SS, and metal ion levels. The wastewater treatment sludge produced during this process also contributes to the waste dyes and paints.
		Waste not from specific sources		COD\pH\BOD\SS	Nair et al.[53]	
HW13	Organic resins wastes	Waste from manufacturing synthetic materials	Yes	pH\COD\N\NH_3-N	Cao et al.[54]	Organic resins wastes are primarily generated from the mother liquor waste liquid produced during the synthesis, esterification, condensation, and other processes involved in the production of resins, latex, and plasticizers. Additionally, residues are generated from distillation, separation, refining, and other processes, which synchronously generate wastewater containing various organic substances. This wastewater affects the pH, COD, N, and NH_3-N of the wastewater.
		Waste not from specific sources				

Hazardous waste code	Hazardous waste name	Source of the Waste	Is it related to wastewater emission indicators?	Correlated wastewater emission indicators	Reference/Source	Relationship description
HW14	Wastes containing unknown chemicals	Waste not from specific sources	No			
HW15	Explosive wastes	Waste from manufacturing dynamite, pyrotechnic and fireworks products	Yes	pH\COD\N\SS\sulphate	Bhanot et al.[55]	Explosive wastes are primarily produced from sludge generated during the wastewater treatment process in the manufacture of explosives. This process generates wastewater that contains organic matter, which affects the COD, pH, and N of the wastewater.
HW16	Photographic chemical wastes	Waste from manufacturing chemical products for special purposes	Yes	COD\pH\N\SS	Papadopoulos et al.[56]	Photographic chemical wastes comprise developers, positive and negative films, image paper, and their production residues. Additionally, sludge from the wastewater treatment process in the production of these materials contributes to this waste, with synchronous discharges of wastewater containing organic compounds and metals. The presence of organic compounds and heavy metals in wastewater affects COD, pH, N, and Cu.
		Waste from printing industry				
		Waste generated from the manufacturing of electronic components and specialized materials		Cu\pH	Adamović et al.[57]	
		Waste from film industry				
		Waste from photography and photo-enlarging services		COD	Stalikas et al.[58]	
		Waste not from specific sources				

Hazardous waste code	Hazardous waste name	Source of the Waste	Is it related to wastewater emission indicators?	Correlated wastewater emission indicators	Reference/Source	Relationship description
HW17	Metal surface treatment waste	Wastes from metal surface treatment and heat treatment	Yes	Cr\Cu\Fe\Ni\Zn\pH	Viguri et al.[59]	Metal surface treatment wastes are generated from wastewater treatment sludge from metal surface treatment processes such as electroplating, descaling, degreasing and pickling. Synchronized generation of wastewater containing heavy metals affects the pH and heavy metal content of the water.
HW18	Residues of incinerating disposal	Waste from environmental treatment	Yes	Cu\Zn\Cr\Cd\Hg\Pb\As\COD\pH	Xiong et al.[60]	Residues of incinerating disposal primarily consist of domestic waste and hazardous waste incineration residue, fly ash, and wastewater treatment sludge. During the wastewater treatment process, wastewater containing heavy metals is generated concurrently.
				Fe\Zn\Cr\Cu\Ni\pH	Astrup et al.[61]	
HW19	Metal carbonyl compound wastes	Waste not from specific sources	Yes	COD\sulphate\Fe\Co\Ni	Manasfi et al.[62] Cesari et al.[63]	Metal carbonyl compound wastes are primarily derived from the waste generated during the production and application of metal carbonyl compounds. During the production of metal carbonyl compounds, wastewater containing metal ions is discharged, which affects the pH and metal ion content of the wastewater.
HW20	Beryllium wastes	Waste from the manufacturing of basic chemical raw materials	Yes	pH\Fe\Cu\sulphate	Zhong et al.[64]	Beryllium wastes are produced during the manufacture of beryllium and its compounds. These wastes

Hazardous waste code	Hazardous waste name	Source of the Waste	Is it related to wastewater emission indicators?	Correlated wastewater emission indicators	Reference/Source	Relationship description
						consist of furnace slag, dust collected by dust collectors, and wastewater treatment sludge. Additionally, metal ion-containing wastewater is discharged during the wastewater treatment process, which affects the pH and other indicators of the wastewater.
HW21	Chromium wastes	Waste from fur dressing and processing	Yes	COD\Cr\$NH_3$-N\SS\pH\Cr\$Cr^{VI}$	Sawalha et al.[65]	Chromium wastes are produced during fur tanning, metal smelting and processing, and chromium salt production. These wastes include residues and treatment sludge generated during processing, reaction and wastewater treatment. The wastewater discharged during these processes contains chromium ions and other pollutants that affect COD, pH, and metal ion content.
		Waste from the manufacturing of basic chemical raw materials				
		Waste from smelting ferro-ally		COD\BOD\pH\SS\cyanide\Cr\Cu\Ni\Zn	Abdel Wahaab et al.[66]	
		Waste from metal surface treatment and heat treatment				
		Waste generated from the manufacturing of electronic components and specialized materials				
HW22	Copper wastes	Waste from producing glass	Yes	Cu\Ni\Fe\Cr\Zn	John et al.[67]	Copper wastes originate primarily from waste tank liquids and wastewater treatment sludge generated during processes such as copper plating, copper plate etching, and copper oxide treatment. These processes produce wastewater containing
		Waste generated from the manufacturing of electronic components and specialized materials				

Hazardous waste code	Hazardous waste name	Source of the Waste	Is it related to wastewater emission indicators?	Correlated wastewater emission indicators	Reference/Source	Relationship description
						metal ions, including copper, which affects the metal content of the wastewater.
HW23	Zinc wastes	Waste from metal surface treatment and heat treatment<br>Waste from producing batteries<br>Waste from steelmaking<br>Waste not from specific sources	Yes	Zn\Cr\CrVI\Pb\Cu\Ni\As\Cd\Hg	Barakat[68]	The zinc wastes originate from the production of batteries, metal galvanizing, steelmaking, and the use of zinc dust in the process of precipitating precious metals, resulting in the generation of zinc-containing sludge and residues. These processes simultaneously produce wastewater containing zinc and other metal ions, thereby impacting the overall metal ion content.
HW24	Arsenic wastes	Waste from the manufacturing of basic chemical raw materials	Yes	pH\Cr\Cu\Zn\As\Cd\Pb	Yao et al.[69]	Arsenic wastes stem from the acid sludge generated during the utilization of dilute acid to cleanse flue gases in the sulfuric iron ore acid production process. This process produces wastewater containing both acid and metal ions, thereby influencing pH levels and the concentrations of other metal ions.
HW25	Selenium wastes	Waste from the manufacturing of basic chemical raw materials	Yes	Se\Cr\Zn\Ni\Cd\Hg	Staicu et al.[70]	Selenium wastes arise from the slag produced in the manufacturing of selenium and its compounds, along with dust collected by dust collectors and sludge from wastewater treatment. The

Hazardous waste code	Hazardous waste name	Source of the Waste	Is it related to wastewater emission indicators?	Correlated wastewater emission indicators	Reference/Source	Relationship description
						wastewater treatment process results in the discharge of wastewater containing selenium and other metals, thereby impacting both pH and the concentrations of metal ions.
HW26	Cadmium wastes	Waste from producing batteries	Yes	Cd\Ni\Fe	Volynskii et al.[71]	Cadmium wastes originate from the waste sludge and wastewater treatment sludge produced in the manufacturing of nickel-cadmium batteries. The wastewater treatment process concurrently releases wastewater containing cadmium and other metal ions, consequently influencing the metal ion composition of the discharged wastewater.
HW27	Antimony wastes	Waste from the manufacturing of basic chemical raw materials	Yes	Sb\As\Fe\Cu\Cr\Zn\Ni\Pb\Hg\Co	Guo et al.[72]	Antimony wastes predominantly arise from the slag generated in the production processes of antimony smelting and antimony oxidation, in addition to dust collected by dust removal devices. The smelting and oxidation of antimony give rise to various wastewater streams, including slag flushing and flue gas cleaning. These wastewater streams carry metal ions, thereby influencing the metal ion content in the overall wastewater.

Hazardous waste code	Hazardous waste name	Source of the Waste	Is it related to wastewater emission indicators?	Correlated wastewater emission indicators	Reference/Source	Relationship description
HW28	Tellurium wastes	Waste from the manufacturing of basic chemical raw materials	Yes	pH	Yao et al.[73]	Tellurium wastes originate from the slag produced in the manufacturing of tellurium and its compounds, along with dust collected by dust collectors and sludge from wastewater treatment. The wastewater treatment process involves the discharge of acidic wastewater, thereby impacting the pH.
HW29	Mercury wastes	Waste from exploitation of natural crude oil and natural gas	Yes	sulphate\Hg\Fe\As	Lothongkum et al.[74]	Mercury wastes are derived from various sources, including the mercury removal process in metal smelting, the production of mercury through electrolysis cell methods, and processes involving vinyl chloride and other halogen-containing chemicals, such as those used in the calcium carbide acetylene method. Additionally, residues from mercury-containing equipment manufacturing, battery production, and wastewater treatment sludge contribute to the presence of mercury wastes. In the mercury electrolysis, calcium carbide acetylene production, battery manufacturing, and wastewater treatment processes, there is concurrent discharge of
		Waste from common non-ferrous metals ore mining		Hg\As\Cd\Cr\Cu\Zn	Ning et al.[75]	
		Waste from precious metal smelting				
		Waste from printing				
		Waste from the manufacturing of basic chemical raw materials				
		Waste from manufacturing synthetic materials				
		Waste from common nonferrous metals smelting				
		Waste from producing batteries		Hg\Pb\Cd\Fe\Zn	Bernardes et al.[76]	

Hazardous waste code	Hazardous waste name	Source of the Waste	Is it related to wastewater emission indicators?	Correlated wastewater emission indicators	Reference/Source	Relationship description
		Waste from manufacturing lighting apparatus				wastewater containing metal ions, including mercury, thereby influencing the overall metal content of the discharged wastewater.
		Waste from manufacturing general instruments and meters				
		Waste not from specific sources				
HW30	Thallium wastes	Waste from the manufacturing of basic chemical raw materials	Yes	Fe\Cu\Zn	Zhang et al.[77]	Thallium wastes originate from the slag produced in the manufacturing of thallium and its compounds, as well as dust collected by dust collectors and sludge from wastewater treatment. Simultaneously, the wastewater treatment process results in the discharge of wastewater containing metal ions, thereby influencing the metal ion content.
HW31	Lead wastes	Waste from manufacturing glass and glass products	Yes	Pb\Sb\As\Fe\Cu\Zn	Ma et al.[78]	Lead wastes primarily stem from waste liquids produced during the electroplating of lead-tin alloys in the manufacturing of circuit boards, as well as the wastewater treatment sludge generated in the production of lead storage batteries. This production process yields both plating cleaning wastewater and battery production
		Waste from the manufacturing of electronic components and electronic special materials				
		Waste from manufacturing of batteries				

Hazardous waste code	Hazardous waste name	Source of the Waste	Is it related to wastewater emission indicators?	Correlated wastewater emission indicators	Reference/Source	Relationship description
		Waste from manufacturing arts and crafts		Pb\Sb\As\Fe\Zn	Kreusch et al.[79]	wastewater, both of which contain lead and other metal ions.
		Waste not from specific sources				
HW32	Inorganic fluoride wastes	Waste not from specific sources	Yes	pH\Cu\sulphate	Yu et al.[80]	Inorganic fluoride wastes result from the utilization of hydrofluoric acid in the etching process of electronic and electrical equipment, leading to the generation of waste liquid. The etching process further produces equipment cleaning wastewater, containing COD, sulphate, and other contaminants.
HW33	Inorganic cyanide wastes	Waste from mining and metallurgy of precious metals	Yes	cyanide\pH\Cu\Zn\Fe	Kuyucak et al.[81]	Inorganic cyanide wastes primarily originate from the application of cyanide in the ore dressing process for precious metals and its use in metal treatment processes such as dipping and polishing. These processes give rise to both ore dressing wastewater and metal surface processing wastewater, which encompass a range of pollutants, including cyanide and metal ions.
		Waste from surface treatment and heat treatment		cyanide\sulphate\Cu\Zn\Ni\Co	Kuyucak et al.[81]	
		Waste not from specific sources				
HW34	Waste acids	Waste from manufacturing of refined oil products	Yes	pH	Shibasaki-Kitakawa et al.[82]	Waste acids predominantly encompass the byproducts of petroleum refining, specifically

Hazardous waste code	Hazardous waste name	Source of the Waste	Is it related to wastewater emission indicators?	Correlated wastewater emission indicators	Reference/Source	Relationship description
		Waste from the manufacturing of paints, inks, pigments and similar products		pH\sulphate\Fe\V	Qiu et al.[83]	waste acids and acid sludge. Additionally, waste acids and acid sludge are generated in the manufacturing processes of chemical raw materials. Furthermore, waste acids are produced in metal processing and circuit manufacturing processes such as pickling, acid etching, and electrolysis. In these processes, wastewater is discharged simultaneously, containing acid, metal ions, and various pollutants. This discharge has an impact on the pH, metal ion content, and other indicators of the wastewater.
		Waste from the manufacturing of basic chemical raw materials		pH\sulphate\Fe\Cr	Wei et al.[84]	
		Waste from steel press forging		pH\cyanide\Fe\Cr\Cr^{VI}\Ni	Agrawal et al.[85]	
		Waste from metal surface treatment and heat treatment		pH\Zn\Fe	Stocks et al.[86]	
		Waste from manufacturing electronic components		pH\N\P\Fe	Kim et al.[87]	
		Waste not from specific sources				
HW35	Waste alkali	Waste from manufacturing refined oil products	Yes	pH\sulphate\volatile phenol\TOC\COD\BOD	Hariz et al.[88]	Waste alkali primarily originate from the byproducts of various industrial processes, including waste lye and lye residue produced in petroleum refining, as well as waste lye and residues generated during processes involving calcium hydroxide, ammonia, etc. Additionally, waste lye is a byproduct of fur processing, the alkaline pulping process in papermaking, and various applications such as plating and cleaning. These processes result in
		Waste from the manufacturing of basic chemical raw materials				
		fur dressing and processing		pH	Weiet al.[89]	
		Waste from paper pulp making				
		Waste not from specific sources		pH\COD\TOC\BOD	Götz et al.[90]	

Hazardous waste code	Hazardous waste name	Source of the Waste	Is it related to wastewater emission indicators?	Correlated wastewater emission indicators	Reference/Source	Relationship description
						the simultaneous generation of diverse wastewater streams, including petroleum refining wastewater, chemical production wastewater, fur processing wastewater, plating wastewater, and others. These wastewaters encompass a range of pollutant indicators, including pH, COD, BOD, TOC, and etc.
HW36	Asbestos wastes	Waste from mining and smelting asbestos<br>Waste from the manufacturing of basic chemical raw materials	Yes	Cr\Ni	Kumar et al.[91]	Asbestos wastes are derived from various sources, including asbestos mining, gypsum cement production, and the manufacturing processes of asbestos-containing mechanical parts. These processes concurrently generate mining wastewater, gypsum cement production wastewater, and wastewater from cleaning mechanical parts. These wastewaters contain asbestos, sulphate, metal ions, and other pollutants, influencing the content of metal ions and sulphate in the overall wastewater.
		Waste from manufacturing cement and gypsum products<br>Waste from manufacturing fire-proof materials<br>Waste from automobile making		Fe\As	Spasiano et al.[92]	
		Waste from manufacturing ships and floating installations<br>Waste not from specific sources		Fe\sulphate	Colangelo et al.[93]	

Hazardous waste code	Hazardous waste name	Source of the Waste	Is it related to wastewater emission indicators?	Correlated wastewater emission indicators	Reference/Source	Relationship description
HW37	Organic phosphorus wastes	Waste from the manufacturing of basic chemical raw materials	Yes	P	Cristaleet al.[94]	Organic phosphorus wastes primarily originate from the production and formulation processes of organophosphorus compounds, excluding pesticides. These wastes include reaction residues, waste adsorbents, and sludge from wastewater treatment. Simultaneously, the production of organophosphorus compounds results in the discharge of phosphorus-containing wastewater, impacting the phosphorus content of the wastewater.
		Waste not from specific sources				
HW38	Organic cyanide wastes	Waste from the manufacturing of basic chemical raw materials	Yes	cyanide\COD\NH_3-N\pH	Zheng et al.[95]	Organic cyanide wastes primarily result from distillation residues, spent mother liquor, and reaction residues produced during the manufacturing of acrylonitrile and other organic cyanides. In the course of this production, distillation wastewater and chemical reaction wastewater are generated, influencing the concentrations of various pollutants in the wastewater, including cyanide, COD, NH_3-N, and pH.
				COD\N\TOC	Dai et al.[96]	
				cyanide\COD\NH_3-N	Han et al.[97]	
				pH\COD	Huang et al.[98]	
HW39	Phenols wastes	Waste from the manufacturing of basic chemical raw materials	Yes	volatile phenol\COD	Said et al.[99]	Phenols wastes and ethers wastes primarily arise from waste mother liquor, reaction residues, distillation
					Hussain et al.[100]	

Hazardous waste code	Hazardous waste name	Source of the Waste	Is it related to wastewater emission indicators?	Correlated wastewater emission indicators	Reference/Source	Relationship description
HW40	Ethers wastes	Waste from the manufacturing of basic chemical raw materials	Yes	COD\petroleum hydrocarbon pollutants\NH_3-N\N\SS\pH	Liang et al.[101]	residues, and sludge produced in the manufacturing of phenolic and ether compounds. These production processes are accompanied by the generation of distillation wastewater and chemical reaction wastewater, impacting the levels of pollutants such as COD, volatile phenols, and others in the wastewater.
HW45	Organ halogen compound wastes	Waste from the manufacturing of basic chemical raw materials	Yes	COD	Gomez-Rico et al.[102]	Organ halogen compound wastes primarily originate from reaction residues, waste adsorbents, and sludge resulting from the production of organic halides. Concurrently, the production process generates chemical reaction wastewater, containing organic halides and impacting the COD of the wastewater.
HW46	Nickel compound wastes	Waste from the manufacturing of basic chemical raw materials	Yes	Ni\Cr^{VI}\Zn	Costa et al.[103]	Nickel compound wastes result from reaction residues in the production of nickel compounds, sludge generated in the production of nickel-metal hydride batteries, and wastewater treatment sludge. These processes encompass the discharge of wastewater from chemical reactions, production processes, and wastewater containing metal ions, including Ni,
		Waste from manufacturing batteries			Kumar et al.[104]	
		Waste not from specific sources				

Hazardous waste code	Hazardous waste name	Source of the Waste	Is it related to wastewater emission indicators?	Correlated wastewater emission indicators	Reference/Source	Relationship description
						thereby influencing the metal ion content in the discharged wastewater.
HW47	Barium compound wastes	Waste from the manufacturing of basic chemical raw materials	Yes	pH\Pb\Zn\Cd	Gu et al.[105]	Barium compound wastes primarily stem from reaction residues produced in the manufacturing of barium compounds, as well as wastewater treatment sludge. Additionally, barium-containing salt bath sludge is generated during metal heat treatment processes. These processes entail the discharge of chemical reaction wastewater and metal processing wastewater, consequently influencing the concentration of metal ions in the wastewater.
HW48	Non-ferrous metal smelting wastes	Waste from smelting common non-ferrous metals	Yes	Fe\Cu\Cr\Zn\Ni\As\Sb\Cd\Co\Hg\Pb	Guo et al.[72]	Non-ferrous metal smelting wastes primarily originate from non-ferrous metal smelting activities, including smelting slag, flue gas purification dust, and acid cake, as well as metal leaching residues and electrolysis residues. These processes concurrently generate smelting cooling water, flue gas purification wastewater, metal leaching, and electrolysis wastewater, containing various metal ions. The discharge of such wastewater has an impact on the
		Waste from the smelting of rare earth metals			Kumar et al.[104]	
					Yu et al.[106]	

Hazardous waste code	Hazardous waste name	Source of the Waste	Is it related to wastewater emission indicators?	Correlated wastewater emission indicators	Reference/Source	Relationship description
						pH and metal ion content of the overall wastewater.
HW49	Other wastes	Graphite and other non-metallic mineral products manufacturing industry	Yes	The pollution factors that produce wastewater are typically associated with specific industrial processes.		
		Waste from environmental management industry				
		Waste not from specific sources				
HW50	Catalyst wastes	Waste from the manufacturing of refined petroleum products	Yes	The pollution factors that produce wastewater are typically associated with specific industrial processes.	-	
		Waste from the manufacturing of basic chemical raw materials				
		Waste from the manufacturing of pesticides				
		Waste from the manufacturing of chemical apis				
		Waste from the manufacturing of veterinary drugs				
		Waste from the manufacturing of biological pharmaceutical products				

Hazardous waste code	Hazardous waste name	Source of the Waste	Is it related to wastewater emission indicators?	Correlated wastewater emission indicators	Reference/Source	Relationship description
		Waste from the environmental treatment industry				
		Waste not from specific sources				

Note: The hazardous waste category is derived from Chinese National List of Hazardous Wastes.

Supplementary Table 2. Performance of the combined model to predict the total generation quantity of HW before and after balancing the training dataset.

Model			R^2	RMSE	MAD	MAE	MAPE	MSE	SSE	Input Features
Before balance	Multiple Linear Regression		0.22	592.42	229.42	288.96	7.26	3.5E+05	1.2E+09	process_1, process_2, process_4, process_6, process_8, process_9, process_10, process_11, industrial sector, firm scale, pH, wastewater discharge amount, Fe, Ni
	Machine Learning	GBDT	0.77	322.61	79.93	151.48	1.00	1.0E+05	3.4E+08	process_1, process_2, process_4, process_6, process_8, process_9, process_10, process_11, industrial sector, firm scale, pH, wastewater discharge amount, Fe
		XGB	0.70	370.74	147.52	206.97	1.29	1.4E+05	4.5E+08	process_1, process_2, process_4, process_6, process_8, process_9, process_10, process_11, industrial sector, firm scale, pH, wastewater discharge amount, Ni
		SVM	0.80	299.49	89.74	133.84	0.94	9.0E+04	3.0E+08	process_1, process_2, process_4, process_6, process_8, process_9, process_10, process_11, industrial sector, firm scale, pH, wastewater discharge amount, Ni, Fe
		KNN	0.78	352.71	40.87	129.17	0.53	1.2E+05	1.6E+09	process_1, process_2, process_4, process_6, process_8, process_9, process_10, process_11, industrial sector, firm scale, pH, wastewater discharge amount, Ni
		RF	0.80	270.35	70.08	127.04	0.58	7.3E+04	2.4E+08	process_1, process_2, process_4, process_6, process_8, process_9, process_10, process_11, industrial sector, firm scale, pH, COD, wastewater discharge amount, Fe, Ni
	Deep Learning	MLP	0.67	411.97	58.14	140.74	1.37	1.7E+05	5.6E+08	process_1, process_2, process_4, process_6, process_8, process_9,

Model			R^2	RMSE	MAD	MAE	MAPE	MSE	SSE	Input Features
										process_10, process_11, industrial sector, firm scale, pH, COD, wastewater discharge amount, pH, Fe, Ni
		Ensemble MLP	0.67	411.59	56.79	140.22	2.24	1.7E+05	5.6E+08	process_1, process_2, process_4, process_6, process_8, process_9, process_10, process_11, industrial sector, firm scale, pH, COD, wastewater discharge amount, pH, Fe, Ni
		TNN	0.81	307.85	138.02	175.74	1.88	9.5E+04	3.1E+08	process_1, process_2, process_4, process_6, process_8, process_9, process_10, process_11, industrial sector, firm scale, pH, COD, pH, wastewater discharge amount, Fe, Ni
After balance	Multiple Linear Regression		0.21	606.99	335.75	354.22	1.53	3.7E+05	1.2E+09	process_1, process_2, process_4, process_6, process_8, process_9, process_10, process_11, industrial sector, firm scale, pH, wastewater discharge amount, Fe, Ni
	Machine Learning	GBDT	0.81	298.05	94.53	155.21	1.53	8.9E+04	2.9E+08	process_1, process_2, process_4, process_6, process_8, process_9, process_10, process_11, industrial sector, firm scale, pH, wastewater discharge amount, Fe
		XGB	0.81	296.17	80.51	148.55	13.18	8.8E+04	2.9E+08	process_1, process_2, process_4, process_6, process_8, process_9, process_10, process_11, industrial sector, firm scale, pH, wastewater discharge amount, Fe
		SVM	0.81	295.93	122.73	155.61	0.90	8.8E+04	2.9E+08	process_1, process_2, process_4, process_6, process_8, process_9, process_10, process_11, industrial sector, firm scale, pH, wastewater discharge amount, Fe, Cu
		KNN	0.83	290.70	41.53	120.51	0.77	8.5E+04	2.8E+08	process_1, process_2, process_4,

Model		R^2	RMSE	MAD	MAE	MAPE	MSE	SSE	Input Features
									process_6, process_8, process_9, process_10, process_11, industrial sector, firm scale, pH, wastewater discharge amount, Ni
	RF	0.87	247.40	60.77	122.28	0.56	6.1E+04	2.0E+08	process_1, process_2, process_4, process_6, process_8, process_9, process_10, process_11, industrial sector, firm scale, pH, COD, wastewater discharge amount, Fe, Ni
	MLP	0.86	249.99	61.36	122.13	1.06	6.2E+04	2.1E+08	process_1, process_2, process_4, process_6, process_8, process_9, process_10, process_11, industrial sector, firm scale, pH, COD, wastewater discharge amount, pH, Fe, Ni
Deep Learning	Ensemble MLP	0.87	241.09	55.32	117.96	0.79	5.8E+04	1.9E+08	process_1, process_2, process_4, process_6, process_8, process_9, process_10, process_11, industrial sector, firm scale, pH, COD, wastewater discharge amount, pH, Fe, Ni
	TNN	0.84	295.82	154.73	179.26	1.00	8.8E+04	2.9E+08	process_1, process_2, process_4, process_6, process_8, process_9, process_10, process_11, industrial sector, firm scale, pH, COD, pH, wastewater discharge amount, Fe, Ni

Note that the test dataset used for evaluating the performance of all models is same. Data balance is conducted only on the training dataset.

Supplementary Table 3. Performance of the classification and regression model to predict the generation quantity of MHW after data balance.

Model		Classification				Regression						
		**Accuracy**	**Precision**	**Recall**	**F1**	**R^2**	**RMSE**	**MAD**	**MAE**	**MAPE**	**MSE**	**SSE**
Machine Learning	GBDT	0.95	0.98	0.91	0.94	0.42	130.99	38.54	47.31	0.76	1.7E+04	5.7E+07
	XGB	0.94	0.97	0.88	0.93	0.45	130.34	38.33	47.07	0.85	1.7E+04	5.6E+07
	SVM	0.95	0.96	0.92	0.94	0.30	177.04	61.79	64.88	0.82	3.1E+04	1.0E+08
	KNN	0.95	0.97	0.93	0.95	0.30	181.87	37.93	65.84	0.78	3.3E+04	1.1E+08
	RF	0.96	0.97	0.94	0.95	0.88	62.51	12.52	23.20	0.52	3.9E+03	6.9E+06
Deep Learning	MLP	0.94	0.98	0.90	0.94	0.86	61.15	45.14	35.00	1.12	3.7E+03	6.6E+06
	Ensemble MLP	0.95	0.97	0.91	0.94	0.25	134.94	21.63	30.56	0.92	1.8E+04	3.2E+07
	TNN	0.95	0.97	0.92	0.95	0.83	77.20	72.60	52.95	1.43	6.0E+03	1.1E+07

Supplementary Table 4. Input features of the combined ensemble model to predict the MHW generation quantity.

**Model**		**R^2**	**RMSE**	**MAD**	**MAE**	**MAPE**	**MSE**	**SSE**	**Input Features**
Machine Learning	GBDT	0.48	116.34	4.39	22.73	-	1.4E+04	4.5E+07	process_1, process_2, process_3, process_4, process_8, process_9, process_10, process_11, industrial sector, firm scale, pH, wastewater discharge amount, Ni
	XGB	0.50	118.12	2.66	22.94	-	1.4E+04	4.6E+07	process_1, process_2, process_3, process_4, process_8, process_9, process_10, process_11, industrial sector, firm scale, pH, wastewater discharge amount, Fe
	SVM	0.54	87.35	12.19	18.34	-	7.6E+03	2.5E+07	process_1, process_2, process_3, process_4, process_8, process_9, process_10, process_11, industrial sector, firm scale, COD, wastewater discharge amount
	KNN	0.45	106.43	0.94	15.58	-	1.1E+04	3.7E+07	process_1, process_2, process_3, process_4, process_8, process_9, process_10, process_11, industrial sector, firm scale, COD, N, Fe
	RF	0.85	47.50	1.40	13.34	-	2.3E+03	7.4E+06	process_1, process_2, process_3, process_4, process_8, process_9, process_10, process_11, industrial sector, firm scale, COD, pH, Ni, Fe
Deep Learning	MLP	0.67	68.63	13.86	22.41	-	4.7E+03	1.6E+07	process_1, process_2, process_3, process_4, process_8, process_9, process_10, process_11, industrial sector, firm scale, COD, pH, wastewater discharge amount, Ni, Fe, Cu, Zn
	Ensemble MLP	0.21	108.09	9.40	18.93	-	1.2E+04	3.8E+07	process_1, process_2, process_3, process_4, process_8, process_9, process_10, process_11, industrial sector, firm scale, COD, pH, wastewater discharge amount, Ni, Fe, Cu, Zn
	TNN	0.56	96.32	5.16	32.73	-	9.3E+03	3.1E+07	process_1, process_2, process_3, process_4, process_8, process_9, process_10, process_11, industrial sector, firm scale, COD, pH, wastewater discharge amount, NH_3-N, N, P, Ni, Fe, Cr, Cu, Zn, Cr^{VI}

Supplementary Table 5. Performance comparison of ensemble model and direct regression model to predict the generation quantity of MHW.

Model	Model Performance Evaluation Metric			
	The test dataset with MHW = 0		The test dataset with MHW > 0	
	Accuracy	F1	R^2	RMSE
Ensemble Model	0.96	0.95	0.88	62.51
Direct Regression	0.63	0.74	0.75	83.45

Supplementary Table 6. Model performance of 15 models, including a combined model and 9 sector-independent models to predict total generation quantity of HW, a combined ensemble model and 4 sector-independent ensemble models to predict generation quantity of MHW.

Models			Data count	R^2	RMSE	MAE	MAD	MAPE	MSE	SSE
Models to predict the total generation quantity of HW	The Combined model		3294	0.87	247.40	60.77	122.28	0.56	6.1E+04	2.0E+08
	Sector independent models	OCM	615	0.77	237.01	75.62	130.02	0.75	5.6E+04	3.5E+07
		CPM	363	0.70	368.42	212.06	233.97	0.62	1.4E+05	4.9E+07
		SCP	226	0.87	289.41	110.71	155.90	0.66	8.4E+04	1.9E+07
		SRP	229	0.93	366.70	104.83	200.80	0.83	1.3E+05	3.1E+07
		MWR	160	0.92	168.80	67.37	93.09	0.38	2.8E+04	4.6E+06
		MST	1326	0.86	31.92	10.60	18.03	0.44	1.0E+03	1.4E+06
		ECM	294	0.84	427.67	145.72	242.88	0.47	1.8E+05	5.4E+07
		BEG	46	0.92	297.76	184.78	206.16	0.33	8.9E+04	4.1E+06
		EGU	30	0.96	151.42	86.47	101.58	0.44	2.3E+04	6.9E+05
Models to predict the generation quantity of MHW	The Combined model		3294	0.85	53.67	0.92	13.32	-	2.9E+03	9.5E+06
	Sector independent models	SRP	229	0.64	36.92	11.98	18.47	-	1.4E+03	3.1E+05
		MWR	160	0.76	29.06	17.32	19.39	-	8.4E+02	1.4E+05
		MST	1326	0.72	30.68	9.09	16.25	-	9.4E+02	1.2E+06
		ECM	294	0.65	209.55	11.43	67.96	-	4.4E+04	1.3E+07

Supplementary Table 7. Descriptive statistics of total generation quantity (ton) of HW for 10 industrial sectors.

Sector	Mean	SD	Min	Percentiles			Max
				25%	50%	75%	
OCM	253.38	512.78	0.00	7.70	39.21	202.08	3.5E+03
CPM	442.88	740.23	0.03	31.45	181.70	511.98	6.6E+03
SCP	390.05	1135.25	0.01	5.37	39.28	246.04	9.7E+03
STE	995.74	1180.37	1.73	26.54	72.82	2392.96	2.6E+03
SRP	489.52	1412.70	0.01	16.33	75.54	309.05	1.2E+04
MWR	278.89	498.56	0.02	59.95	143.03	298.38	3.7E+03
MST	50.64	74.69	0.00	4.18	19.26	65.08	5.5E+02
ECM	666.82	1060.35	0.15	41.35	272.62	687.28	8.2E+03
BEG	987.54	722.75	0.01	609.06	875.25	1280.85	3.6E+03
EGU	717.26	618.59	0.03	89.09	642.61	1173.13	2.5E+03

Supplementary Table 8. Descriptive statistics of generation quantity (ton) of MHW for 10 industrial sectors.

Sector	Mean	SD	Min	Percentiles			Max
				25%	50%	75%	
OCM	0.42	5.44	0.00	0.00	0.00	0.00	129.78
CPM	0.00	0.00	0.00	0.00	0.00	0.00	0.00
SCP	0.06	1.33	0.00	0.00	0.00	0.00	33.38
STE	179.97	230.70	0.00	0.00	0.00	405.39	657.60
SRP	30.33	72.24	0.00	1.00	9.46	29.28	832.60
MWR	52.42	85.77	0.00	6.98	31.00	65.60	1061.06
MST	33.34	51.51	0.00	2.76	12.07	41.00	494.06
ECM	77.67	396.36	0.00	0.00	5.86	45.51	5274.05
BEG	0.00	0.00	0.00	0.00	0.00	0.00	0.00
EGU	3.87	11.86	0.00	0.00	0.00	0.00	72.16

Supplementary Table 9. Model performance of application cases to demonstrate the generalization of the model framework and discuss the spatial heterogeneity using the yearly data from the China Environmental Statistics Database of 2015.

Sector	Region	Cluster	Feature engineering method	R^2	RMSE	MAD	MAE	MAPE	MSE	SSE
Metal surface treatment	Zhejiang, China	High WTH	BL	0.74	705.41	238.33	342.08	22.35	5.0E+05	3.9E+07
			MB	0.66	798.75	273.35	392.18	19.09	6.4E+05	5.0E+07
			MB+I&I	0.72	721.15	240.36	348.32	15.53	5.2E+05	4.1E+07
	Guangdong, China	High WTH	BL	0.63	102.53	51.34	64.75	0.65	1.1E+04	1.9E+06
			MB	0.60	122.73	41.05	71.79	0.63	1.5E+04	2.8E+06
			MB+I&I	0.61	106.05	48.94	67.85	0.74	1.1E+04	2.1E+06
	Jiangsu*, China	Median WTH	BL	0.66	916.62	211.88	431.18	0.91	8.4E+05	7.7E+07
			MB	0.28	1238.77	171.52	474.89	1.01	1.5E+06	1.4E+08
			MB+I&I	0.71	903.94	211.21	439.80	1.01	8.2E+05	7.5E+07
	Fujian, China	Median WTH	BL	0.70	80.80	70.03	55.64	0.83	6.5E+03	1.4E+05
			MB	0.68	84.60	82.31	66.74	0.78	7.2E+03	1.6E+05
			MB+I&I	0.69	82.06	62.07	55.98	0.91	6.7E+03	1.5E+05
	Hebei, China	Median WTH	BL	0.77	171.57	46.11	103.18	0.74	2.9E+04	7.7E+05
			MB	0.68	191.36	43.08	116.00	0.79	3.7E+04	9.5E+05
			MB+I&I	0.72	188.01	38.59	105.51	0.78	3.5E+04	9.2E+05
	Shandong, China	Low WTH	BL	0.66	30.83	5.69	13.98	7.85	9.5E+02	3.6E+04
			MB	0.66	30.83	5.79	13.16	7.61	9.5E+02	3.6E+04
			MB+I&I	0.69	29.38	5.23	12.00	6.50	8.6E+02	3.3E+04
lead and zinc metallurgy	Hunan, China	-	BL	0.70	3072.10	1044.67	1925.85	39.20	9.4E+06	1.9E+08
			MB	0.80	2484.94	882.04	1505.48	32.94	6.2E+06	1.2E+08
			MB+I&I	0.82	2366.63	878.63	1549.32	36.76	5.6E+06	1.1E+08

Note: BL is the baseline model with all the features as input to train the model; MB is the model trained using the set of features selected based on Markov blanket; MB+I&I is the input features screened incorporating the causal discovery, importance, and correlation analysis.

* Even though the studied region was same as the dominant case in Section 3, the model here used the yearly dataset in 2015, rather than the monthly observations.

Supplementary Table 10. Performances of models to predict total generation quantity of HW under different feature selection methods.

Models		Feature engineering method	R^2	RMSE	MAD	MAE	MAPE	MSE	SSE	Input Features
The Combined model		BL	0.87	246.50	66.40	123.46	0.55	6.1E+04	2.0E+08	process_1, process_2, process_4, process_6, process_8, process_9, process_10, process_11, industrial sector, firm scale, pH, wastewater discharge amount, COD, Fe, Ni
		MB	0.68	386.35	67.26	167.53	0.61	1.5E+05	4.9E+08	
		MB+I&I	0.87	247.40	60.77	122.28	0.56	6.1E+04	2.0E+08	
Sector independent models	OCM	BL	0.78	230.95	103.41	132.93	0.83	5.3E+04	3.3E+07	process_5, process_6, process_7, process_8, process_9, process_10, process_11, firm scale, pH, wastewater discharge amount
		MB	0.76	241.47	76.91	133.47	0.74	5.8E+04	3.6E+07	
		MB+I&I	0.77	237.01	75.62	130.02	0.75	5.6E+04	3.5E+07	
	CPM	BL	0.73	359.68	271.24	253.69	0.60	1.3E+05	4.7E+07	process_5, process_6, process_7, process_8, process_9, process_10, process_11, pH, firm scale, N
		MB	0.53	471.03	248.28	271.68	0.64	2.2E+05	8.1E+07	
		MB+I&I	0.70	368.42	212.06	233.97	0.62	1.4E+05	4.9E+07	
	SCP	BL	0.84	295.85	119.64	160.09	0.66	8.8E+04	2.0E+07	process_2, process_3, process_5, process_6, process_7, process_8, process_9, process_10, process_11, firm scale, pH, P
		MB	0.91	246.21	77.00	128.09	0.67	6.1E+04	1.4E+07	
		MB+I&I	0.87	289.41	110.71	155.90	0.66	8.4E+04	1.9E+07	
	SRP	BL	0.95	338.86	126.72	195.83	0.86	1.1E+05	2.6E+07	process_1, process_2, process_3, process_9, process_10, process_11, firm scale, pH, N, Cr
		MB	0.93	381.42	162.41	217.68	0.77	1.5E+05	3.3E+07	
		MB+I&I	0.93	366.70	104.83	200.80	0.83	1.3E+05	3.1E+07	
	MWR	BL	0.95	130.36	101.61	89.48	0.42	1.7E+04	2.7E+06	process_1, process_2, process_3, process_4, process_9, process_10, process_11, firm scale, pH, P, Cu
		MB	0.93	150.88	85.72	92.75	0.41	2.3E+04	3.6E+06	
		MB+I&I	0.92	168.80	67.37	93.09	0.38	2.8E+04	4.6E+06	
	MST	BL	0.87	30.21	10.41	17.12	0.43	9.1E+02	1.2E+06	process_1, process_2, process_3, process_4, process_9, process_10, process_11, firm scale, pH, NH_3-N, Ni
		MB	0.86	32.34	12.29	18.75	0.47	1.0E+03	1.4E+06	
		MB+I&I	0.86	31.92	10.60	18.03	0.44	1.0E+03	1.4E+06	

Models	Feature engineering method	R^2	RMSE	MAD	MAE	MAPE	MSE	SSE	Input Features
ECM	BL	0.86	392.58	141.54	225.73	0.47	1.5E+05	4.5E+07	process_2, process_3, process_4, process_6, process_8, process_9, process_10, process_11, firm scale, pH, wastewater discharge amount, Ni
	MB	0.82	459.17	162.66	273.15	0.52	2.1E+05	6.2E+07	
	MB+I&I	0.84	427.67	145.72	242.88	0.47	1.8E+05	5.4E+07	
BEG	BL	0.87	417.87	252.28	272.96	0.36	1.7E+05	8.0E+06	process_2, process_3, process_6, process_7, process_9, process_10, process_11, firm scale, pH, COD
	MB	0.91	321.62	237.35	236.73	0.34	1.0E+05	4.8E+06	
	MB+I&I	0.92	297.76	184.78	206.16	0.33	8.9E+04	4.1E+06	
EGU	BL	0.95	177.88	67.31	110.46	0.46	3.2E+04	9.5E+05	process_2, process_3, process_4, process_6, process_8, process_9, process_10, process_11, firm scale, pH, NH_3-N, P, Cu
	MB	0.95	156.52	98.65	103.95	0.51	2.4E+04	7.3E+05	
	MB+I&I	0.96	142.62	77.98	92.64	0.51	2.0E+04	6.1E+05	

Note: BL is the baseline model with all the features as input to train the model; MB is the model trained using the set of features selected based on Markov blanket; MB+I&I is the input features screened incorporating the causal discovery, importance, and correlation analysis.

Supplementary Table 11. Performances of models to predict generation quantity of MHW under different feature selection methods.

Models		Feature engineering method	R^2	RMSE	MAD	MAE	MAPE	MSE	SSE	Input Features
The Combined model		BL	0.75	58.32	1.31	14.08	-	3.4E+03	1.1E+07	process_1, process_2, process_4, process_6, process_8, process_9, process_10, process_11, industrial sector, firm scale, pH, COD, Fe, Ni
		MB	0.67	83.08	1.03	14.86	-	6.9E+03	2.3E+07	
		MB+I&I	0.85	53.67	0.92	13.32	-	2.9E+03	9.5E+06	
Sector independent models	SRP	BL	0.62	38.37	15.87	19.83	-	1.5E+03	3.4E+05	process_1, process_2, process_3, process_9, process_10, process_11, firm scale, pH, NH_3-N, Ni, Cr
		MB	0.59	40.95	11.55	19.58	-	1.7E+03	3.8E+05	
		MB+I&I	0.64	36.92	11.98	18.47	-	1.4E+03	3.1E+05	
	MWR	BL	0.74	30.30	20.32	20.77	-	9.2E+02	1.5E+05	process_1, process_2, process_3, process_4, process_9, process_10, firm scale, pH, P, Zn, Ni
		MB	0.77	27.99	16.14	18.73	-	7.8E+02	1.3E+05	
		MB+I&I	0.76	29.06	17.32	19.39	-	8.4E+02	1.4E+05	
	MST	BL	0.73	29.50	8.88	15.33	-	8.7E+02	1.2E+06	process_1, process_2, process_3, process_9, process_10, process_11, firm scale, pH, wastewater discharge amount, Ni, Cu, Fe, Cr, Cr^{VI}
		MB	0.69	32.06	10.11	17.21	-	1.0E+03	1.4E+06	
		MB+I&I	0.72	30.68	9.09	16.25	-	9.4E+02	1.2E+06	
	ECM	BL	0.63	219.51	9.79	70.23	-	4.8E+04	1.4E+07	process_1, process_2, process_3, process_4, process_6, process_9, process_10, process_11, firm scale, pH, COD, NH_3-N, Ni, Cu
		MB	0.38	308.36	10.86	84.51	-	9.5E+04	2.8E+07	
		MB+I&I	0.65	209.55	11.43	67.96	-	4.4E+04	1.3E+07	

Note: BL is the baseline model with all the features as input to train the model; MB is the model trained using the set of features selected based on Markov blanket; MB+I&I is the input features screened incorporating the causal discovery, importance, and correlation analysis.

Supplementary Table 12. Uncertainty analysis results about the percentage of the test samples that have their true values fall into the respective 95% confidence intervals of prediction for different models

Models			N_test	N_in interval	proportion
Predicting the generation quantity of MHW	The combined model		3294	2914	88.46%
	Sector independent models	SRP	229	203	88.65%
		MWR	160	144	90.00%
		MST	1326	1189	89.67%
		ECM	294	273	92.86%
Predicting the total generation quantity of HW	The combined model		3294	3149	95.60%
	Sector independent models	OCM	615	563	91.54%
		CPM	363	335	92.29%
		SCP	226	216	95.58%
		SRP	229	214	93.45%
		MWR	160	152	95.00%
		MST	1326	1280	96.53%
		ECM	294	263	89.46%
		BEG	46	43	93.48%
		EGU	30	29	96.67%

Note: N_test refers to the amount of data in the model test set; N_in interval refers to the amounts of samples that have their true values fall into the respective 95% confidence intervals of prediction.

Supplementary Table 13. Firm scale categories based on staff number.

Staff Number	Category
1-49	I
50-99	II
100-199	III
200-499	IV
≥500	V

Supplementary Table 14. Main relevant manufacturing processes that generate hazardous waste for the 50 industrial sectors with the highest hazardous waste generation in China.

	Process name	Process abbreviations	Process description	Relevant industrial sectors
1	Machining Process	Process_1	Metal cutting, clipping, chipping, etc.[107-109]	Metal and Electronics Manufacturing Industry
2	Metal Heat Treatment	Process_2	Metal jointing, melting, quenching, etc.[110,111]	
3	Metal Surface Processing	Process_3	Metal surface plating, oxidation, phosphating, painting, coating, etc.[112-114]	
4	Circuit Board Processing	Process_4	Circuit board manufacturing process, including etching, exposition, covering photosensitive materials, etc.[115-118]	
5	Rectification and Distillation	Process_5	Rectification, distillation, refining[119]	Chemical Manufacturing Industry
6	Other Separations	Process_6	Separation methods such as filtration, pressure filtration, dehydration, etc.[120,121]	
7	Chemical Reaction	Process_7	Including oxidation, hydrogenation, hydrolysis, polymerization and other chemical reactions.[122,123]	
8	Incineration	Process_8	Incineration treatment[124,125]	General Segment
11	Equipment Maintenance	Process_9	Equipment replacement, maintenance, wiping, lubricating oil, etc.[126-128]	
9	Wastewater Treatment	Process_10	Waste liquid treatment, wastewater treatment, sludge treatment[129,130]	
10	Flue Gas Treatment	Process_11	Collection and treatment of oil mist, VOCs, dust, etc.[131,132]	
12	Mineral Leaching	Process_12	A variety of acids, cyanides, oxygen pressure, etc. are used to leach the metal elements in the ore.[133,134]	Mining Industry
13	Roasting	Process_13	The ore and salt are calcined at high temperature for ion exchange.[135,136]	
14	Electrolysis	Process_14	Electrolytic method for ion exchange to extract metal.[137,138]	
15	Amalgamation And Mercury Removal	/	Gold extraction and mercury removal process based on mixed mercury method.[139,140]	
16	Petroleum Collection	/	Including drilling and petroleum exploration segments.[141-143]	Petroleum Industry
17	Crude Oil Transportation	/	Transfer of petroleum and crude oil, etc.[144-146]	

Note: The manufacturing processes which have process abbreviations were used as model inputs in our studied cases.

574 Supplementary Table 15. 50 industrial sectors with highest HW generation
575 quantity in China.

	Industrial sector	Main category of hazardous waste	Region with highest HW generation (Province)	Main indicators related to wastewater emission	Hazardous waste related processes
1	Mining and dressing of asbestos and mica	HW36	Qinghai, Xinjiang	wastewater discharge amount, pH, COD, NH_3-N, SS, P, N, petroleum hydrocarbon pollutants, BOD, volatile phenol, sulphate, fluoride, cyanide[147]	Mineral Leaching, Waste Gas Treatment
2	Machine-made paper and cardboard manufacturing	HW35	Shandong, Hunan, Jiangxi	wastewater discharge amount, pH, COD, NH_3-N, SS, P, N, BOD[148]	Wastewater Treatment, Other Separations
3	Lead and zinc metallurgy	HW48	Yunnan, Hunan, Inner Mongolia	wastewater discharge amount, pH, COD, NH_3-N, N, P, Pb, As, Cd, Hg, Zn, Cu, Cr, Ni[149]	Mineral Leaching, Electrolysis, Roasting, Waste Gas Treatment
4	Gold mining	HW33	Qinghai, Shandong, Hunan	wastewater discharge amount, pH, COD, NH_3-N, N, P, Pb, As, Cd, Hg, Zn, Cu[149]	Wastewater Treatment, Mineral Leaching
5	Pigment manufacturing	HW12, HW34	Guangxi, Sichuan, Anhui	wastewater discharge amount, pH, COD, NH_3-N, SS, P, N, BOD, TOC, petroleum hydrocarbon pollutants[150]	Chemical Reaction, Other Separations, Wastewater Treatment
6	Organic chemical materials manufacturing	HW11	Zhejiang, Jiangsu, Shandong	wastewater discharge amount, pH, COD, NH_3-N, SS, P, N, BOD, petroleum hydrocarbon pollutants, sulphate[151]	Wastewater Treatment, Rectification, Other Separations, Chemical Reaction
7	Electricity generation using other sources	HW18, HW50, HW13, HW08	Zhejiang, Hubei, Fujian	wastewater discharge amount, pH, COD, NH_3-N,	Equipment Maintenance, Incineration

	Industrial sector	Main category of hazardous waste	Region with highest HW generation (Province)	Main indicators related to wastewater emission	Hazardous waste related processes
	Biomass energy generation			SS, P, volatile phenol, sulphate, petroleum hydrocarbon pollutants, fluoride[152]	Equipment Maintenance, Incineration
8	Oil extraction	HW08	Xinjiang, Shaanxi, Liaoning	wastewater discharge amount, pH, COD, NH_3-N, SS, P, BOD, TOC, petroleum hydrocarbon pollutants, sulphate[153]	Petroleum Collection, Crude Oil Transportation
9	Gold smelting	HW35, HW34	Shandong, Henan, Shaanxi	wastewater discharge amount, pH, COD, NH_3-N, P, N, Pb, As, Cd, Hg, Zn, Cu[149]	Mineral Leaching, Amalgamation and Mercury Removal
10	Crude oil processing and petroleum product manufacturing	HW35, HW50, HW11, HW08	Xinjiang, Liaoning, Hunan	wastewater discharge amount, pH, COD, NH_3-N, SS, N, P, BOD, TOC, petroleum hydrocarbon pollutants, volatile phenol, sulphate, fluoride[154]	Crude Oil Transportation, Rectification, Catalytic Reaction
11	Electronic circuits manufacturing	HW17, HW22	Guangdong, Jiangsu, Hubei	wastewater discharge amount, pH, COD, NH_3-N, SS, N, P, BOD, TOC, petroleum hydrocarbon pollutants, volatile phenol, fluoride, cyanide, Cu, Zn[155]	Metal Heat Treatment, Machining Process, Circuit Board Processing, Wastewater Treatment
12	Acrylic fiber manufacturing	HW38	Jilin, Liaoning, Jiangsu	wastewater discharge amount, pH, COD, NH_3-N, SS, N, P, BOD, TOC, petroleum hydrocarbon pollutants, volatile phenol, sulphate[156]	Wastewater Treatment, Rectification, Waste Gas Treatment

	Industrial sector	Main category of hazardous waste	Region with highest HW generation (Province)	Main indicators related to wastewater emission	Hazardous waste related processes
13	Inorganic salt manufacturing	HW46	Guizhou, Yunnan, Hubei	wastewater discharge amount, pH, COD, NH_3-N, SS, P, petroleum hydrocarbon pollutants, sulphate, fluoride, cyanide[157]	Chemical Reaction, Wastewater Treatment
14	Steel rolling and processing	HW17, HW34	Hebei, Jiangsu, Guangdong	wastewater discharge amount, pH, COD, NH_3-N, SS, P, N, petroleum hydrocarbon pollutants[158]	Machining Process, Wastewater Treatment, Surface Processing
15	Chemical drug API manufacturing	HW02, HW50	Inner Mongolia, Shandong, Zhejiang	wastewater discharge amount, pH, COD, NH_3-N, SS, P, N, BOD, TOC, cyanide, volatile phenol, Cu, Zn[159]	Wastewater Treatment, Rectification, Other Separations, Chemical Reaction
16	Manufacturing of other basic chemical raw materials	HW06	Shandong, Jiangxi, Inner Mongolia	wastewater discharge amount, pH, COD, NH_3-N, SS, P, petroleum hydrocarbon pollutants, sulphate, fluoride, cyanide[157]	Wastewater Treatment, Rectification, Other Separations, Chemical Reaction
17	Thermal power generation	HW12, HW50	Zhejiang, Yunnan, Jiangsu	wastewater discharge amount, pH, COD, NH_3-N, SS, P, volatile phenol, sulphate, petroleum hydrocarbon pollutants, fluoride[152]	Incineration, Waste Gas Treatment
18	Phosphate fertilizer manufacturing	HW37	Jiangsu, Anhui, Hubei	wastewater discharge amount, pH, COD, NH_3-N, SS, P, N, fluoride[160]	Mineral Leaching, Chemical Reaction
19	Manufacturing of wood and bamboo pulp	HW35	Shandong, Hainan, Guangxi	wastewater discharge amount, pH,	Other Separations

	Industrial sector	Main category of hazardous waste	Region with highest HW generation (Province)	Main indicators related to wastewater emission	Hazardous waste related processes
				COD, NH_3-N, SS, P, N, BOD[148]	
20	Metal surface treatment and heat treatment	HW34, HW17	Zhejiang, Jiangsu, Guangdong	wastewater discharge amount, pH, COD, NH_3-N, Cu, Zn, Fe, petroleum hydrocarbon pollutants, fluoride, cyanide[161,162]	Surface Processing, Wastewater Treatment
21	Copper smelting	HW12, HW09, HW48, HW08	Inner Mongolia, Yunnan, Gansu	wastewater discharge amount, pH, COD, NH_3-N, P, N, Pb, As, Cd, Hg, Zn, Cu, Ni[149]	Metals Leaching, Waste Gas Treatment, Amalgamation And Mercury Removal
22	Steelmaking	HW31, HW09, HW23	Hunan, Fujian, Shandong	wastewater discharge amount, pH, COD, NH_3-N, N, petroleum hydrocarbon pollutants[158]	Equipment Maintenance, Incineration, Waste Gas Treatment
23	Coking	HW11	Shanxi, Inner Mongolia, Hebei	wastewater discharge amount, pH, COD, NH_3-N, SS, P, N, BOD, petroleum hydrocarbon pollutants, volatile phenol, cyanide, sulphate[158]	Waste Gas Treatment, Wastewater Treatment, Rectification
24	Primary form plastic and synthetic resin manufacturing	HW06	Shandong, Guangdong, Inner Mongolia	wastewater discharge amount, pH, COD, NH_3-N, SS, P, N, BOD, petroleum hydrocarbon pollutants, sulphate[151,163]	Rectification, Chemical Reaction, Wastewater Treatment
25	Iron making	HW08, HW48	Hebei, Shandong, Xinjiang	wastewater discharge amount, pH, COD, NH_3-N, SS, N, petroleum hydrocarbon pollutants[158]	Waste Gas Treatment, Roasting, Equipment Maintenance

	Industrial sector	Main category of hazardous waste	Region with highest HW generation (Province)	Main indicators related to wastewater emission	Hazardous waste related processes
26	Aluminum smelting	HW13, HW08, HW17, HW48	Xinjiang, Qinghai, Henan	wastewater discharge amount, pH, COD, NH_3-N, P, N, fluoride[149]	Roasting, Waste Gas Treatment, Electrolysis
27	Metal scrap and scrap processing	HW17	Yunnan, Zhejiang, Guangdong	wastewater discharge amount, pH, COD, NH_3-N, P, N, Cu, Zn[164]	Mineral Leaching, Metal Heat Treatment, Machining Process
28	Vinylon fiber manufacturing	HW12, HW34, HW35	Chongqing, Zhejiang, Shanghai	wastewater discharge amount, pH, COD, NH_3-N, SS, P, N, BOD, TOC, petroleum hydrocarbon pollutants, volatile phenol, sulphate[156]	Wastewater Treatment
29	Automobile manufacturing	HW08, HW36	Chongqing, Shanghai, Jilin	pH, SS, P, N, COD, BOD, NH_3-N[165]	Machining Process, Metal Heat Treatment
30	Other battery manufacturing	HW23, HW29, HW31	Jiangsu, Anhui, Zhejiang	wastewater discharge amount, pH, COD, NH_3-N, SS, P, N, fluoride[166]	Wastewater Treatment, Electrolysis, Wastewater Treatment
31	Information chemical manufacturing	HW06, HW24, HW11	Inner Mongolia, Liaoning, Jiangsu	pH, COD, NH_3-N, SS, P, N, BOD[167]	Circuit Board Processing
32	Synthetic fiber mono (polymer) body manufacturing	HW06, HW38, HW11	Hunan, Shandong, Hebei	wastewater discharge amount, COD, NH_3-N[156]	Wastewater Treatment
33	Dye manufacturing	HW08, HW12	Zhejiang, Jiangsu, Ningxia	wastewater discharge amount, pH, COD, NH_3-N, SS, P, N, BOD, sulphate[168]	Wastewater Treatment, Chemical Reaction
34	Manufacturing of architectural ceramic products	HW12	Guangdong, Fujian, Liaoning	wastewater discharge amount, pH, COD, NH_3-N[169]	Roasting, Waste Gas Treatment, Surface Processing
35	Manufacturing of optoelectronic devices and other electronic devices	HW13, HW12, HW09, HW06	Zhejiang, Guangdong, Jiangsu	wastewater discharge amount, pH, COD, NH_3-N, SS, P, N, petroleum hydrocarbon	Surface Processing, Circuit Board Processing

	Industrial sector	Main category of hazardous waste	Region with highest HW generation (Province)	Main indicators related to wastewater emission	Hazardous waste related processes
				pollutants, TOC, cyanide, sulphate, fluoride, Cu, Zn[155]	
36	Auto parts and accessories manufacturing	HW36	Jiangsu, Guangdong, Beijing	pH, SS, COD, BOD, NH_3-N, P, N[165]	Machining Process, Metal Heat Treatment
37	Chemical pesticides Manufacturing	HW04, HW11	Jiangsu, Shandong, Liaoning	wastewater discharge amount, pH, COD, NH_3-N, SS, P, BOD, petroleum hydrocarbon pollutants, volatile phenol[170]	Equipment Maintenance, Rectification, Other Separations, Chemical Reaction
38	Tin smelting	HW48	Yunnan, Guangxi, Hunan	wastewater discharge amount, pH, COD, NH_3-N, P, N, Pb, As, Cd, Hg, Zn, Cu, Cr^{VI}[149]	Mineral Leaching, Electrolysis
39	Electronic components and component manufacturing	HW17, HW09	Jiangsu, Guangdong, Tianjin	wastewater discharge amount, pH, COD, NH_3-N, SS, P, N, petroleum hydrocarbon pollutants, TOC, cyanide, sulphate, fluoride, Cu, Zn[155]	Circuit Board Processing, Surface Processing
40	Metal wire and rope Manufacturing	HW08, HW17, HW09, HW34	Jiangsu, Zhejiang, Hunan	pH, SS, COD, BOD, NH_3-N, P, N[165]	Wastewater Treatment, Machining Process
41	Paint manufacturing	HW12, HW09, HW06	Anhui, Shanghai, Jiangsu	wastewater discharge amount, pH, COD, NH_3-N, P, N, SS, BOD, TOC, petroleum hydrocarbon pollutants[150]	Wastewater Treatment, Chemical Reaction
42	Manufacturing of chemical reagents and auxiliaries	HW06, HW11	Shandong, Jiangsu, Zhejiang	wastewater discharge amount, pH, COD, NH_3-N, SS, P, petroleum	Wastewater Treatment, Rectification, Other Separations, Chemical Reaction

	Industrial sector	Main category of hazardous waste	Region with highest HW generation (Province)	Main indicators related to wastewater emission	Hazardous waste related processes
				hydrocarbon pollutants, sulphate, fluoride, cyanide[157]	
43	Integrated circuit manufacturing	HW34	Jiangsu, Shanghai, Shaanxi	wastewater discharge amount, pH, COD, NH_3-N, SS, P, N, petroleum hydrocarbon pollutants, TOC, cyanide, sulphate, fluoride, Cu, Zn[155]	Metal Heat Treatment, Machining Process, Circuit Board Processing, Wastewater Treatment
44	Fabrication of metal structures	HW17, HW34	Jiangsu, Guangdong, Anhui	pH, SS, COD, BOD, NH_3-N, P, N[165]	Mineral Leaching, Metal Heat Treatment, Machining Process
45	Copper rolling	HW22	Jiangxi, Hubei, Zhejiang	wastewater discharge amount, pH, COD, NH_3-N, P, N, Pb, As, Cd, Hg, Zn, Cu, Ni[165]	Machining Process, Metal Heat Treatment
46	Specialized chemical products manufacturing	HW06, HW34, HW11	Jiangsu, Zhejiang, Xinjiang	wastewater discharge amount, pH, COD, NH_3-N, P, N, SS, BOD, TOC, petroleum hydrocarbon pollutants, sulphate, fluoride, cyanide, volatile phenol, Cu, Zn[171]	Rectification, Other Separations, Chemical Reaction
47	Inorganic alkali manufacturing	HW50, HW34, HW11	Jiangsu, Hubei, Fujian	wastewater discharge amount, pH, COD, NH_3-N, SS, P, petroleum hydrocarbon pollutants, sulphate, fluoride, cyanide[157]	Wastewater Treatment, Rectification, Other Separations, Equipment Maintenance
48	Nickel and cobalt smelting	HW06, HW46, HW09, HW08	Gansu, Jiangxi, Yunnan	wastewater discharge amount, pH, COD, NH_3-N, P, N, Pb, As, Cd,	Metals Leaching, Electrolysis, Roasting

	Industrial sector	Main category of hazardous waste	Region with highest HW generation (Province)	Main indicators related to wastewater emission	Hazardous waste related processes
				Hg, Zn, Cu, Ni[149]	
49	Other electronic equipment manufacturing	HW17	Jiangsu, Anhui, Sichuan	wastewater discharge amount, pH, COD, NH_3-N, SS, P, N, petroleum hydrocarbon pollutants, TOC, cyanide, sulphate, fluoride, Cu, Zn[155]	Surface Processing, Circuit Board Processing
50	Ferroalloy smelting	HW21	Liaoning, Hunan, Sichuan	wastewater discharge amount, pH, COD, NH_3-N, N, petroleum hydrocarbon pollutants[158]	Metals Leaching, Electrolysis, Roasting

Note: The major types of hazardous waste generated from different sectors are mainly derived from the Chinese National List of Hazardous Wastes. Region with highest HW generation from different industrial sectors were summarized according to the China Environmental Statistics Database.

Supplementary Table 16. Comparison of hazardous waste list across China, U.S. and EU.

Hazardous waste name[172]	Hazardous waste code		
	Chinese national list of hazardous wastes (China)[172]	Identification and listing of hazardous wastes (U.S.)[173]	Commission notice on technical guidance on the classification of waste (EU)[174]
Healthcare wastes	HW01		
Pharmaceutical wastes	HW02	K084, K101, K102	07 05 01, 07 07 04, 07 07 08, 07 07 10, 07 07 11, 18 01 03
Obsolete medicine	HW03		18 01 08, 18 02 02, 18 02 07, 20 01 31
Pesticide wastes	HW04	K158, K159, K161, K031, K032, K033, K034, K035, K036, K037, K038, K039, K040, K041, K042, K043, K097, K098, K099, K123, K124, K125, K126, K132 F020	02 01 08, 06 13 01, 20 01 19, 07 04 01, 07 04 04, 07 04 08, 07 04 10, 07 04 11, 07 04 13
Wastes containing wood preservation chemicals	HW05	K001 F032, F034, F035	03 01 04, 03 02 01, 03 02 02, 03 02 03, 03 02 04, 03 02 05, 17 02 04, 19 12 06
Organic solvent-containing wastes	HW06	F001, F002, F003, F004, F005	07 01 04, 07 02 04, 10 09 15, 10 10 15, 14 06 03, 14 06 05, 20 01 13
Wastes from heat treatment containing cyanide	HW07	F007, F008, F009, F011, F012	06 03 11
Waste mineral oils and waste containing mineral oils	HW08	K048, K049, K050, K051, K052, K169, K170 F010	01 05 05, 01 05 06, 05 01 02, 05 01 03, 05 01 05, 05 01 06, 05 01 09, 05 01 11, 05 01 15, 07 02 01, 12 01 07, 13 01 01, 13 01 09, 13 01 10, 13 01 11, 13 01 12, 13 01 13, 13 02 04, 13 02 05, 13 02 06, 13 02 07, 13 02 08, 13 03 01, 13 03 06, 13 03 07, 13 03 09, 13 03 10, 13 04 01, 13 04 02, 13 04 03, 13 05 06, 13 07 01, 13 07 02, 13 07 03, 16 07 08, 19 02 07, 19 11 01
Oil/water, hydrocarbon/water mixture or emulsified liquid	HW09	F037, F038	12 01 09, 12 01 10, 12 01 12, 12 01 19, 12 03 01, 12 03 02, 13 01 04, 13 01 05, 13 05 07, 13 05 08, 13 08 01, 13 08 02, 13 08 99, 19 08 10
Wastes containing PCBs and PBBs	HW10	F022, F026	16 01 09, 16 02 09, 16 02 10, 17 09 02
Residues of rectifying and distillation	HW11	K009, K010, K015, K016, K017, K018, K019, K020, K022, K023, K024, K025,	05 01 07, 05 01 08, 05 01 12, 05 06 01, 05 06 03, 06 13 05, 17 03 01, 17 03 03,

Hazardous waste name[172]	Hazardous waste code		
	Chinese national list of hazardous wastes (China)[172]	Identification and listing of hazardous wastes (U.S.)[173]	Commission notice on technical guidance on the classification of waste (EU)[174]
		K026, K027, K029, K030, K083, K085, K093, K094, K095, K096, K103, K104, K105, K107, K108, K109, K110, K111, K112, K113, K114, K115, K116, K117, K118, K136, K149, K150, K151, K156, K157, K060, K087, K141, K142, K143, K144, K145, K147, K148<br>F024	19 11 02, 19 11 03, 19 11 05
Waste dyes and paints	HW12	K002, K003, K004, K005, K006, K007, K008, K181, K086	04 02 14, 04 02 16, 04 02 19, 07 03 01, 07 03 04, 07 03 08, 07 03 10, 07 03 11, 08 01 11, 08 01 13, 08 01 15, 08 01 17, 08 01 19, 08 01 21, 08 03 12, 08 03 14, 08 03 16, 08 03 17, 08 03 19, 10 12 11
Organic resins wastes	HW13		08 04 09, 08 04 11, 08 04 13, 08 04 15, 08 04 17, 10 09 13, 10 10 13, 11 01 16, 19 08 06
Wastes containing unknown chemicals	HW14		
Explosive wastes	HW15		12 01 16, 16 04 01, 16 04 02, 16 04 03
Photographic chemical wastes	HW16		09 01 01, 09 01 02, 09 01 03, 09 01 04, 09 01 05, 09 01 06
Metal surface treatment wastes	HW17	F006, F019	06 03 13, 06 03 15, 06 04 05, 06 05 02, 06 06 02, 11 01 08, 11 01 09, 11 01 11, 11 01 13, 11 01 15, 11 01 98, 11 05 03, 11 05 04, 12 01 14, 12 01 18, 12 01 20
Residues of incinerating disposal	HW18	F028	19 01 05, 19 01 06, 19 01 07, 19 01 11, 19 01 13, 19 01 15, 19 01 17, 19 04 02, 19 04 03
Metal carbonyl compound wastes	HW19		
Beryllium wastes	HW20		
Chromium wastes	HW21		04 01 03, 16 09 02
Copper wastes	HW22		10 06 03, 10 06 06, 10 06 07, 10 06 09, 10 11 11, 10

Hazardous waste name[172]	Hazardous waste code		
	Chinese national list of hazardous wastes (China)[172]	Identification and listing of hazardous wastes (U.S.)[173]	Commission notice on technical guidance on the classification of waste (EU)[174]
			11 13, 10 11 15, 10 11 17, 10 11 19, 11 02 05
Zinc wastes	HW23	K061	10 05 03, 10 05 05, 10 05 06, 10 05 08, 10 05 10, 11 02 02
Arsenic wastes	HW24		06 04 03, 10 04 03
Selenium wastes	HW25		
Cadmium wastes	HW26		
Antimony wastes	HW27	K021, K176, K177	
Tellurium wastes	HW28		
Mercury wastes	HW29	K174, K175, K071, K106	05 07 01, 06 04 04, 06 07 02, 06 07 03, 10 14 01, 16 01 08, 16 03 07, 16 06 03, 17 09 01, 18 01 10, 20 01 21
Thallium wastes	HW30		10 04 01, 10 04 02, 10 04 04, 10 04 05, 10 04 06, 10 04 07, 10 04 09
Lead wastes	HW31	K069, K100	
Inorganic fluoride wastes	HW32		
Inorganic cyanide wastes	HW33		11 03 01
Waste acids	HW34	K178, K131, K062	05 01 04, 06 01 01, 06 01 02, 06 01 03, 06 01 04, 06 01 05, 06 01 06, 06 07 04, 10 01 09, 11 01 05, 11 01 06, 11 01 07, 20 01 14
Waste alkali	HW35		06 02 01, 06 02 03, 06 02 04, 06 02 05, 06 09 03, 20 01 15
Asbestos wastes	HW36		01 04 07, 06 07 01, 06 13 04, 10 13 09, 10 13 12, 16 01 11, 16 02 12, 16 11 01, 16 11 03, 16 11 05, 17 06 01, 17 06 05, 17 08 01
Organic phosphorus wastes	HW37		
Organic cyanide wastes	HW38	K011, K013, K014	08 05 01
Phenols wastes	HW39	F021, F023, F027	
Ethers wastes	HW40		
Organohalogen compound wastes	HW45	K073	06 10 02, 07 01 03, 07 01 07, 07 01 08, 07 01 09, 07 01 10, 07 01 11, 07 02 03, 07 02 07, 07 02 08, 07 02 09, 07 02 10, 07 02 11, 07 02 14, 07 03 03, 07 03 07,

Hazardous waste name[172]	Hazardous waste code		
	Chinese national list of hazardous wastes (China)[172]	Identification and listing of hazardous wastes (U.S.)[173]	Commission notice on technical guidance on the classification of waste (EU)[174]
			07 03 09, 07 04 03, 07 04 07, 07 04 09, 07 07 03, 07 07 07, 07 07 09, 12 01 06, 12 01 08, 14 06 01, 14 06 02, 14 06 04, 16 02 11, 20 01 23
Nickel compound wastes	HW46		
Barium compound wastes	HW47		
Non-ferrous metal smelting wastes	HW48	K088	01 03 04, 01 03 05, 01 03 07, 10 02 07, 10 02 11, 10 02 13, 10 03 04, 10 03 08, 10 03 09, 10 03 15, 10 03 17, 10 03 19, 10 03 21, 10 03 23, 10 03 25, 10 03 27, 10 03 29, 10 07 07, 10 08 08, 10 08 10, 10 08 12, 10 08 15, 10 08 17, 10 08 19, 10 09 05, 10 09 07, 10 09 09, 10 09 11, 10 10 05, 10 10 07, 10 10 09, 10 10 11, 11 02 07
Other hazardous wastes not listed	HW49	F039	06 08 02, 06 13 02, 07 01 01, 15 01 10, 07 02 16, 15 01 11, 16 05 06, 16 06 06, 19 01 10, 19 02 05
Catalyst wastes	HW50	K028, K171, K172 F025	16 08 02, 16 08 05, 16 08 06, 16 08 07

583

Supplementary Table 17. Names and abbreviations of 10 industrial sectors included in this study.

Sector Name	Abbreviation
Organic Chemical Materials Manufacturing	OCM
Chemical Pesticides Manufacturing	CPM
Specialized Chemical Products Manufacturing	SCP
Steelmaking	STE
Steel Rolling and Processing	SRP
Metal Wire and Rope Manufacturing	MWR
Metal Surface Treatment	MST
Electronic Circuits Manufacturing	ECM
Biomass Energy Generation	BEG
Electricity Generation Using other sources	EGU

Supplementary Table 18. Descriptive statistics of numeric variables for cleaned 16,477 observations.

Variables[a]	Mean	SD[b]	Min	Percentiles			Max
				25%	50%	75%	
COD (kg)	1294.85	12929.26	0.00	19.30	93.57	492.05	9.9E+05
pH	7.52	0.39	2.21	7.35	7.48	7.68	1.2E+01
Wastewater discharge amount (t)	12323.97	46115.34	0.00	614.00	2286.00	8205.00	1.8E+06
P (kg)	1405.86	121179.90	0.00	0.06	0.32	1.77	1.2E+07
NH_3-N (kg)	44.12	395.39	0.00	0.34	1.73	11.34	3.6E+04
N (kg)	93.38	300.98	0.00	3.57	14.79	62.68	7.7E+03
Cr^{VI} (kg)	0.09	3.74	0.00	0.00	0.00	0.01	4.7E+02
Cr (kg)	0.08	1.50	0.00	0.00	0.00	0.03	1.9E+02
Ni (kg)	1.30	54.86	0.00	0.00	0.01	0.05	4.8E+03
Fe (kg)	0.84	2.57	0.00	0.00	0.00	0.52	6.1E+01
Cu (kg)	0.50	14.41	0.00	0.00	0.02	0.13	1.8E+03
Zn (kg)	2.40	279.16	0.00	0.00	0.00	0.13	3.6E+04
Total generation quantity of HW (t)	272.66	717.50	0.00	7.79	44.91	204.14	1.2E+04
Generation quantity of MHW (t)	25.40	128.31	0.00	0.00	0.86	20.28	5.3E+03

[a] Monthly total; [b] Standard deviation

Supplementary Table 19. Descriptive statistics of numeric variables for all the observations of the application case for metal surface treatment sector in Zhejiang, China.

Variables	Mean	SD	Min	Percentiles			Max
				25%	50%	75%	
Wastewater discharge amount (t)	49946.28	75778.92	187.00	13560.00	30929.70	64850.00	1160000.00
COD (kg)	4.41	12.43	0.02	0.80	2.03	5.03	232.00
NH_3-N (kg)	0.34	0.68	0.00	0.03	0.12	0.44	9.20
N (kg)	0.79	1.17	0.00	0.08	0.35	1.01	9.20
P (kg)	0.10	0.29	0.00	0.00	0.02	0.09	3.70
Cr (kg)	9.01	16.87	0.00	0.40	2.54	10.82	126.00
Cr^{VI}(kg)	3.18	5.20	0.00	0.20	0.99	3.55	29.00
Total generation quantity of HW (t)	509.11	1456.97	0.17	33.75	169.44	500.38	18502.80

Supplementary Table 20. Descriptive statistics of numeric variables for all the observations of the application case for metal surface treatment sector in Shandong, China.

Variables	Mean	SD	Min	Percentiles			Max
				25%	50%	75%	
Wastewater discharge amount (t)	32039.51	40478.62	250.00	4962.50	17258.58	39675.00	280000.00
COD (kg)	1.62	3.51	0.00	0.19	0.46	1.84	39.40
NH_3-N (kg)	0.12	0.18	0.00	0.01	0.04	0.16	1.13
N (kg)	0.22	0.54	0.00	0.02	0.08	0.22	4.99
P (kg)	0.02	0.05	0.00	0.00	0.01	0.02	0.63
Cr (kg)	7.19	15.89	0.00	0.00	0.27	2.65	83.66
Cr^{VI}(kg)	5.88	13.47	0.00	0.00	0.14	2.79	75.81
Total generation quantity of HW (t)	39.88	269.54	0.01	0.67	1.15	3.36	3540.16

Supplementary Table 21. Descriptive statistics of numeric variables for all the observations of the application case for lead and zinc metallurgy sector in Hunan, China.

Variables	Mean	SD	Min	Percentiles			Max
				25%	50%	75%	
Wastewater discharge amount (t)	94367.28	223727.35	93.55	5444.85	17264.94	47700.00	1293541.00
COD (kg)	6.03	15.24	0.00	0.00	0.26	3.14	94.88
NH_3-N (kg)	1.20	2.72	0.00	0.07	0.49	0.69	18.10
N (kg)	1.91	3.53	0.00	0.11	0.91	1.17	18.10
P (kg)	1.61	4.88	0.00	0.00	0.00	0.00	27.66
Cr (kg)	0.94	2.35	0.00	0.04	0.15	0.38	13.88
Cr^{VI}(kg)	0.94	2.35	0.00	0.04	0.15	0.38	13.88
Total generation quantity of HW (t)	6885.22	25846.01	0.63	41.38	293.95	2254.11	208256.00

Supplementary Table 22. Descriptive statistics of numeric variables for all the observations of the application case for metal surface treatment sector in Jiangsu, China.

Variables	Mean	SD	Min	Percentiles			Max
				25%	50%	75%	
Wastewater discharge amount (t)	49948.89	111083.99	10.00	7749.25	20010.00	46100.00	1102064.00
COD (kg)	4.01	8.90	0.00	0.40	1.30	3.64	87.50
NH_3-N (kg)	0.24	0.86	0.00	0.01	0.05	0.20	13.75
N (kg)	0.53	1.52	0.00	0.04	0.15	0.49	18.00
P (kg)	0.06	0.13	0.00	0.00	0.02	0.05	1.19
Cr (kg)	9.10	18.10	0.00	0.10	1.86	10.64	157.65
Cr^{VI}(kg)	4.90	9.79	0.00	0.05	1.04	5.04	102.40
Total generation quantity of HW (t)	328.77	2697.53	0.09	4.79	18.90	79.20	55012.17

Note: The yearly data is derived from the China Environmental Statistics Database of 2015, different from the monthly observations.

Supplementary Table 23. Descriptive statistics of numeric variables for all the observations of the application case for metal surface treatment sector in Fujian, China.

Variables	Mean	SD	Min	Percentiles			Max
				25%	50%	75%	
Wastewater discharge amount (t)	45316.84	89496.35	32.00	6656.25	19493.50	47339.98	639352.95
COD (kg)	4.01	10.75	0.00	0.41	1.21	2.98	100.80
NH_3-N (kg)	0.69	2.40	0.00	0.06	0.11	0.33	21.60
N (kg)	1.08	3.44	0.00	0.09	0.24	0.65	28.80
P (kg)	0.63	1.79	0.00	0.02	0.14	0.45	14.40
Cr (kg)	61.34	163.80	0.00	0.66	5.96	21.38	1088.91
Cr^{VI}(kg)	2.93	7.28	0.00	0.12	0.75	1.80	50.00
Total generation quantity of HW (t)	57.19	123.86	0.00	0.47	6.25	54.53	674.41

Supplementary Table 24. Descriptive statistics of numeric variables for all the observations of the application case for metal surface treatment sector in Guangdong, China.

Variables	Mean	SD	Min	Percentiles			Max
				25%	50%	75%	
Wastewater discharge amount (t)	51406<br>.29	118384.<br>84	22.4<br>0	8783.<br>75	25614.<br>00	56075.<br>00	2812028<br>.00
COD (kg)	4.81	12.05	0.00	0.53	1.80	4.71	225.00
NH_3-N (kg)	0.43	3.07	0.00	0.04	0.13	0.35	90.25
N (kg)	0.60	3.40	0.00	0.05	0.18	0.54	99.28
P (kg)	0.08	0.22	0.00	0.00	0.02	0.05	3.80
Cr (kg)	4.39	15.21	0.00	0.08	0.66	2.19	201.56
Cr^{VI}(kg)	1.94	8.11	0.00	0.04	0.28	1.07	164.00
Total generation quantity of HW (t)	96.60	236.21	0.01	5.00	23.52	80.00	2670.32

Supplementary Table 25. Descriptive statistics of numeric variables for all the observations of the application case for metal surface treatment sector in Hebei, China.

Variables	Mean	SD	Min	Percentiles			Max
				25%	50%	75%	
Wastewater discharge amount (t)	44584.75	101197.76	150.00	4320.00	11711.72	31351.25	954950.00
COD (kg)	4.95	7.99	0.00	0.21	2.30	4.71	39.00
NH_3-N (kg)	0.17	0.20	0.00	0.02	0.10	0.25	0.91
N (kg)	0.67	1.74	0.00	0.04	0.23	0.67	14.40
P (kg)	0.34	0.75	0.00	0.01	0.08	0.34	6.56
Cr (kg)	70.07	209.72	0.00	0.06	5.49	37.37	1892.68
Cr^{VI}(kg)	57.88	194.17	0.00	0.01	0.39	18.39	1714.13
Total generation quantity of HW (t)	91.03	330.32	0.20	2.00	5.05	18.29	2500.00

Supplementary Table 26. Proportion of missing values for 6 routine monitoring indicators of wastewater in the dataset of the studied cases.

Datasets		Data counts	Wastewater discharge amount	COD	pH	NH_3-N	N	P
Datasets for different sectors	OCM	3205	0.00%	4.12%	26.46%	38.91%	87.30%	80.41%
	CPM	1891	0.00%	1.16%	15.02%	18.30%	85.30%	55.00%
	SCP	1176	0.00%	4.17%	24.15%	39.88%	97.87%	90.31%
	STE	28	0.00%	3.57%	0.00%	3.57%	-	42.86%
	SRP	1192	0.00%	61.66%	61.91%	82.05%	91.28%	91.28%
	MWR	833	0.00%	50.30%	52.94%	91.60%	97.00%	95.08%
	MST	6909	0.00%	49.63%	40.86%	70.47%	94.56%	83.92%
	ECM	1531	0.00%	10.12%	49.38%	32.33%	95.36%	89.61%
	BEG	248	0.00%	1.21%	41.13%	27.82%	88.71%	88.71%
	EGU	160	0.00%	14.38%	20.00%	14.38%	95.63%	73.75%
The combined dataset		17173	0.00%	28.93%	36.73%	53.92%	92.15%	81.98%

Note: The data counts are the size of data before outliner rejections.

Supplementary Table 27. Comparison of different missing value imputation approaches on model performances

Models	missing value imputation approaches	R^2	RMSE	MAD	MAE	MAPE	MSE	SSE
The combined model to predict the total generation quantity of HW	Based on the industrial sector median emissions	0.05	2445.83	76.03	231.43	0.49	6.0E+06	2.0E+10
	KNNimpute	0.26	3360.51	75.04	266.99	0.50	1.1E+07	3.7E+10
	MissForest	0.14	1326.03	171.76	326.55	0.57	1.8E+06	5.8E+09
	Based on the contaminant emission intensity	0.87	247.40	60.77	122.28	0.56	6.1E+04	2.0E+08
The combined model to predict the generation quantity of MHW	Based on the industrial sector median emissions	0.68	66.31	1.10	13.36	-	4.4E+03	1.4E+07
	KNNimpute	0.60	76.02	1.60	15.03	-	5.8E+03	1.9E+07
	MissForest	0.55	78.24	3.51	20.00	-	6.1E+03	2.0E+07
	Based on the contaminant emission intensity	0.85	47.50	1.40	13.34	-	2.3E+03	7.4E+06

Supplementary Table 28. Comparison of different data balance approaches on model performances

	Data balance approaches	R^2	RMSE	MAD	MAE	MAPE	MSE	SSE
Total generation quantity of HW	Non data balance	0.80	270.35	70.08	127.04	0.58	7.3E+04	2.4E+08
	Random over-sampling	0.81	281.38	57.71	126.08	0.57	7.9E+04	2.6E+08
	SMOTER	0.85	261.49	63.82	130.13	0.56	6.8E+04	2.3E+08
	SMOGN	0.87	247.40	60.77	122.28	0.56	6.1E+04	2.0E+08
Generation quantity of MHW	Non data balance	0.69	73.39	1.86	14.08	-	5.4E+03	1.8E+07
	Random over-sampling	0.67	67.04	2.73	18.94	-	4.5E+03	1.5E+07
	SMOTER	0.83	48.75	1.68	13.42	-	2.4E+03	7.8E+06
	SMOGN	0.85	47.50	1.40	13.34	-	2.3E+03	7.4E+06

References

1 National Bureau of Statistics of China. *China Statistical Yearbook*. (2021).

2 Yang, M. *et al.* Predicting extraction selectivity of acetic acid in pervaporation by machine learning models with data leakage management. *Environmental Science & Technology* **57**, 5934-5946 (2023). https://doi.org/10.1021/acs.est.2c06382

3 Li, R. *et al.* Large virtual transboundary hazardous waste flows: the case of China. *Environmental Science & Technology* **57**, 8161-8173 (2023). https://doi.org/10.1021/acs.est.2c07962

4 Altman, N. An introduction to kernel and nearest-neighbor nonparametric regression. *American Statistician* **46**, 175-185 (1992). https://doi.org/10.2307/2685209

5 Cover, T.M. & Hart, P.E. Nearest neighbor pattern classification. *Ieee Transactions on Information Theory* **13**, 21-27 (1967). https://doi.org/10.1109/tit.1967.1053964

6 Rousseeuw, P.J. & Vanzomeren, B.C. Unmasking multivariate outliers and leverage points. *Journal of the American Statistical Association* **85**, 633-639 (1990). https://doi.org/10.2307/2289995

7 Fauconnier, C. & Haesbroeck, G. Outliers detection with the minimum covariance determinant estimator in practice. *Statistical Methodology* **6**, 363-379 (2009). https://doi.org/10.1016/j.stamet.2008.12.005

8 He, Z.Y., Xu, X.F. & Deng, S.C. Discovering cluster-based local outliers. *Pattern Recognition Letters* **24**, 1641-1650 (2003). https://doi.org/10.1016/s0167-8655(03)00003-5

9 Gao, Z. Application of cluster based local outlier factor algorithm in antimoney laundering. *Proceedings of the 2009 International Conference on Management and Service Science (MASS)*, 4 pp.-4 pp. (2009). https://doi.org/10.1109/icmss.2009.5302396

10 Goldstein, M. & Dengel, A.R. Histogram-based Outlier Score (HBOS): A fast unsupervised anomaly detection algorithm. *KI-2012: Poster and Demo Track* (2012).

11 Breunig, M.M. *et al.* LOF: Identifying density-based local outliers. *Sigmod Record* **29**, 93-104 (2000). https://doi.org/10.1145/335191.335388

12 Alghushairy, O. *et al.* A Review of local outlier factor algorithms for outlier detection in big data streams. *Big Data and Cognitive Computing* **5** (2021). https://doi.org/10.3390/bdcc5010001

13 Liu, F.T., Ting, K.M. & Zhou, Z.H. Isolation forest. *2008 eighth ieee international conference on data mining*, 413-422 (2008). https://doi.org/10.1109/ICDM.2008.17

14 Tokovarov, M. & Karczmarek, P. A probabilistic generalization of isolation forest. *Information Sciences* **584**, 433-449 (2022). https://doi.org/10.1016/j.ins.2021.10.075

15 Aggarwal, C.C. & Sathe, S. Theoretical foundations and algorithms for outlier ensembles. *SIGKDD Explor. Newsl.* **17**, 24–47 (2015). https://doi.org/10.1145/2830544.2830549

16 Li, Q. *et al.* Determination of corrosion types from electrochemical noise by gradient boosting decision tree method. *International Journal of Electrochemical Science* **14**, 1516-1528 (2019). https://doi.org/10.20964/2019.02.72

17 Hong, W. *et al.* Novel chaotic bat algorithm for forecasting complex motion of floating platforms. *Applied Mathematical Modelling* **72**, 425-443 (2019). https://doi.org/10.1016/j.apm.2019.03.031

18 Yang, H. *et al.* Predicting heavy metal adsorption on soil with machine learning and mapping global distribution of soil adsorption capacities. *Environmental Science & Technology* **55**, 14316-14328 (2021). https://doi.org/10.1021/acs.est.1c02479

19 Altman, N. An introduction to kernel and nearest-neighbor nonparametric regression. *The American Statistician* **46**, 175-185 (1992). https://doi.org/10.1080/00031305.1992.10475879

20 Tyralis, H., Papacharalampous, G. & Langousis, A. A brief review of random forests for water scientists and practitioners and their recent history in water resources. *Water* **11**, 910 (2019). https://doi.org/10.3390/w11050910

21 Cybenko, G. Approximation by superpositions of a sigmoidal function. *Mathematics of Control, Signals and Systems* **2**, 303-314 (1989). https://doi.org/10.1007/BF02551274

22 Taud, H. & Mas, J.F. Multilayer Perceptron (MLP). *Geomatic Approaches for Modeling Land Change Scenarios*, 451-455 (2018). https://doi.org/10.1007/978-3-319-60801-3_27

23 Efron, B. Bootstrap Methods: Another Look at the Jackknife. *Breakthroughs in Statistics: Methodology and Distribution*, 569-593 (1992). https://doi.org/10.1007/978-1-4612-4380-9_41

24 Cheng, H.T. *et al.* Wide & Deep Learning for Recommender Systems. *Proceedings of the 1st Workshop on Deep Learning for Recommender Systems* (2016). https://doi.org/10.48550/arXiv.1606.07792

25 Guo, H. *et al.* DeepFM: A Factorization-Machine based Neural Network for CTR Prediction. *ArXiv* **abs/1703.04247** (2017). https://doi.org/10.48550/arXiv.1703.04247

26 Wang, S., Zhou, W. & Jiang, C. A survey of word embeddings based on deep learning. *Computing* **102**, 717-740 (2020). https://doi.org/10.1007/s00607-019-00768-7

27 Srivastava, N. *et al.* Dropout: a simple way to prevent neural networks from overfitting. *The Journal of Machine Learning Research* **15**, 1929-1958 (2014).

28 He, K. *et al.* Delving Deep into Rectifiers: Surpassing Human-Level Performance on ImageNet Classification. *Proceedings of the 2015 IEEE International Conference on Computer Vision (ICCV)*, 1026–1034 (2015). https://doi.org/10.1109/iccv.2015.123

29 Chicco, D., Warrens, M.J. & Jurman, G. The coefficient of determination R-squared is more informative than SMAPE, MAE, MAPE, MSE and RMSE in regression analysis evaluation. *PeerJ Computer Science* **7**, e623 (2021). https://doi.org/10.7717/peerj-cs.623

30 Zhong, S. *et al.* Machine Learning: new ideas and tools in environmental science and engineering. *Environmental Science & Technology* **55**, 12741-12754 (2021). https://doi.org/10.1021/acs.est.1c01339

31 Troyanskaya, O. *et al.* Missing value estimation methods for DNA microarrays. *Bioinformatics* **17**, 520-525 (2001). https://doi.org/10.1093/bioinformatics/17.6.520

32 Silva, J.d.A. & Hruschka, E.R. An experimental study on the use of nearest neighbor-based imputation algorithms for classification tasks. *Data & Knowledge Engineering* **84**, 47-58 (2013). https://doi.org/10.1016/j.datak.2012.12.006

33 Stekhoven, D.J. & Bühlmann, P. MissForest-non-parametric missing value imputation for mixed-type data. *Bioinformatics* **28**, 112-118 (2012). https://doi.org/10.1093/bioinformatics/btr597

34 Torgo, L. *et al.* SMOTE for Regression. *16th Portuguese Conference on Artificial Intelligence (EPIA)* **8154**, 378-389 (2013).

35 Santoso, B. *et al.* Synthetic over sampling methods for handling class imbalanced problems: A review. *IOP conference series: earth and environmental science* **58**, 012031 (2017). https://doi.org/10.1088/1755-1315/58/1/012031

36 LaPara, T.M. *et al.* Aerobic Biological Treatment of a Pharmaceutical Wastewater: Effect of Temperature on COD Removal and Bacterial Community Development. *Water Research* **35**, 4417-4425 (2001). https://doi.org/10.1016/S0043-1354(01)00178-6

37 Rana, R.S. *et al.* A review on characterization and bioremediation of pharmaceutical industries' wastewater: an Indian perspective. *Applied Water Science* **7**, 1-12 (2017). https://doi.org/10.1007/s13201-014-0225-3

38 Jia, Z. *et al.* Use of pressurized hydrolytic processes as a pretreatment of the wastewater from organophosphate pesticide manufacturing. *Environmental Engineering Science* **24**, 981-986 (2007). https://doi.org/10.1089/EES.2005.0060

39 Kim, J.Y., Oh, S. & Park, Y.K. Overview of biochar production from preservative-treated wood with detailed analysis of biochar characteristics, heavy metals behaviors, and their ecotoxicity. *Journal of Hazardous Materials* **384**, 121356 (2020). https://doi.org/10.1016/j.jhazmat.2019.121356

40 Güneş, E. *et al.* Characterization and treatment alternatives of industrial container and drum cleaning wastewater: Comparison of Fenton-like process and combined coagulation/oxidation processes. *Separation and Purification Technology* **209**, 426-433 (2019). https://doi.org/10.1016/j.seppur.2018.07.060

41 Manyuchi, M.M., Sukdeo, N. & Stinner, W. Potential to remove heavy metals and cyanide from gold mining wastewater using biochar. *Physics and Chemistry of the Earth, Parts A/B/C* **126**, 103110 (2022). https://doi.org/10.1016/j.pce.2022.103110

42 Costa, T.C. *et al.* Evaluation of the technical and environmental feasibility of adsorption process to remove water soluble organics from produced water: A review. *Journal of Petroleum Science and Engineering* **208**, 109360 (2022). https://doi.org/10.1016/j.petrol.2021.109360

43 Rezakazemi, M., Khajeh, A. & Mesbah, M. Membrane filtration of wastewater from gas

and oil production. *Environmental Chemistry Letters* **16**, 367-388 (2018). https://doi.org/10.1007/s10311-017-0693-4

44 Cailleaud, K. *et al.* Investigating predictive tools for refinery effluent hazard assessment using stream mesocosms. *Environmental Toxicology and Chemistry* **38** (2019). https://doi.org/10.1002/etc.4338

45 Park, E.J. *et al.* Effects of gamma-ray treatment on wastewater toxicity from a rubber products factory. *Journal of Radioanalytical and Nuclear Chemistry* **277**, 619-624 (2008). https://doi.org/10.1007/s10967-007-7094-2

46 Ma, F.X. *et al.* Aggregation-induced demulsification technology for the separation of highly emulsified oily wastewater produced in the petrochemical industry. *Journal of Cleaner Production* **374**, 134017 (2022). https://doi.org/10.1016/j.jclepro.2022.134017

47 Abbasi, U. *et al.* Chemidegradation of polychlorinated biphenyls from transformer oil wastewater. *International Journal of Environmental Science and Technology* **19**, 407-420 (2022). https://doi.org/10.1007/s13762-021-03177-6

48 Elmobarak, W.F. *et al.* A Review on the Treatment of Petroleum Refinery Wastewater Using Advanced Oxidation Processes. *Catalysts* **11**, 782 (2021). https://doi.org/10.3390/catal11070782

49 Maiti, D. *et al.* Comprehensive review on wastewater discharged from the coal-related industries–characteristics and treatment strategies. *Water Science and Technology* **79**, 2023-2035 (2019). https://doi.org/10.2166/wst.2019.195

50 Yang, Q. *et al.* Synchronous complete COD reduction for persistent chemical-industrial organic wastewater using the integrated treatment system. *Chemical Engineering Journal* **430**, 133-136 (2022). https://doi.org/10.1016/j.cej.2021.133136

51 Vineetha, M., Matheswaran, M. & Sheeba, K. Photocatalytic colour and COD removal in the distillery effluent by solar radiation. *Solar Energy* **91**, 368-373 (2013). https://doi.org/10.1016/j.solener.2012.09.013

52 Aniyikaiye, T.E. *et al.* Physico-chemical analysis of wastewater discharge from selected paint industries in Lagos, Nigeria. *International journal of environmental research and public health* **16**, 1235 (2019). https://doi.org/10.3390/ijerph16071235

53 Nair, S., Manu, B. & Azhoni, A. Sustainable treatment of paint industry wastewater: Current techniques and challenges. *Journal of Environmental Management* **296**, 113105 (2021). https://doi.org/10.1016/j.jenvman.2021.113105

54 Cao, S. *et al.* Pretreatment Hydrolysis Acidification/Two-Stage AO Combination Process to Treat High-Concentration Resin Production Wastewater. *Water* **14**, 2949 (2022). https://doi.org/10.3390/w14192949

55 Bhanot, P. *et al.* Application of integrated treatment strategies for explosive industry wastewater—A critical review. *Journal of Water Process Engineering* **35**, 101232 (2020). https://doi.org/10.1016/j.jwpe.2020.101232

56 Papadopoulos, K.P. *et al.* Treatment of printing ink wastewater using electrocoagulation. *Journal of environmental management* **237**, 442-448 (2019). https://doi.org/10.1016/j.jenvman.2019.02.080

57 Adamović, S. *et al.* Measurement of copper deposition by electrocoagulation/flotation from waste printing developer. *Measurement* **131**, 288-299 (2019). https://doi.org/10.1016/j.measurement.2018.08.077

58 Stalikas, C.D. *et al.* Degradation of medical X-ray film developing wastewaters by advanced oxidation processes. *Water Research* **35**, 3845-3856 (2001). https://doi.org/10.1016/S0043-1354(01)00107-5

59 Viguri, J. *et al.* Characterization of metal finishing sludges: influence of the pH. *Journal of hazardous materials* **79**, 63-75 (2000). https://doi.org/10.1016/S0304-3894(00)00248-X

60 Xiong, Y. *et al.* Mass balance of heavy metals in a non-operational incinerator residue landfill site in Japan. *Journal of Material Cycles and Waste Management* **22**, 354-364 (2020). https://doi.org/10.1007/s10163-020-00976-w

61 Astrup, T. *et al.* Assessment of long-term pH developments in leachate from waste incineration residues. *Waste management & research* **24**, 491-502 (2006). https://doi.org/10.1177/0734242X06066963

62 Manasfi, T. *et al.* Formation of carbonyl compounds during ozonation of lake water and wastewater: Development of a non-target screening method and quantification of target compounds. *Water Research* **237**, 119751 (2023). https://doi.org/10.1016/j.watres.2023.119751

63 Cesari, C. *et al.* Metal carbonyl clusters of groups 8–10: synthesis and catalysis. *Chemical Society Reviews* **50**, 9503-9539 (2021). https://doi.org/10.1039/D1CS00161B
64 Zhong, S. *et al.* Toxic metals and the risks of sludge from the treatment of wastewater from beryllium smelting. *Chemosphere* **326**, 138439 (2023). https://doi.org/10.1016/j.chemosphere.2023.138439
65 Sawalha, H. *et al.* Wastewater from leather tanning and processing in Palestine: characterization and management aspects. *Journal of environmental management* **251**, 109596 (2019). https://doi.org/10.1016/j.jenvman.2019.109596
66 Abdel Wahaab, R. & Alseroury, F.A. Wastewater treatment: a case study of electronics manufacturing industry. *International Journal of Environmental Science and Technology* **16**, 47-58 (2019). https://doi.org/10.1007/s13762-017-1529-2
67 John, M. *et al.* Purification of heavy metal loaded wastewater from electroplating industry under synthesis of delafossite (ABO2) by "Lt-delafossite process". *Water research* **100**, 98-104 (2016). https://doi.org/10.1016/j.watres.2016.04.071
68 Barakat, M. New trends in removing heavy metals from industrial wastewater. *Arabian journal of chemistry* **4**, 361-377 (2011). https://doi.org/10.1016/j.arabjc.2010.07.019
69 Yao, L. *et al.* Hydrothermal treatment of arsenic sulfide residues from arsenic-bearing acid wastewater. *International Journal of Environmental Research and Public Health* **15**, 1863 (2018). https://doi.org/10.3390/ijerph15091863
70 Staicu, L.C., Nadia, M.C. & Crini, G. Desulfurization: Critical step towards enhanced selenium removal from industrial effluents. *Chemosphere* **172**, 111-119 (2017). https://doi.org/10.1016/j.chemosphere.2016.12.132
71 Volynskii, V. *et al.* Processing of cadmium (II) wastes from battery industry. *Russian journal of applied chemistry* **79**, 1825-1828 (2006). https://doi.org/10.1134/S1070427206110164
72 Guo, X. *et al.* Antimony smelting process generating solid wastes and dust: Characterization and leaching behaviors. *Journal of Environmental Sciences* **26**, 1549-1556 (2014). https://doi.org/10.1016/j.jes.2014.05.022
73 Yao, G. *et al.* Magnetic FeS@Lignin-derived carbon nanocomposites as an efficient adsorbent for multistage collaborative selective recovery of tellurium (IV) from wastewater. *Journal of Environmental Chemical Engineering* **9**, 106135 (2021). https://doi.org/10.1016/j.jece.2021.106135
74 Lothongkum, A.W. *et al.* Simultaneous removal of arsenic and mercury from natural-gas-co-produced water from the Gulf of Thailand using synergistic extractant via HFSLM. *Journal of membrane science* **369**, 350-358 (2011). https://doi.org/10.1016/j.memsci.2010.12.013
75 Ning, L. *et al.* Heavy metal pollution in surface water of Linglong gold mining area, China. *Procedia Environmental Sciences* **10**, 914-917 (2011). https://doi.org/10.1016/j.proenv.2011.09.146
76 Bernardes, A.M., Espinosa, D.C.R. & Tenório, J.S. Recycling of batteries: a review of current processes and technologies. *Journal of Power sources* **130**, 291-298 (2004). https://doi.org/10.1016/j.jpowsour.2003.12.026
77 Zhang, Z. *et al.* Thallium pollution associated with mining of thallium deposits. *Science in China Series D: Earth Sciences* **41**, 75-81 (1998). https://doi.org/10.1007/BF02932424
78 Ma, Y. & Qiu, K. Recovery of lead from lead paste in spent lead acid battery by hydrometallurgical desulfurization and vacuum thermal reduction. *Waste Management* **40**, 151-156 (2015). https://doi.org/10.1016/j.wasman.2015.03.010
79 Kreusch, M. *et al.* Technological improvements in automotive battery recycling. *Resources, conservation and recycling* **52**, 368-380 (2007). https://doi.org/10.1016/j.resconrec.2007.05.004
80 Yu, M. *et al.* Examining regeneration technologies for etching solutions: a critical analysis of the characteristics and potentials. *Journal of Cleaner Production* **113**, 973-980 (2016). https://doi.org/10.1016/j.jclepro.2015.10.131
81 Kuyucak, N. & Akcil, A. Cyanide and removal options from effluents in gold mining and metallurgical processes. *Minerals Engineering* **50**, 13-29 (2013). https://doi.org/10.1016/j.mineng.2013.05.027
82 Shibasaki-Kitakawa, N. *et al.* Production of high quality biodiesel from waste acid oil obtained during edible oil refining using ion-exchange resin catalysts. *Fuel* **139**, 11-17

(2015). https://doi.org/10.1016/j.fuel.2014.08.024
83 Qiu, H. *et al.* From trace to pure: Recovery of scandium from the waste acid of titanium pigment production by solvent extraction. *Process Safety and Environmental Protection* **121**, 118-124 (2019). https://doi.org/10.1016/j.psep.2018.10.027
84 Wei, Q., Ren, X. & Chen, Y. Recovery and separation of sulfuric acid and iron from dilute acidic sulfate effluent and waste sulfuric acid by solvent extraction and stripping. *Journal of Hazardous Materials* **304**, 1-9 (2016). https://doi.org/10.1016/j.jhazmat.2015.10.049
85 Agrawal, A. & Sahu, K.K. An overview of the recovery of acid from spent acidic solutions from steel and electroplating industries. *Journal of hazardous materials* **171**, 61-75 (2009). https://doi.org/10.1016/j.jhazmat.2009.06.099
86 Stocks, C., Wood, J. & Guy, S. Minimisation and recycling of spent acid wastes from galvanizing plants. *Resources, conservation and recycling* **44**, 153-166 (2005). https://doi.org/10.1016/j.resconrec.2004.11.005
87 Kim, J.Y. *et al.* Recovery of phosphoric acid from mixed waste acids of semiconductor industry by diffusion dialysis and vacuum distillation. *Separation and purification technology* **90**, 64-68 (2012). https://doi.org/10.1016/j.seppur.2012.02.013
88 Hariz, I.B. *et al.* Treatment of petroleum refinery sulfidic spent caustic wastes by electrocoagulation. *Separation and Purification Technology* **107**, 150-157 (2013). https://doi.org/10.1016/j.seppur.2013.01.051
89 Wei, Y. *et al.* Comparative study on regenerating sodium hydroxide from the spent caustic by bipolar membrane electrodialysis (BMED) and electro-electrodialysis (EED). *Separation and Purification Technology* **118**, 1-5 (2013). https://doi.org/10.1016/j.seppur.2013.06.025
90 Götz, G. *et al.* Adjustment of the wastewater matrix for optimization of membrane systems applied for water reuse in breweries. *Journal of membrane science* **465**, 68-77 (2014). https://doi.org/10.1016/j.memsci.2014.04.014
91 Kumar, A., Prasad, M.N.V. & Maiti, S. Resource recovery and its waste management. *Environmental Materials and Waste*, 285-305 (2016).
92 Spasiano, D. & Pirozzi, F. Treatments of asbestos containing wastes. *Journal of Environmental Management* **204**, 82-91 (2017). https://doi.org/10.1016/j.jenvman.2017.08.038
93 Colangelo, F. *et al.* Treatment and recycling of asbestos-cement containing waste. *Journal of Hazardous Materials* **195**, 391-397 (2011). https://doi.org/10.1016/j.jhazmat.2011.08.057
94 Cristale, J. *et al.* Occurrence and risk assessment of organophosphorus and brominated flame retardants in the River Aire (UK). *Environmental Pollution* **179**, 194-200 (2013). https://doi.org/10.1016/j.envpol.2013.04.001
95 Zheng, D. *et al.* Pilot-scale integrated membrane system for the treatment of acrylonitrile wastewater. *Desalination* **357**, 215-224 (2015). https://doi.org/10.1016/j.desal.2014.11.026
96 Dai, Y. *et al.* Sequential shape-selective adsorption and photocatalytic transformation of acrylonitrile production wastewater. *Water Research* **85**, 216-225 (2015). https://doi.org/10.1016/j.watres.2015.08.034
97 Han, Y. *et al.* Successful startup of a full-scale acrylonitrile wastewater biological treatment plant (ACN-WWTP) by eliminating the inhibitory effects of toxic compounds on nitrification. *Water science and technology* **69**, 553-559 (2014). https://doi.org/10.2166/wst.2013.744
98 Huang, D. *et al.* Application for acrylonitrile wastewater treatment by new micro-electrolysis ceramic fillers. *Desalination and Water Treatment* **57**, 4420-4428 (2016). https://doi.org/10.1080/19443994.2014.995717
99 Said, K.A.M. *et al.* A review of technologies for the phenolic compounds recovery and phenol removal from wastewater. *Process Safety and Environmental Protection* **151**, 257-289 (2021). https://doi.org/10.1016/j.psep.2021.05.015
100 Hussain, A., Dubey, S.K. & Kumar, V. Kinetic study for aerobic treatment of phenolic wastewater. *Water Resources and industry* **11**, 81-90 (2015). https://doi.org/10.1016/j.wri.2015.05.002
101 Liang, J. *et al.* Long-term microbiota and performance monitoring of a highly efficient propylene oxide co-production methyl tert-butyl ether production wastewater treatment plant. *Journal of Water Process Engineering* **56**, 104376 (2023).

https://doi.org/10.1016/j.jwpe.2023.104376
102 M. Gómez, R. *et al.* Analysis of organic pollutants in sewage sludges from the Valencian community (Spain). *Archives of Environmental Contamination and Toxicology* **52**, 306-316 (2007). https://doi.org/10.1007/s00244-006-0081-8
103 Josiel, M., Javan, G. & Ambrósio, F. Techniques of nickel (II) removal from electroplating industry wastewater: Overview and trends. *Journal of Water Process Engineering* **46**, 102593 (2022). https://doi.org/10.1016/j.jwpe.2022.102593
104 Kumar, V. & Dwivedi, S. A review on accessible techniques for removal of hexavalent Chromium and divalent Nickel from industrial wastewater: Recent research and future outlook. *Journal of Cleaner Production* **295**, 126229 (2021). https://doi.org/10.1016/j.jclepro.2021.126229
105 Gu, H. *et al.* Review on treatment and utilization of barium slag in China. *Journal of Environmental Management* **325**, 116461 (2023). https://doi.org/10.1016/j.jenvman.2022.116461
106 Yu, L. *et al.* Risk assessment of heavy metals in soils and vegetables around non-ferrous metals mining and smelting sites, Baiyin, China. *Journal of Environmental Sciences* **18**, 1124-1134 (2006). https://doi.org/10.1016/S1001-0742(06)60050-8
107 Tomaskova, T. & Bicova, K. Impact of machining on the environment. *Annals of DAAAM and Proceedings of the International DAAAM Symposium* **32**, 623-630 (2021). https://doi.org/10.2507/32nd.daaam.proceedings.088
108 Kamil, S., Zeinab, A. & Çagla, C. Metal swarf and cutting fluid waste management in metal processing industry. *Journal of Metallic Material Research* **1**, 16-26 (2018). https://doi.org/10.30564/jmmr.v1i1.431
109 Kovoor, P.P. *et al.* A study conducted on the impact of effluent waste from machining process on the environment by water analysis. *International Journal of Energy and Environmental Engineering* **3**, 1-12 (2012). https://doi.org/10.1186/2251-6832-3-21
110 Hoffmann, G. *et al.* Thermal treatment of harzardous waste for heavy metal recovery. *Journal of hazardous materials* **145**, 351-357 (2007). https://doi.org/10.1016/j.jhazmat.2007.03.080
111 Shin, S.K. *et al.* Hazardous waste characterization among various thermal processes in South Korea: a comparative analysis. *Journal of hazardous materials* **260**, 157-166 (2013). https://doi.org/10.1016/j.jhazmat.2013.05.022
112 Rajarao, R. *et al.* Novel approach for processing hazardous electronic waste. *Procedia Environmental Sciences* **21**, 33-41 (2014). https://doi.org/10.1016/j.proenv.2014.09.005
113 Duan, H. *et al.* Hazardous waste generation and management in China: A review. *Journal of hazardous materials* **158**, 221-227 (2008). https://doi.org/10.1016/j.jhazmat.2008.01.106
114 Lawrence, K.W., Hung, Y.T. & Nazih, K.S. *Handbook of advanced industrial and hazardous wastes treatment*. (CRC Press, 2009).
115 Huang, K., Guo, J. & Xu, Z. Recycling of waste printed circuit boards: A review of current technologies and treatment status in China. *Journal of hazardous materials* **164**, 399-408 (2009). https://doi.org/10.1016/j.jhazmat.2008.08.051
116 LaDou, J. Printed circuit board industry. *International journal of hygiene and environmental health* **209**, 211-219 (2006). https://doi.org/10.1016/j.ijheh.2006.02.001
117 Khanna, R. *et al.* A novel recycling approach for transforming waste printed circuit boards into a material resource. *Procedia environmental sciences* **21**, 42-54 (2014). https://doi.org/10.1016/j.proenv.2014.09.006
118 Nunno, T. *et al. Toxic waste minimization in the printed circuit board industry*. (William Andrew, 2012).
119 Capello, C., Hellweg, S. & Hungerbühler, K. Environmental Assessment of Waste‐Solvent Treatment Options: Part II: General Rules of Thumb and Specific Recommendations. *Journal of Industrial Ecology* **12**, 111-127 (2008). https://doi.org/10.1111/j.1530-9290.2008.00009.x
120 Guo, Y. *et al.* Microemulsion extraction: An efficient way for simultaneous detoxification and resource recovery of hazardous wastewater containing V (V) and Cr (VI). *Journal of hazardous materials* **386**, 121948 (2020). https://doi.org/10.1016/j.jhazmat.2019.121948
121 Chan, K.H., Malik, M. & Azimi, G. Separation of lithium, nickel, manganese, and cobalt from waste lithium-ion batteries using electrodialysis. *Resources, Conservation and*

Recycling **178**, 106076 (2022). https://doi.org/10.1016/j.resconrec.2021.106076

122 Gharabaghi, M., Irannajad, M. & Azadmehr, A.R. Leaching behavior of cadmium from hazardous waste. *Separation and purification technology* **86**, 9-18 (2012). https://doi.org/10.1016/j.seppur.2011.10.014

123 Pecha, J. *et al.* Technological-economic optimization of enzymatic hydrolysis used for the processing of chrome-tanned leather waste. *Process Safety and Environmental Protection* **152**, 220-229 (2021). https://doi.org/10.1016/j.psep.2021.06.009

124 Jiang, X., Li, Y. & Yan, J. Hazardous waste incineration in a rotary kiln: a review. *Waste Disposal & Sustainable Energy* **1**, 3-37 (2019). https://doi.org/10.1007/s42768-019-00001-3

125 Zhang, Y. *et al.* Treatment of municipal solid waste incineration fly ash: State-of-the-art technologies and future perspectives. *Journal of Hazardous Materials* **411**, 125132 (2021). https://doi.org/10.1016/j.jhazmat.2021.125132

126 Zontek, T.L. & Hall, K.K. Hazardous Materials and Waste Management. *Planning and Managing the Safety System*, 379 (2017).

127 Franciosi, C. *et al.* Measuring maintenance impacts on sustainability of manufacturing industries: from a systematic literature review to a framework proposal. *Journal of Cleaner Production* **260**, 121065 (2020). https://doi.org/10.1016/j.jclepro.2020.121065

128 Ajukumar, V. & Gandhi, O. Evaluation of green maintenance initiatives in design and development of mechanical systems using an integrated approach. *Journal of cleaner production* **51**, 34-46 (2013). https://doi.org/10.1016/j.jclepro.2013.01.010

129 Gardner, M. *et al.* The significance of hazardous chemicals in wastewater treatment works effluents. *Science of the Total Environment* **437**, 363-372 (2012). https://doi.org/10.1016/j.scitotenv.2012.07.086

130 Cieślik, B. & Konieczka, P. A review of phosphorus recovery methods at various steps of wastewater treatment and sewage sludge management. The concept of “no solid waste generation” and analytical methods. *Journal of Cleaner Production* **142**, 1728-1740 (2017). https://doi.org/10.1016/j.jclepro.2016.11.116

131 Zhang, X. *et al.* Adsorption of VOCs onto engineered carbon materials: A review. *Journal of hazardous materials* **338**, 102-123 (2017). https://doi.org/10.1016/j.jhazmat.2017.05.013

132 Nzihou, A. & Stanmore, B. The fate of heavy metals during combustion and gasification of contaminated biomass—a brief review. *Journal of hazardous materials* **256**, 56-66 (2013). https://doi.org/10.1016/j.jhazmat.2013.02.050

133 Silva, L.F. *et al.* Leaching of potential hazardous elements of coal cleaning rejects. *Environmental monitoring and assessment* **175**, 109-126 (2011). https://doi.org/10.1007/s10661-010-1497-1

134 Dudeney, A. *et al.* Management of waste and wastewater from mineral industry processes, especially leaching of sulphide resources: state of the art. *International Journal of Mining, Reclamation and Environment* **27**, 2-37 (2013). https://doi.org/10.1080/17480930.2012.696790

135 Cheng, J. *et al.* Eco-friendly chromium recovery from hazardous chromium-containing vanadium extraction tailings via low-dosage roasting. *Process Safety and Environmental Protection* **164**, 818-826 (2022). https://doi.org/10.1016/j.psep.2022.06.065

136 Tang, L. *et al.* A cleaner process for lead recovery from lead-containing hazardous solid waste and zinc leaching residue via reducing-matting smelting. *Journal of Cleaner Production* **241**, 118328 (2019). https://doi.org/10.1016/j.jclepro.2019.118328

137 Yang, T. *et al.* Solidification/stabilization and separation/extraction treatments of environmental hazardous components in electrolytic manganese residue: a review. *Process Safety and Environmental Protection* **157**, 509-526 (2022). https://doi.org/10.1016/j.psep.2021.10.031

138 Li, H. *et al.* The study of carbon recovery from electrolysis aluminum carbon dust by froth flotation. *Metals* **11**, 145 (2021). https://doi.org/10.3390/met11010145

139 Chalkidis, A. *et al.* Mercury-bearing wastes: Sources, policies and treatment technologies for mercury recovery and safe disposal. *Journal of environmental management* **270**, 110945 (2020). https://doi.org/10.1016/j.jenvman.2020.110945

140 Rodríguez, O. *et al.* Concerns on liquid mercury and mercury-containing wastes: A review of the treatment technologies for the safe storage. *Journal of environmental management* **101**, 197-205 (2012). https://doi.org/10.1016/j.jenvman.2012.02.013

141 Jafarinejad, S. *Petroleum waste treatment and pollution control*. (Butterworth-Heinemann, 2016).
142 Li, J. *et al.* A critical review on energy recovery and non-hazardous disposal of oily sludge from petroleum industry by pyrolysis. *Journal of Hazardous Materials* **406**, 124706 (2021). https://doi.org/10.1016/j.jhazmat.2020.124706
143 Akcil, A. *et al.* A review of metal recovery from spent petroleum catalysts and ash. *Waste management* **45**, 420-433 (2015). https://doi.org/10.1016/j.wasman.2015.07.007
144 Hu, G., Li, J. & Zeng, G. Recent development in the treatment of oily sludge from petroleum industry: a review. *Journal of hazardous materials* **261**, 470-490 (2013). https://doi.org/10.1016/j.jhazmat.2013.07.069
145 Salihoglu, G. Industrial hazardous waste management in Turkey: Current state of the field and primary challenges. *Journal of hazardous materials* **177**, 42-56 (2010). https://doi.org/10.1016/j.jhazmat.2009.11.096
146 Demirbas, A. Waste management, waste resource facilities and waste conversion processes. *Energy Conversion and Management* **52**, 1280-1287 (2011). https://doi.org/10.1016/j.enconman.2010.09.025
147 Ministry of Ecology and Environment of the People's Republic of China. *Self-monitoring technology guidelines for pollution sources— flat glass industry*. Vol. HJ 819-2017(2018).
148 Ministry of Ecology and Environment of the People's Republic of China. *Self-monitoring technology guidelines for pollution sources — Paper industry*. Vol. HJ 821-2017(2017).
149 Ministry of Ecology and Environment of the People's Republic of China. *Self-monitoring technology guidelines for pollution sources— Non-ferrous metal metallurgy industry*. Vol. HJ 989-2018(2018).
150 Ministry of Ecology and Environment of the People's Republic of China. *Self-monitoring technology guidelines for pollution sources —Manufacture of paint and ink*. Vol. HJ 1087—2020(2020).
151 Ministry of Ecology and Environment of the People's Republic of China. *Self-monitoring technology guidelines for pollution sources —Polyvinyl chloride industry*. Vol. HJ 1245—2022(2022).
152 Ministry of Ecology and Environment of the People's Republic of China. *Self-monitoring guidelines for pollution sources—Thermal power generation and boiler*. Vol. HJ 820-2017(2017).
153 Ministry of Ecology and Environment of the People's Republic of China. *Self-monitoring technology guidelines for pollution sources —Onshore oil and gas exploitation and production industry*. (2022).
154 Ministry of Ecology and Environment of the People's Republic of China. *Self-monitoring technology guidelines for pollution sources —Coal processing-production of synthesis gas and liquid fuel*. Vol. HJ 1247—2022(2022).
155 Ministry of Ecology and Environment of the People's Republic of China. *Self-monitoring technology guidelines for pollution sources —Electronics industry*. Vol. HJ 1253—2022(2022).
156 Ministry of Ecology and Environment of the People's Republic of China. *Self-monitoring technology guidelines for pollution sources—Chemical fibers manufacturing industry*. Vol. HJ 1139-2020(2020).
157 Ministry of Ecology and Environment of the People's Republic of China. *Self-monitoring technology guidelines for pollution sources—Inorganic chemical industry*. Vol. HJ 1138—2020(2020).
158 Ministry of Ecology and Environment of the People's Republic of China. *Self-monitoring technology guidelines for pollution sources — Iron and steel industry and coking chemical industry*. Vol. HJ 878-2017(2017).
159 Ministry of Ecology and Environment of the People's Republic of China. *Self-monitoring technology guidelines for pollution sources-Pharmaceutical industry chemical synthesis products category*. Vol. HJ 883-2017(2017).
160 Ministry of Ecology and Environment of the People's Republic of China. *Self-monitoring technology guidelines for pollution sources Phosphatic, potassic, compound, organic and microbial fertilizer*. Vol. HJ 1088-2020(2020).
161 Ministry of Ecology and Environment of the People's Republic of China. *Self-monitoring technology guidelines for pollution sources— Electroplating industry*. Vol. HJ 985-

2018(2018).

162 Ministry of Ecology and Environment of the People's Republic of China. *Self-monitoring technology guidelines for pollution sources — Coating*. Vol. HJ 1086-2020(2020).

163 Ministry of Ecology and Environment of the People's Republic of China. *Self-monitoring technology guidelines for pollution sources — Rubber and plastic products industry*. Vol. HJ 1207—2021(2021).

164 Ministry of Ecology and Environment of the People's Republic of China. *Self-monitoring technology guidelines for pollution sources —Non-ferrous metal metallurgy industry—secondary non-ferrous metal*. Vol. HJ 1208—2021(2021).

165 Ministry of Ecology and Environment of the People's Republic of China. *Self-monitoring technology guidelines for pollution sources —Metal foundry industry*. Vol. HJ 1251—2022(2022).

166 Ministry of Ecology and Environment of the People's Republic of China. *Self-monitoring technology guidelines for pollution sources — Battery industry*. Vol. HJ 1204—2021(2021).

167 Ministry of Ecology and Environment of the People's Republic of China. *Self-monitoring technology guidelines for pollution sources —Printing industry*. Vol. HJ 1246—2022(2022).

168 Ministry of Ecology and Environment of the People's Republic of China. *Self-monitoring technology guidelines for pollution sources-Textile and dyeing industry*. Vol. HJ 879-2017(2017).

169 Ministry of Ecology and Environment of the People's Republic of China. *Self-monitoring technology guidelines for pollution sources —Ceramics industry*. Vol. HJ 1255-2022(2022).

170 Ministry of Ecology and Environment of the People's Republic of China. *Self-monitoring technology guidelines for pollution sources—Pesticide manufacture*. Vol. HJ 987-2018(2018).

171 Ministry of Ecology and Environment of the People's Republic of China. *Self-monitoring technology guidelines for pollution sources— Petroleum chemistry industry*. Vol. HJ 947-2018(2018).

172 Ministry of Ecology and Environment of the People's Republic of China. *Chinese National List of Hazardous Wastes*. (2021).

173 U.S. Environmental Protection Agency. *Identification and listing of hazardous waste*. (1991).

174 EU. Commission. *Commission notice on technical guidance on the classification of waste*. (2018).
